# Supplementary material for: Sorting at embryonic boundaries requires high heterotypic interfacial tension
Source: Nat Commun. 2017 Jul 31;8:157. doi: 10.1038/s41467-017-00146-x (PMC5537356; doi:10.1038/s41467-017-00146-x)
Supplement: Supplementary file 2 — Supplementary Software 1 [file 41467_2017_146_MOESM2_ESM.zip › PottsModel/SrcPottsModel/doc/allclasses-frame.html]

All Classes


# All Classes

- AObservable
- AreaEnergyFunction
- AreaEnergyStatistic
- AreaStatistic
- BoundarySimulation
- CartesianCoordinates
- Cell
- Cell.CellType
- Cell.Energy
- Cell.State
- CellCoordinatesCSVStatistic
- CellDisplay
- CellEnergyStatistics
- CellSet
- CellShapeCSVLabel
- CellShapeCSVStatistic
- CellStatistic
- ColorScale
- CommandLineSimulation
- ConfigurationInformationPanel
- ConfigurationPanel
- ConfigurationStartMenu
- Console
- Constants
- Constants.Model
- Coordinates
- CSVStatistic
- DefaultSimulation
- DispersionIndex
- DITHSimulation
- EndoSimulation
- EnergyFunction
- EnergyFunction.Energy
- EnergyManager
- EnergyStatistic
- EngineObserverPanel
- FiniteVector
- FourByFourSimulation
- GenerateRandomLattices
- GranerGlazierSimulation
- HalfLine
- HBLStatistic
- Hexagon
- HexagonPixelDisplay
- HexagonPositionManager
- HMDStatistic
- HSLColor
- IEnergyFunction
- InteractionEnergyStatistic
- InterfacialTensionEnergyFunction
- IO
- IObservable
- IPositionManager
- IPositionManager.Position
- IsoperimetricQuotientStatistic
- JavaVersion
- KnowsConstants
- Lattice
- Lattice.State
- Lattice.Type
- LatticeUtilities
- NegativeControl
- Observer
- Pair
- PerimeterEnergyFunction
- PerimeterStatistic
- Pixel
- Pixel.PixelType
- PixelDisplay
- PixelInteractionEnergyFunction
- PixelShape
- PixelShape.Edge
- PixelShape.Type
- PlotPanel
- PottsCanvas
- PottsEngine
- PottsEngine.State
- PottsFrame
- PottsFrame.Action
- PottsLogger
- PottsToolbar
- Simulation
- SingleCell
- SnapshotManager
- Square
- SquareCell
- SquarePixelDisplay
- SquarePositionManager
- Statistic
- Statistic.Utils
- StatisticsManager
- StatusBar
- TypeSpecificAreaStatistic
- TypeSpecificCellStatistic
- TypeSpecificNearestNeighborStatistic
- TypeSpecificNumNeighborsStatistic
- TypeSpecificPercentIsolatedCellStatistic
- TypeSpecificPerimeterStatistic
- TypeSpecificStatistic
- Utils
- Utils
- Utils.EnergyTracker
- Vector
- VectorTests
- VectorUtils
