# Supplementary material for: Sorting at embryonic boundaries requires high heterotypic interfacial tension
Source: Nat Commun. 2017 Jul 31;8:157. doi: 10.1038/s41467-017-00146-x (PMC5537356; doi:10.1038/s41467-017-00146-x)
Supplement: Supplementary file 2 — Supplementary Software 1 [file 41467_2017_146_MOESM2_ESM.zip › PottsModel/SrcPottsModel/doc/constant-values.html]

Constant Field Values


JavaScript is disabled on your browser.


Skip navigation links


- Overview
- Package
- Class
- Use
- Tree
- Deprecated
- Index
- Help

- Prev
- Next

- Frames
- No Frames

- All Classes

# Constant Field Values

## Contents

- engine.\*
- model.\*

## engine.\*

- engine.PottsEngine

  | Modifier and Type | Constant Field | Value |
  |  |  |  |
  | --- | --- | --- |
  | `public static final java.lang.String` | `cNotificationSource` | `"potts.engine"` |
- engine.Statistic

  | Modifier and Type | Constant Field | Value |
  |  |  |  |
  | --- | --- | --- |
  | `public static final int` | `DEFAULT_FREQUENCY` | `1` |


## model.\*

- model.Cell

  | Modifier and Type | Constant Field | Value |
  |  |  |  |
  | --- | --- | --- |
  | `public final double` | `cAreaUnit` | `1.0` |
  | `public static final java.lang.String` | `cNotificationSource` | `"potts.cell"` |
- model.Lattice

  | Modifier and Type | Constant Field | Value |
  |  |  |  |
  | --- | --- | --- |
  | `public static final java.lang.String` | `cDefaultConfigFile` | `"config/config.properties"` |
  | `public static final java.lang.String` | `cNotificationSource` | `"model.lattice"` |

Skip navigation links


- Overview
- Package
- Class
- Use
- Tree
- Deprecated
- Index
- Help

- Prev
- Next

- Frames
- No Frames

- All Classes
