# Supplementary material for: Sorting at embryonic boundaries requires high heterotypic interfacial tension
Source: Nat Commun. 2017 Jul 31;8:157. doi: 10.1038/s41467-017-00146-x (PMC5537356; doi:10.1038/s41467-017-00146-x)
Supplement: Supplementary file 2 — Supplementary Software 1 [file 41467_2017_146_MOESM2_ESM.zip › PottsModel/SrcPottsModel/doc/engine/CellShapeCSVStatistic.html]

CellShapeCSVStatistic


JavaScript is disabled on your browser.


Skip navigation links


- Overview
- Package
- Class
- Use
- Tree
- Deprecated
- Index
- Help

- Prev Class
- Next Class

- Frames
- No Frames

- All Classes

- Summary:
- Nested |
- Field |
- Constr |
- Method

- Detail:
- Field |
- Constr |
- Method


engine

## Class CellShapeCSVStatistic

- java.lang.Object
- - engine.Statistic
  - - engine.CSVStatistic<CellShapeCSVLabel>
    - - engine.CellShapeCSVStatistic

- ---

    

  ```
  public class CellShapeCSVStatistic
  extends CSVStatistic<CellShapeCSVLabel>
  ```

  Tracks cell shape statistics (area, perimeter, center of mass) in a separate csv file for \*\*EACH CELL\*\*
  Therefore, this statistic is memory intensive and only recommended for a very limited number of MCS.

  Author:
  :   eleyine

- - ### Nested Class Summary

    - ### Nested classes/interfaces inherited from class engine.Statistic

      `Statistic.Utils`
  - ### Field Summary

    - ### Fields inherited from class engine.Statistic

      `DEFAULT_FREQUENCY`
  - ### Constructor Summary

    Constructors

    | Constructor and Description |
    | `CellShapeCSVStatistic(PottsEngine engine, int frequency)` |
  - ### Method Summary

    All Methods Instance Methods Concrete Methods

    | Modifier and Type | Method and Description |
    | `CellShapeCSVLabel[]` | `getColumnLabelEnums()` |
    | `java.lang.String` | `getCSVRow(PottsEngine pEngine, Cell pCell)` |
    | `java.lang.String` | `getFilenamePrefix()` |
    | `boolean` | `isSpinAttemptsObserved()` |
    | `boolean` | `isStateObservable(PottsEngine.State pState)` |

    - ### Methods inherited from class engine.CSVStatistic

      `getColumnLabels, getSeriesNames, getYAxisLabel, isDisplayed, observe`
    - ### Methods inherited from class engine.Statistic

      `addToManagerStatistics, attachPlotPanel, getAxis, getEngine, getFrequency, getLastValues, isAreaRendered, setRepeatFrequency, wrapUp`
    - ### Methods inherited from class java.lang.Object

      `equals, getClass, hashCode, notify, notifyAll, toString, wait, wait, wait`

- - ### Constructor Detail


    - #### CellShapeCSVStatistic

      ```
      public CellShapeCSVStatistic(PottsEngine engine,
                                   int frequency)
      ```
  - ### Method Detail


    - #### getCSVRow

      ```
      public java.lang.String getCSVRow(PottsEngine pEngine,
                                        Cell pCell)
      ```

      Specified by:
      :   `getCSVRow` in class `CSVStatistic<CellShapeCSVLabel>`


    - #### getColumnLabelEnums

      ```
      public CellShapeCSVLabel[] getColumnLabelEnums()
      ```

      Specified by:
      :   `getColumnLabelEnums` in class `CSVStatistic<CellShapeCSVLabel>`


    - #### getFilenamePrefix

      ```
      public java.lang.String getFilenamePrefix()
      ```

      Specified by:
      :   `getFilenamePrefix` in class `CSVStatistic<CellShapeCSVLabel>`


    - #### isStateObservable

      ```
      public boolean isStateObservable(PottsEngine.State pState)
      ```

      Specified by:
      :   `isStateObservable` in class `CSVStatistic<CellShapeCSVLabel>`


    - #### isSpinAttemptsObserved

      ```
      public boolean isSpinAttemptsObserved()
      ```

      Specified by:
      :   `isSpinAttemptsObserved` in class `CSVStatistic<CellShapeCSVLabel>`


Skip navigation links


- Overview
- Package
- Class
- Use
- Tree
- Deprecated
- Index
- Help

- Prev Class
- Next Class

- Frames
- No Frames

- All Classes

- Summary:
- Nested |
- Field |
- Constr |
- Method

- Detail:
- Field |
- Constr |
- Method
