# Supplementary material for: Sorting at embryonic boundaries requires high heterotypic interfacial tension
Source: Nat Commun. 2017 Jul 31;8:157. doi: 10.1038/s41467-017-00146-x (PMC5537356; doi:10.1038/s41467-017-00146-x)
Supplement: Supplementary file 2 — Supplementary Software 1 [file 41467_2017_146_MOESM2_ESM.zip › PottsModel/SrcPottsModel/doc/engine/CellStatistic.html]

CellStatistic


JavaScript is disabled on your browser.


Skip navigation links


- Overview
- Package
- Class
- Use
- Tree
- Deprecated
- Index
- Help

- Prev Class
- Next Class

- Frames
- No Frames

- All Classes

- Summary:
- Nested |
- Field |
- Constr |
- Method

- Detail:
- Field |
- Constr |
- Method


engine

## Class CellStatistic

- java.lang.Object
- - engine.Statistic
  - - engine.CellStatistic

- Direct Known Subclasses:
  :   AreaEnergyStatistic, AreaStatistic, InteractionEnergyStatistic, PerimeterStatistic

  ---

    

  ```
  public abstract class CellStatistic
  extends Statistic
  ```

- - ### Nested Class Summary

    - ### Nested classes/interfaces inherited from class engine.Statistic

      `Statistic.Utils`
  - ### Field Summary

    - ### Fields inherited from class engine.Statistic

      `DEFAULT_FREQUENCY`
  - ### Constructor Summary

    Constructors

    | Constructor and Description |
    | `CellStatistic(PottsEngine engine, java.lang.String yAxisLabel)` |
    | `CellStatistic(PottsEngine engine, java.lang.String yAxisLabel, int frequency)` |
  - ### Method Summary

    All Methods Instance Methods Concrete Methods

    | Modifier and Type | Method and Description |
    | `java.lang.String[]` | `getSeriesNames()` |

    - ### Methods inherited from class engine.Statistic

      `addToManagerStatistics, attachPlotPanel, getAxis, getEngine, getFrequency, getLastValues, getYAxisLabel, isAreaRendered, isDisplayed, isSpinAttemptsObserved, observe, setRepeatFrequency, wrapUp`
    - ### Methods inherited from class java.lang.Object

      `equals, getClass, hashCode, notify, notifyAll, toString, wait, wait, wait`

- - ### Constructor Detail


    - #### CellStatistic

      ```
      public CellStatistic(PottsEngine engine,
                           java.lang.String yAxisLabel)
      ```


    - #### CellStatistic

      ```
      public CellStatistic(PottsEngine engine,
                           java.lang.String yAxisLabel,
                           int frequency)
      ```
  - ### Method Detail


    - #### getSeriesNames

      ```
      public java.lang.String[] getSeriesNames()
      ```

      Specified by:
      :   `getSeriesNames` in class `Statistic`


Skip navigation links


- Overview
- Package
- Class
- Use
- Tree
- Deprecated
- Index
- Help

- Prev Class
- Next Class

- Frames
- No Frames

- All Classes

- Summary:
- Nested |
- Field |
- Constr |
- Method

- Detail:
- Field |
- Constr |
- Method
