# Supplementary material for: Sorting at embryonic boundaries requires high heterotypic interfacial tension
Source: Nat Commun. 2017 Jul 31;8:157. doi: 10.1038/s41467-017-00146-x (PMC5537356; doi:10.1038/s41467-017-00146-x)
Supplement: Supplementary file 2 — Supplementary Software 1 [file 41467_2017_146_MOESM2_ESM.zip › PottsModel/SrcPottsModel/doc/engine/class-use/CellShapeCSVLabel.html]

Uses of Class engine.CellShapeCSVLabel


JavaScript is disabled on your browser.


Skip navigation links


- Overview
- Package
- Class
- Use
- Tree
- Deprecated
- Index
- Help

- Prev
- Next

- Frames
- No Frames

- All Classes

## Uses of Class engine.CellShapeCSVLabel

- Packages that use CellShapeCSVLabel

  | Package | Description |
  |  |  |
  | --- | --- |
  | engine |  |
- - ### Uses of CellShapeCSVLabel in engine

    Methods in engine that return CellShapeCSVLabel

    | Modifier and Type | Method and Description |
    |  |  |
    | --- | --- |
    | `CellShapeCSVLabel[]` | CellShapeCSVStatistic.`getColumnLabelEnums()` |
    | `CellShapeCSVLabel[]` | CellCoordinatesCSVStatistic.`getColumnLabelEnums()` |
    | `static CellShapeCSVLabel` | CellShapeCSVLabel.`valueOf(java.lang.String name)` Returns the enum constant of this type with the specified name. |
    | `static CellShapeCSVLabel[]` | CellShapeCSVLabel.`values()` Returns an array containing the constants of this enum type, in the order they are declared. |

Skip navigation links


- Overview
- Package
- Class
- Use
- Tree
- Deprecated
- Index
- Help

- Prev
- Next

- Frames
- No Frames

- All Classes
