# Supplementary material for: Sorting at embryonic boundaries requires high heterotypic interfacial tension
Source: Nat Commun. 2017 Jul 31;8:157. doi: 10.1038/s41467-017-00146-x (PMC5537356; doi:10.1038/s41467-017-00146-x)
Supplement: Supplementary file 2 — Supplementary Software 1 [file 41467_2017_146_MOESM2_ESM.zip › PottsModel/SrcPottsModel/doc/engine/class-use/PottsEngine.html]

Uses of Class engine.PottsEngine


JavaScript is disabled on your browser.


Skip navigation links


- Overview
- Package
- Class
- Use
- Tree
- Deprecated
- Index
- Help

- Prev
- Next

- Frames
- No Frames

- All Classes

## Uses of Class engine.PottsEngine

- Packages that use PottsEngine

  | Package | Description |
  |  |  |
  | --- | --- |
  | engine |  |
  | gui |  |
- - ### Uses of PottsEngine in engine

    Methods in engine that return PottsEngine

    | Modifier and Type | Method and Description |
    |  |  |
    | --- | --- |
    | `PottsEngine` | Statistic.`getEngine()` |
    | `static PottsEngine` | CommandLineSimulation.`parseArguments(java.lang.String[] args)` |

    Methods in engine with parameters of type PottsEngine

    | Modifier and Type | Method and Description |
    |  |  |
    | --- | --- |
    | `abstract java.lang.String` | CSVStatistic.`getCSVRow(PottsEngine paramPottsEngine, Cell paramCell)` |
    | `java.lang.String` | CellShapeCSVStatistic.`getCSVRow(PottsEngine pEngine, Cell pCell)` |
    | `java.lang.String` | CellCoordinatesCSVStatistic.`getCSVRow(PottsEngine pEngine, Cell pCell)` |

    Constructors in engine with parameters of type PottsEngine

    | Constructor and Description |
    |  |
    | --- |
    | `AreaEnergyStatistic(PottsEngine engine)` |
    | `AreaStatistic(PottsEngine engine)` |
    | `AreaStatistic(PottsEngine engine, int frequency)` |
    | `CellCoordinatesCSVStatistic(PottsEngine engine, int frequency)` |
    | `CellEnergyStatistics(PottsEngine engine)` |
    | `CellEnergyStatistics(PottsEngine engine, int frequency)` |
    | `CellShapeCSVStatistic(PottsEngine engine, int frequency)` |
    | `CellStatistic(PottsEngine engine, java.lang.String yAxisLabel)` |
    | `CellStatistic(PottsEngine engine, java.lang.String yAxisLabel, int frequency)` |
    | `CSVStatistic(PottsEngine engine, int frequency)` |
    | `DispersionIndex(PottsEngine engine, Cell.CellType[] pCellTypes)` |
    | `DispersionIndex(PottsEngine engine, Cell.CellType[] pCellTypes, int frequency)` |
    | `EnergyStatistic(PottsEngine engine)` |
    | `EnergyStatistic(PottsEngine engine, int frequency)` |
    | `HBLStatistic(PottsEngine engine)` |
    | `HBLStatistic(PottsEngine engine, int frequency)` |
    | `HMDStatistic(PottsEngine pEngine, Cell.CellType[] pCellTypes, int pFrequency)` |
    | `InteractionEnergyStatistic(PottsEngine engine)` |
    | `InteractionEnergyStatistic(PottsEngine engine, int frequency)` |
    | `IsoperimetricQuotientStatistic(PottsEngine pEngine, Cell.CellType[] pCellTypes, int pFrequency)` |
    | `PerimeterStatistic(PottsEngine engine)` |
    | `PerimeterStatistic(PottsEngine engine, int frequency)` |
    | `Statistic(PottsEngine engine, java.lang.String yAxisLabel)` |
    | `Statistic(PottsEngine engine, java.lang.String yAxisLabel, int frequency)` |
    | `StatisticsManager(PottsEngine e)` |
    | `TypeSpecificAreaStatistic(PottsEngine pEngine, Cell.CellType[] pCellTypes, int pFrequency)` |
    | `TypeSpecificCellStatistic(PottsEngine pEngine, java.lang.String pYAxisLabel, Cell.CellType[] pCellTypes, int pFrequency)` |
    | `TypeSpecificNearestNeighborStatistic(PottsEngine pEngine, Cell.CellType[] pCellTypes, int pFrequency)` |
    | `TypeSpecificNearestNeighborStatistic(PottsEngine pEngine, Cell.CellType[] pCellTypes, int pFrequency, int pNumNeighbors)` |
    | `TypeSpecificNumNeighborsStatistic(PottsEngine pEngine, java.lang.String pYAxisLabel, Cell.CellType[] pCellTypes, int pFrequency)` |
    | `TypeSpecificPercentIsolatedCellStatistic(PottsEngine pEngine, java.lang.String pYAxisLabel, Cell.CellType[] pCellTypes, int pFrequency)` |
    | `TypeSpecificPerimeterStatistic(PottsEngine pEngine, Cell.CellType[] pCellTypes, int pFrequency)` |
    | `TypeSpecificStatistic(PottsEngine pEngine, java.lang.String pYAxisLabel, Cell.CellType[] pCellTypes, int pFrequency)` |
  - ### Uses of PottsEngine in gui

    Constructors in gui with parameters of type PottsEngine

    | Constructor and Description |
    |  |
    | --- |
    | `ConfigurationInformationPanel(PottsEngine pEngine)` |
    | `PottsFrame(PottsEngine pEngine)` |

Skip navigation links


- Overview
- Package
- Class
- Use
- Tree
- Deprecated
- Index
- Help

- Prev
- Next

- Frames
- No Frames

- All Classes
