# Supplementary material for: Sorting at embryonic boundaries requires high heterotypic interfacial tension
Source: Nat Commun. 2017 Jul 31;8:157. doi: 10.1038/s41467-017-00146-x (PMC5537356; doi:10.1038/s41467-017-00146-x)
Supplement: Supplementary file 2 — Supplementary Software 1 [file 41467_2017_146_MOESM2_ESM.zip › PottsModel/SrcPottsModel/doc/engine/class-use/PottsEngine.State.html]

Uses of Class engine.PottsEngine.State


JavaScript is disabled on your browser.


Skip navigation links


- Overview
- Package
- Class
- Use
- Tree
- Deprecated
- Index
- Help

- Prev
- Next

- Frames
- No Frames

- All Classes

## Uses of Class engine.PottsEngine.State

- Packages that use PottsEngine.State

  | Package | Description |
  |  |  |
  | --- | --- |
  | engine |  |
- - ### Uses of PottsEngine.State in engine

    Methods in engine that return PottsEngine.State

    | Modifier and Type | Method and Description |
    |  |  |
    | --- | --- |
    | `static PottsEngine.State` | PottsEngine.State.`valueOf(java.lang.String name)` Returns the enum constant of this type with the specified name. |
    | `static PottsEngine.State[]` | PottsEngine.State.`values()` Returns an array containing the constants of this enum type, in the order they are declared. |

    Methods in engine with parameters of type PottsEngine.State

    | Modifier and Type | Method and Description |
    |  |  |
    | --- | --- |
    | `abstract boolean` | CSVStatistic.`isStateObservable(PottsEngine.State paramState)` |
    | `boolean` | CellShapeCSVStatistic.`isStateObservable(PottsEngine.State pState)` |
    | `boolean` | CellCoordinatesCSVStatistic.`isStateObservable(PottsEngine.State pState)` |
    | `void` | TypeSpecificStatistic.`observe(PottsEngine.State engineState)` |
    | `void` | TypeSpecificPerimeterStatistic.`observe(PottsEngine.State engineState)` |
    | `void` | TypeSpecificNumNeighborsStatistic.`observe(PottsEngine.State engineState)` |
    | `void` | TypeSpecificNearestNeighborStatistic.`observe(PottsEngine.State engineState)` |
    | `void` | TypeSpecificAreaStatistic.`observe(PottsEngine.State engineState)` |
    | `abstract void` | Statistic.`observe(PottsEngine.State paramState)` Observe values during MCS. |
    | `void` | PerimeterStatistic.`observe(PottsEngine.State engineState)` |
    | `void` | IsoperimetricQuotientStatistic.`observe(PottsEngine.State engineState)` |
    | `void` | InteractionEnergyStatistic.`observe(PottsEngine.State engineState)` |
    | `void` | HMDStatistic.`observe(PottsEngine.State engineState)` |
    | `void` | HBLStatistic.`observe(PottsEngine.State engineState)` |
    | `void` | EnergyStatistic.`observe(PottsEngine.State engineState)` |
    | `void` | DispersionIndex.`observe(PottsEngine.State engineState)` |
    | `void` | CSVStatistic.`observe(PottsEngine.State engineState)` |
    | `void` | CellEnergyStatistics.`observe(PottsEngine.State engineState)` |
    | `void` | AreaStatistic.`observe(PottsEngine.State engineState)` |
    | `void` | AreaEnergyStatistic.`observe(PottsEngine.State engineState)` |

Skip navigation links


- Overview
- Package
- Class
- Use
- Tree
- Deprecated
- Index
- Help

- Prev
- Next

- Frames
- No Frames

- All Classes
