# Supplementary material for: Sorting at embryonic boundaries requires high heterotypic interfacial tension
Source: Nat Commun. 2017 Jul 31;8:157. doi: 10.1038/s41467-017-00146-x (PMC5537356; doi:10.1038/s41467-017-00146-x)
Supplement: Supplementary file 2 — Supplementary Software 1 [file 41467_2017_146_MOESM2_ESM.zip › PottsModel/SrcPottsModel/doc/engine/class-use/Statistic.html]

Uses of Class engine.Statistic


JavaScript is disabled on your browser.


Skip navigation links


- Overview
- Package
- Class
- Use
- Tree
- Deprecated
- Index
- Help

- Prev
- Next

- Frames
- No Frames

- All Classes

## Uses of Class engine.Statistic

- Packages that use Statistic

  | Package | Description |
  |  |  |
  | --- | --- |
  | engine |  |
  | gui |  |
- - ### Uses of Statistic in engine

    Subclasses of Statistic in engine

    | Modifier and Type | Class and Description |
    |  |  |
    | --- | --- |
    | `class` | `AreaEnergyStatistic` |
    | `class` | `AreaStatistic` |
    | `class` | `CellCoordinatesCSVStatistic` |
    | `class` | `CellEnergyStatistics` |
    | `class` | `CellShapeCSVStatistic` Tracks cell shape statistics (area, perimeter, center of mass) in a separate csv file for \*\*EACH CELL\*\* Therefore, this statistic is memory intensive and only recommended for a very limited number of MCS. |
    | `class` | `CellStatistic` |
    | `class` | `CSVStatistic<LabelEnum extends java.lang.Enum<LabelEnum>>` |
    | `class` | `DispersionIndex` Dispersion index statistic measured for each cell type using 10x10 quadrats. |
    | `class` | `EnergyStatistic` |
    | `class` | `HBLStatistic` |
    | `class` | `HMDStatistic` Homotypic Minimal Distance statistic. |
    | `class` | `InteractionEnergyStatistic` |
    | `class` | `IsoperimetricQuotientStatistic` |
    | `class` | `PerimeterStatistic` |
    | `class` | `TypeSpecificAreaStatistic` |
    | `class` | `TypeSpecificCellStatistic` |
    | `class` | `TypeSpecificNearestNeighborStatistic` |
    | `class` | `TypeSpecificNumNeighborsStatistic` |
    | `class` | `TypeSpecificPercentIsolatedCellStatistic` |
    | `class` | `TypeSpecificPerimeterStatistic` |
    | `class` | `TypeSpecificStatistic` |

    Method parameters in engine with type arguments of type Statistic

    | Modifier and Type | Method and Description |
    |  |  |
    | --- | --- |
    | `void` | Statistic.`addToManagerStatistics(java.util.List<Statistic> statsList)` |
  - ### Uses of Statistic in gui

    Methods in gui with parameters of type Statistic

    | Modifier and Type | Method and Description |
    |  |  |
    | --- | --- |
    | `org.jfree.data.xy.XYSeries` | PlotPanel.`getSeries(Statistic s)` |
    | `void` | PlotPanel.`observe(Statistic s, double x, double y, java.lang.String seriesName)` |

    Constructor parameters in gui with type arguments of type Statistic

    | Constructor and Description |
    |  |
    | --- |
    | `PlotPanel(java.util.List<Statistic> aStatistics)` Constructs a new demonstration application. |

Skip navigation links


- Overview
- Package
- Class
- Use
- Tree
- Deprecated
- Index
- Help

- Prev
- Next

- Frames
- No Frames

- All Classes
