# Supplementary material for: Sorting at embryonic boundaries requires high heterotypic interfacial tension
Source: Nat Commun. 2017 Jul 31;8:157. doi: 10.1038/s41467-017-00146-x (PMC5537356; doi:10.1038/s41467-017-00146-x)
Supplement: Supplementary file 2 — Supplementary Software 1 [file 41467_2017_146_MOESM2_ESM.zip › PottsModel/SrcPottsModel/doc/engine/class-use/Utils.EnergyTracker.html]

Uses of Class engine.Utils.EnergyTracker


JavaScript is disabled on your browser.


Skip navigation links


- Overview
- Package
- Class
- Use
- Tree
- Deprecated
- Index
- Help

- Prev
- Next

- Frames
- No Frames

- All Classes

## Uses of Class engine.Utils.EnergyTracker

- Packages that use Utils.EnergyTracker

  | Package | Description |
  |  |  |
  | --- | --- |
  | engine |  |
  | model |  |
- - ### Uses of Utils.EnergyTracker in engine

    Fields in engine declared as Utils.EnergyTracker

    | Modifier and Type | Field and Description |
    |  |  |
    | --- | --- |
    | `Utils.EnergyTracker` | PottsEngine.StateVariables.`aDeltaEnergyTracker` |

    Methods in engine with parameters of type Utils.EnergyTracker

    | Modifier and Type | Method and Description |
    |  |  |
    | --- | --- |
    | `void` | StatisticsManager.`setTracker(Utils.EnergyTracker t)` |
    | `void` | EnergyStatistic.`setTracker(Utils.EnergyTracker t)` |
  - ### Uses of Utils.EnergyTracker in model

    Methods in model that return Utils.EnergyTracker

    | Modifier and Type | Method and Description |
    |  |  |
    | --- | --- |
    | `Utils.EnergyTracker` | EnergyManager.`getTracker()` Makes new tacker based on energy function types. |

    Methods in model with parameters of type Utils.EnergyTracker

    | Modifier and Type | Method and Description |
    |  |  |
    | --- | --- |
    | `void` | EnergyManager.`setTracker(Utils.EnergyTracker t)` |

Skip navigation links


- Overview
- Package
- Class
- Use
- Tree
- Deprecated
- Index
- Help

- Prev
- Next

- Frames
- No Frames

- All Classes
