# Supplementary material for: Sorting at embryonic boundaries requires high heterotypic interfacial tension
Source: Nat Commun. 2017 Jul 31;8:157. doi: 10.1038/s41467-017-00146-x (PMC5537356; doi:10.1038/s41467-017-00146-x)
Supplement: Supplementary file 2 — Supplementary Software 1 [file 41467_2017_146_MOESM2_ESM.zip › PottsModel/SrcPottsModel/doc/engine/CommandLineSimulation.Option.html]

CommandLineSimulation.Option


---


|  |  |  |  |  |  |  |  |  |  |  |
| --- | --- | --- | --- | --- | --- | --- | --- | --- | --- | --- |
| |  |  |  |  |  |  |  |  | | --- | --- | --- | --- | --- | --- | --- | --- | | **Overview** | **Package** | **Class** | **Use** | **Tree** | **Deprecated** | **Index** | **Help** | | |  |
| **PREV CLASS**   **NEXT CLASS** | **FRAMES**    **NO FRAMES**     **All Classes** |
| SUMMARY: NESTED | ENUM CONSTANTS | FIELD | METHOD | DETAIL: ENUM CONSTANTS | FIELD | METHOD |


---


## engine Enum CommandLineSimulation.Option

```
java.lang.Object
  java.lang.Enum<CommandLineSimulation.Option>
      engine.CommandLineSimulation.Option
```

**All Implemented Interfaces:**: java.io.Serializable, java.lang.Comparable<CommandLineSimulation.Option>

**Enclosing class:**: CommandLineSimulation

---

``` static enum CommandLineSimulation.Option extends java.lang.Enum<CommandLineSimulation.Option> ```

---

| **Enum Constant Summary** | |
| --- | --- |
| `config` |
| `dir` |
| `ee` |
| `em` |
| `graphic` |
| `help` |
| `lattice` |
| `log` |
| `mcs` |
| `mm` |
| `name` |
| `noauto` |
| `verbose` |


| **Method Summary** | |
| --- | --- |
| `static CommandLineSimulation.Option` | `valueOf(java.lang.String name)`             Returns the enum constant of this type with the specified name. |
| `static CommandLineSimulation.Option[]` | `values()`             Returns an array containing the constants of this enum type, in the order they are declared. |

| **Methods inherited from class java.lang.Enum** |
| --- |
| `clone, compareTo, equals, finalize, getDeclaringClass, hashCode, name, ordinal, toString, valueOf` |

| **Methods inherited from class java.lang.Object** |
| --- |
| `getClass, notify, notifyAll, wait, wait, wait` |

| **Enum Constant Detail** |
| --- |

### dir

```
public static final CommandLineSimulation.Option dir
```

---


### name

```
public static final CommandLineSimulation.Option name
```

---


### ee

```
public static final CommandLineSimulation.Option ee
```

---


### mm

```
public static final CommandLineSimulation.Option mm
```

---


### em

```
public static final CommandLineSimulation.Option em
```

---


### config

```
public static final CommandLineSimulation.Option config
```

---


### noauto

```
public static final CommandLineSimulation.Option noauto
```

---


### graphic

```
public static final CommandLineSimulation.Option graphic
```

---


### lattice

```
public static final CommandLineSimulation.Option lattice
```

---


### verbose

```
public static final CommandLineSimulation.Option verbose
```

---


### log

```
public static final CommandLineSimulation.Option log
```

---


### mcs

```
public static final CommandLineSimulation.Option mcs
```

---


### help

```
public static final CommandLineSimulation.Option help
```


| **Method Detail** |
| --- |

### values

```
public static CommandLineSimulation.Option[] values()
```

:   Returns an array containing the constants of this enum type, in
    the order they are declared. This method may be used to iterate
    over the constants as follows:

    ```
    for (CommandLineSimulation.Option c : CommandLineSimulation.Option.values())
        System.out.println(c);
    ```

    :   **Returns:**: an array containing the constants of this enum type, in the order they are declared

---


### valueOf

```
public static CommandLineSimulation.Option valueOf(java.lang.String name)
```

:   Returns the enum constant of this type with the specified name.
    The string must match *exactly* an identifier used to declare an
    enum constant in this type. (Extraneous whitespace characters are
    not permitted.)

    :   **Parameters:**: `name` - the name of the enum constant to be returned. **Returns:**: the enum constant with the specified name **Throws:**: `java.lang.IllegalArgumentException` - if this enum type has no constant with the specified name: `java.lang.NullPointerException` - if the argument is null


---


|  |  |  |  |  |  |  |  |  |  |  |
| --- | --- | --- | --- | --- | --- | --- | --- | --- | --- | --- |
| |  |  |  |  |  |  |  |  | | --- | --- | --- | --- | --- | --- | --- | --- | | **Overview** | **Package** | **Class** | **Use** | **Tree** | **Deprecated** | **Index** | **Help** | | |  |
| **PREV CLASS**   **NEXT CLASS** | **FRAMES**    **NO FRAMES**     **All Classes** |
| SUMMARY: NESTED | ENUM CONSTANTS | FIELD | METHOD | DETAIL: ENUM CONSTANTS | FIELD | METHOD |


---
