# Supplementary material for: Sorting at embryonic boundaries requires high heterotypic interfacial tension
Source: Nat Commun. 2017 Jul 31;8:157. doi: 10.1038/s41467-017-00146-x (PMC5537356; doi:10.1038/s41467-017-00146-x)
Supplement: Supplementary file 2 — Supplementary Software 1 [file 41467_2017_146_MOESM2_ESM.zip › PottsModel/SrcPottsModel/doc/engine/CSVStatistic.html]

CSVStatistic


JavaScript is disabled on your browser.


Skip navigation links


- Overview
- Package
- Class
- Use
- Tree
- Deprecated
- Index
- Help

- Prev Class
- Next Class

- Frames
- No Frames

- All Classes

- Summary:
- Nested |
- Field |
- Constr |
- Method

- Detail:
- Field |
- Constr |
- Method


engine

## Class CSVStatistic<LabelEnum extends java.lang.Enum<LabelEnum>>

- java.lang.Object
- - engine.Statistic
  - - engine.CSVStatistic<LabelEnum>

- Direct Known Subclasses:
  :   CellCoordinatesCSVStatistic, CellShapeCSVStatistic

  ---

    

  ```
  public abstract class CSVStatistic<LabelEnum extends java.lang.Enum<LabelEnum>>
  extends Statistic
  ```

- - ### Nested Class Summary

    - ### Nested classes/interfaces inherited from class engine.Statistic

      `Statistic.Utils`
  - ### Field Summary

    - ### Fields inherited from class engine.Statistic

      `DEFAULT_FREQUENCY`
  - ### Constructor Summary

    Constructors

    | Constructor and Description |
    | `CSVStatistic(PottsEngine engine, int frequency)` |
  - ### Method Summary

    All Methods Instance Methods Abstract Methods Concrete Methods

    | Modifier and Type | Method and Description |
    | `abstract LabelEnum[]` | `getColumnLabelEnums()` |
    | `java.lang.String[]` | `getColumnLabels()` |
    | `abstract java.lang.String` | `getCSVRow(PottsEngine paramPottsEngine, Cell paramCell)` |
    | `abstract java.lang.String` | `getFilenamePrefix()` |
    | `java.lang.String[]` | `getSeriesNames()` |
    | `java.lang.String` | `getYAxisLabel()` |
    | `boolean` | `isDisplayed()` |
    | `abstract boolean` | `isSpinAttemptsObserved()` |
    | `abstract boolean` | `isStateObservable(PottsEngine.State paramState)` |
    | `void` | `observe(PottsEngine.State engineState)` Observe values during MCS. |

    - ### Methods inherited from class engine.Statistic

      `addToManagerStatistics, attachPlotPanel, getAxis, getEngine, getFrequency, getLastValues, isAreaRendered, setRepeatFrequency, wrapUp`
    - ### Methods inherited from class java.lang.Object

      `equals, getClass, hashCode, notify, notifyAll, toString, wait, wait, wait`

- - ### Constructor Detail


    - #### CSVStatistic

      ```
      public CSVStatistic(PottsEngine engine,
                          int frequency)
      ```
  - ### Method Detail


    - #### getColumnLabelEnums

      ```
      public abstract LabelEnum[] getColumnLabelEnums()
      ```


    - #### getCSVRow

      ```
      public abstract java.lang.String getCSVRow(PottsEngine paramPottsEngine,
                                                 Cell paramCell)
      ```


    - #### getFilenamePrefix

      ```
      public abstract java.lang.String getFilenamePrefix()
      ```


    - #### isStateObservable

      ```
      public abstract boolean isStateObservable(PottsEngine.State paramState)
      ```


    - #### getColumnLabels

      ```
      public java.lang.String[] getColumnLabels()
      ```


    - #### observe

      ```
      public void observe(PottsEngine.State engineState)
      ```

      Description copied from class: `Statistic`

      Observe values during MCS.

      Specified by:
      :   `observe` in class `Statistic`


    - #### isDisplayed

      ```
      public boolean isDisplayed()
      ```

      Overrides:
      :   `isDisplayed` in class `Statistic`


    - #### isSpinAttemptsObserved

      ```
      public abstract boolean isSpinAttemptsObserved()
      ```

      Overrides:
      :   `isSpinAttemptsObserved` in class `Statistic`


    - #### getSeriesNames

      ```
      public java.lang.String[] getSeriesNames()
      ```

      Specified by:
      :   `getSeriesNames` in class `Statistic`


    - #### getYAxisLabel

      ```
      public java.lang.String getYAxisLabel()
      ```

      Overrides:
      :   `getYAxisLabel` in class `Statistic`


Skip navigation links


- Overview
- Package
- Class
- Use
- Tree
- Deprecated
- Index
- Help

- Prev Class
- Next Class

- Frames
- No Frames

- All Classes

- Summary:
- Nested |
- Field |
- Constr |
- Method

- Detail:
- Field |
- Constr |
- Method
