# Supplementary material for: Sorting at embryonic boundaries requires high heterotypic interfacial tension
Source: Nat Commun. 2017 Jul 31;8:157. doi: 10.1038/s41467-017-00146-x (PMC5537356; doi:10.1038/s41467-017-00146-x)
Supplement: Supplementary file 2 — Supplementary Software 1 [file 41467_2017_146_MOESM2_ESM.zip › PottsModel/SrcPottsModel/doc/engine/DispersionIndex.QuadratManager.html]

DispersionIndex.QuadratManager


---


|  |  |  |  |  |  |  |  |  |  |  |
| --- | --- | --- | --- | --- | --- | --- | --- | --- | --- | --- |
| |  |  |  |  |  |  |  |  | | --- | --- | --- | --- | --- | --- | --- | --- | | **Overview** | **Package** | **Class** | **Use** | **Tree** | **Deprecated** | **Index** | **Help** | | |  |
| **PREV CLASS**   **NEXT CLASS** | **FRAMES**    **NO FRAMES**     **All Classes** |
| SUMMARY: NESTED | FIELD | CONSTR | METHOD | DETAIL: FIELD | CONSTR | METHOD |


---


## engine Class DispersionIndex.QuadratManager

```
java.lang.Object
  engine.DispersionIndex.QuadratManager
```

**Enclosing class:**: DispersionIndex

---

``` private class DispersionIndex.QuadratManager extends java.lang.Object ```

---

| **Field Summary** | |
| --- | --- |
| `private  float[]` | `bins` |
| `private  Cell.CellType` | `cellType` |
| `int` | `n` |
| `private  int` | `numQuadratsInColumn` |
| `private  int` | `numQuadratsInRow` |
| `private  int` | `quadratSize` |


| **Constructor Summary** | |
| --- | --- |
| `DispersionIndex.QuadratManager(Cell.CellType c, int pixelsPerCell, int width, int height)` |


| **Method Summary** | |
| --- | --- |
| `private  int` | `getQuadratIndex(int x, int y)` |
| `float` | `mean()` |
| `private  void` | `reset()` |
| `void` | `scoreCell(Cell c)` |
| `float` | `var(float m)` |

| **Methods inherited from class java.lang.Object** |
| --- |
| `clone, equals, finalize, getClass, hashCode, notify, notifyAll, toString, wait, wait, wait` |

| **Field Detail** |
| --- |

### bins

```
private float[] bins
```

---


### cellType

```
private final Cell.CellType cellType
```

---


### quadratSize

```
private int quadratSize
```

---


### numQuadratsInRow

```
private int numQuadratsInRow
```

---


### numQuadratsInColumn

```
private int numQuadratsInColumn
```

---


### n

```
public int n
```


| **Constructor Detail** |
| --- |

### DispersionIndex.QuadratManager

```
public DispersionIndex.QuadratManager(Cell.CellType c,
                                      int pixelsPerCell,
                                      int width,
                                      int height)
```


| **Method Detail** |
| --- |

### reset

```
private void reset()
```

---


### getQuadratIndex

```
private int getQuadratIndex(int x,
                            int y)
```

---


### scoreCell

```
public void scoreCell(Cell c)
```

---


### mean

```
public float mean()
```

---


### var

```
public float var(float m)
```


---


|  |  |  |  |  |  |  |  |  |  |  |
| --- | --- | --- | --- | --- | --- | --- | --- | --- | --- | --- |
| |  |  |  |  |  |  |  |  | | --- | --- | --- | --- | --- | --- | --- | --- | | **Overview** | **Package** | **Class** | **Use** | **Tree** | **Deprecated** | **Index** | **Help** | | |  |
| **PREV CLASS**   **NEXT CLASS** | **FRAMES**    **NO FRAMES**     **All Classes** |
| SUMMARY: NESTED | FIELD | CONSTR | METHOD | DETAIL: FIELD | CONSTR | METHOD |


---
