# Supplementary material for: Sorting at embryonic boundaries requires high heterotypic interfacial tension
Source: Nat Commun. 2017 Jul 31;8:157. doi: 10.1038/s41467-017-00146-x (PMC5537356; doi:10.1038/s41467-017-00146-x)
Supplement: Supplementary file 2 — Supplementary Software 1 [file 41467_2017_146_MOESM2_ESM.zip › PottsModel/SrcPottsModel/doc/engine/package-frame.html]

engine


# engine

## Classes

- AreaEnergyStatistic
- AreaStatistic
- CellCoordinatesCSVStatistic
- CellEnergyStatistics
- CellShapeCSVStatistic
- CellStatistic
- CommandLineSimulation
- CSVStatistic
- DispersionIndex
- EnergyStatistic
- HBLStatistic
- HMDStatistic
- InteractionEnergyStatistic
- IsoperimetricQuotientStatistic
- PerimeterStatistic
- PottsEngine
- PottsLogger
- Simulation
- Statistic
- Statistic.Utils
- StatisticsManager
- TypeSpecificAreaStatistic
- TypeSpecificCellStatistic
- TypeSpecificNearestNeighborStatistic
- TypeSpecificNumNeighborsStatistic
- TypeSpecificPercentIsolatedCellStatistic
- TypeSpecificPerimeterStatistic
- TypeSpecificStatistic
- Utils
- Utils.EnergyTracker

## Enums

- CellShapeCSVLabel
- PottsEngine.State
