# Supplementary material for: Sorting at embryonic boundaries requires high heterotypic interfacial tension
Source: Nat Commun. 2017 Jul 31;8:157. doi: 10.1038/s41467-017-00146-x (PMC5537356; doi:10.1038/s41467-017-00146-x)
Supplement: Supplementary file 2 — Supplementary Software 1 [file 41467_2017_146_MOESM2_ESM.zip › PottsModel/SrcPottsModel/doc/engine/package-summary.html]

engine


JavaScript is disabled on your browser.


Skip navigation links


- Overview
- Package
- Class
- Use
- Tree
- Deprecated
- Index
- Help

- Prev Package
- Next Package

- Frames
- No Frames

- All Classes

# Package engine

- Class Summary

  | Class | Description |
  |  |  |
  | --- | --- |
  | AreaEnergyStatistic |  |
  | AreaStatistic |  |
  | CellCoordinatesCSVStatistic |  |
  | CellEnergyStatistics |  |
  | CellShapeCSVStatistic | Tracks cell shape statistics (area, perimeter, center of mass) in a separate csv file for \*\*EACH CELL\*\* Therefore, this statistic is memory intensive and only recommended for a very limited number of MCS. |
  | CellStatistic |  |
  | CommandLineSimulation |  |
  | CSVStatistic<LabelEnum extends java.lang.Enum<LabelEnum>> |  |
  | DispersionIndex | Dispersion index statistic measured for each cell type using 10x10 quadrats. |
  | EnergyStatistic |  |
  | HBLStatistic |  |
  | HMDStatistic | Homotypic Minimal Distance statistic. |
  | InteractionEnergyStatistic |  |
  | IsoperimetricQuotientStatistic |  |
  | PerimeterStatistic |  |
  | PottsEngine | Class describing core loop functionality of the PottsModel simulation. |
  | PottsLogger | Potts Automaton Logger. |
  | Simulation |  |
  | Statistic |  |
  | Statistic.Utils |  |
  | StatisticsManager | Manages statistics by initailizing and evaluating the Statistics according to the frequency defined by the user in the simulation settings file. |
  | TypeSpecificAreaStatistic |  |
  | TypeSpecificCellStatistic |  |
  | TypeSpecificNearestNeighborStatistic |  |
  | TypeSpecificNumNeighborsStatistic |  |
  | TypeSpecificPercentIsolatedCellStatistic |  |
  | TypeSpecificPerimeterStatistic |  |
  | TypeSpecificStatistic |  |
  | Utils |  |
  | Utils.EnergyTracker |  |
- Enum Summary

  | Enum | Description |
  |  |  |
  | --- | --- |
  | CellShapeCSVLabel |  |
  | PottsEngine.State | Enum class representing the different states of the potts engine that can be passed as a notification message to the different class observers. |

Skip navigation links


- Overview
- Package
- Class
- Use
- Tree
- Deprecated
- Index
- Help

- Prev Package
- Next Package

- Frames
- No Frames

- All Classes
