# Supplementary material for: Sorting at embryonic boundaries requires high heterotypic interfacial tension
Source: Nat Commun. 2017 Jul 31;8:157. doi: 10.1038/s41467-017-00146-x (PMC5537356; doi:10.1038/s41467-017-00146-x)
Supplement: Supplementary file 2 — Supplementary Software 1 [file 41467_2017_146_MOESM2_ESM.zip › PottsModel/SrcPottsModel/doc/engine/package-tree.html]

engine Class Hierarchy


JavaScript is disabled on your browser.


Skip navigation links


- Overview
- Package
- Class
- Use
- Tree
- Deprecated
- Index
- Help

- Prev
- Next

- Frames
- No Frames

- All Classes

# Hierarchy For Package engine

Package Hierarchies:

- All Packages

## Class Hierarchy

- java.lang.Object
  - mvc.AObservable (implements mvc.IObservable)
    - engine.PottsEngine (implements model.KnowsConstants)
    - engine.StatisticsManager (implements mvc.Observer)
  - engine.CommandLineSimulation
  - engine.PottsEngine.StateVariables
  - engine.PottsLogger (implements mvc.Observer)
  - engine.Simulation
  - engine.Statistic
    - engine.CellEnergyStatistics
    - engine.CellStatistic
      - engine.AreaEnergyStatistic
      - engine.AreaStatistic
      - engine.InteractionEnergyStatistic
      - engine.PerimeterStatistic
    - engine.CSVStatistic<LabelEnum>
      - engine.CellCoordinatesCSVStatistic
      - engine.CellShapeCSVStatistic
    - engine.DispersionIndex
    - engine.EnergyStatistic
    - engine.HBLStatistic
    - engine.TypeSpecificCellStatistic
      - engine.HMDStatistic
      - engine.IsoperimetricQuotientStatistic
      - engine.TypeSpecificAreaStatistic
      - engine.TypeSpecificNearestNeighborStatistic
      - engine.TypeSpecificNumNeighborsStatistic
      - engine.TypeSpecificPerimeterStatistic
    - engine.TypeSpecificStatistic
      - engine.TypeSpecificPercentIsolatedCellStatistic
  - engine.Statistic.Utils
  - engine.Utils
  - engine.Utils.EnergyTracker

## Enum Hierarchy

- java.lang.Object
  - java.lang.Enum<E> (implements java.lang.Comparable<T>, java.io.Serializable)
    - engine.PottsEngine.State
    - engine.CellShapeCSVLabel

Skip navigation links


- Overview
- Package
- Class
- Use
- Tree
- Deprecated
- Index
- Help

- Prev
- Next

- Frames
- No Frames

- All Classes
