# Supplementary material for: Sorting at embryonic boundaries requires high heterotypic interfacial tension
Source: Nat Commun. 2017 Jul 31;8:157. doi: 10.1038/s41467-017-00146-x (PMC5537356; doi:10.1038/s41467-017-00146-x)
Supplement: Supplementary file 2 — Supplementary Software 1 [file 41467_2017_146_MOESM2_ESM.zip › PottsModel/SrcPottsModel/doc/engine/package-use.html]

Uses of Package engine


JavaScript is disabled on your browser.


Skip navigation links


- Overview
- Package
- Class
- Use
- Tree
- Deprecated
- Index
- Help

- Prev
- Next

- Frames
- No Frames

- All Classes

# Uses of Package engine

- Packages that use engine

  | Package | Description |
  |  |  |
  | --- | --- |
  | engine |  |
  | gui |  |
  | model |  |
  | simulations |  |
- Classes in engine used by engine

  | Class and Description |
  |  |
  | --- |
  | CellShapeCSVLabel |
  | CellStatistic |
  | CSVStatistic |
  | PottsEngine Class describing core loop functionality of the PottsModel simulation. |
  | PottsEngine.State Enum class representing the different states of the potts engine that can be passed as a notification message to the different class observers. |
  | PottsEngine.StateVariables Initializes state variables (if they are objects, they are set to null) |
  | Simulation |
  | Statistic |
  | TypeSpecificCellStatistic |
  | TypeSpecificStatistic |
  | Utils.EnergyTracker |
- Classes in engine used by gui

  | Class and Description |
  |  |
  | --- |
  | PottsEngine Class describing core loop functionality of the PottsModel simulation. |
  | Statistic |
  | StatisticsManager Manages statistics by initailizing and evaluating the Statistics according to the frequency defined by the user in the simulation settings file. |
- Classes in engine used by model

  | Class and Description |
  |  |
  | --- |
  | PottsEngine.StateVariables Initializes state variables (if they are objects, they are set to null) |
  | Utils.EnergyTracker |
- Classes in engine used by simulations

  | Class and Description |
  |  |
  | --- |
  | Simulation |

Skip navigation links


- Overview
- Package
- Class
- Use
- Tree
- Deprecated
- Index
- Help

- Prev
- Next

- Frames
- No Frames

- All Classes
