# Supplementary material for: Sorting at embryonic boundaries requires high heterotypic interfacial tension
Source: Nat Commun. 2017 Jul 31;8:157. doi: 10.1038/s41467-017-00146-x (PMC5537356; doi:10.1038/s41467-017-00146-x)
Supplement: Supplementary file 2 — Supplementary Software 1 [file 41467_2017_146_MOESM2_ESM.zip › PottsModel/SrcPottsModel/doc/engine/PottsEngine.html]

PottsEngine


JavaScript is disabled on your browser.


Skip navigation links


- Overview
- Package
- Class
- Use
- Tree
- Deprecated
- Index
- Help

- Prev Class
- Next Class

- Frames
- No Frames

- All Classes

- Summary:
- Nested |
- Field |
- Constr |
- Method

- Detail:
- Field |
- Constr |
- Method


engine

## Class PottsEngine

- java.lang.Object
- - mvc.AObservable
  - - engine.PottsEngine

- All Implemented Interfaces:
  :   KnowsConstants, IObservable

  ---

    

  ```
  public class PottsEngine
  extends AObservable
  implements KnowsConstants
  ```

  Class describing core loop functionality of the PottsModel simulation. Based
  on the passed Constants object, this object will initialize the Lattice
  (which in turn initializes Pixel and Cell objects (see respective classes for
  more details), the EnergyManager which will compute energies given a certain
  model (e.g. classical, realistic etc.), the graphical interface if needed as
  well as various bookkeeping objects. Once that is done, a simulation can be
  ran using runSimulation().
  The most important methods in this class can be found under "CORE METHODS".
  Each method is commented in detail but here is an overview:
  - tick():
  controls what happens at each "tick" of the clock like attempting spin copies
  - trySpinCopy(Pixel s, Pixel t):
  Given a source and a target pixel, decide whether or not to execute this
  spin copy based on this model's energy function.
  - copyCriterion(double energyBefore, double energyAfter):
  If the difference in energy is negative, then return true.
  Otherwise, return false with a Boltzmann probability that increases with
  temperature and with Delta(Energy)
  - runSimulation(): runs simulation given the current Constants object
  Note: The main constructor for this class takes an array of Constants objects
  so that simulations can be ran consecutively.

  Author:
  :   eleyine

  See Also:
  :   `Lattice`,
      `Pixel`,
      `Cell`

- - ### Nested Class Summary

    Nested Classes

    | Modifier and Type | Class and Description |
    | `static class` | `PottsEngine.State` Enum class representing the different states of the potts engine that can be passed as a notification message to the different class observers. |
    | `class` | `PottsEngine.StateVariables` Initializes state variables (if they are objects, they are set to null) |
  - ### Field Summary

    Fields

    | Modifier and Type | Field and Description |
    | `java.util.concurrent.atomic.AtomicBoolean` | `aAutomaticMode` |
    | `java.util.concurrent.atomic.AtomicBoolean` | `aDoMCS` |
    | `java.util.concurrent.atomic.AtomicBoolean` | `aDone` |
    | `java.util.concurrent.atomic.AtomicBoolean` | `aDoSwap` |
    | `boolean` | `aGraphicalOutputEnabled` |
    | `Lattice` | `aLattice` |
    | `PottsEngine.StateVariables` | `aStateVariables` |
    | `java.lang.Thread` | `aThread` |
    | `static java.lang.String` | `cNotificationSource` |
  - ### Constructor Summary

    Constructors

    | Constructor and Description |
    | `PottsEngine(Constants pConstants)` |
    | `PottsEngine(Constants[] pConstants)` |
    | `PottsEngine(Constants pConstants, Lattice pLattice)` |
    | `PottsEngine(Simulation pSimulation)` |
  - ### Method Summary

    All Methods Static Methods Instance Methods Concrete Methods

    | Modifier and Type | Method and Description |
    | `static boolean` | `copyCriterion(double pDeltaEnergy, double pTemperature)` Returns whether the copy from source to target pixel should be done. |
    | `Constants` | `getConstants()` |
    | `int` | `getCurrentSimulationNumber()` |
    | `java.util.List<EnergyFunction.Energy>` | `getEnergyTypes()` |
    | `int` | `getMCSCount()` |
    | `int` | `getNumberSimulations()` |
    | `Lattice.PixelIterator` | `getPixelIterator()` |
    | `long` | `getSimulationStartTime()` |
    | `int` | `getSpinCopyAttemptCount()` |
    | `void` | `runSimulation()` Run simulation for given Constants once the PottsEngine has been initialized. |
    | `void` | `setRecorded(boolean pIsRecorded)` |

    - ### Methods inherited from class mvc.AObservable

      `addObserver, getNotificationSequenceNumber, getNotificationSource, notifyObservers, removeAllObservers, removeObserver`
    - ### Methods inherited from class java.lang.Object

      `equals, getClass, hashCode, notify, notifyAll, toString, wait, wait, wait`

- - ### Field Detail


    - #### cNotificationSource

      ```
      public static final java.lang.String cNotificationSource
      ```

      See Also:
      :   Constant Field Values


    - #### aLattice

      ```
      public Lattice aLattice
      ```


    - #### aStateVariables

      ```
      public PottsEngine.StateVariables aStateVariables
      ```


    - #### aGraphicalOutputEnabled

      ```
      public final boolean aGraphicalOutputEnabled
      ```


    - #### aThread

      ```
      public java.lang.Thread aThread
      ```


    - #### aDone

      ```
      public java.util.concurrent.atomic.AtomicBoolean aDone
      ```


    - #### aAutomaticMode

      ```
      public java.util.concurrent.atomic.AtomicBoolean aAutomaticMode
      ```


    - #### aDoSwap

      ```
      public java.util.concurrent.atomic.AtomicBoolean aDoSwap
      ```


    - #### aDoMCS

      ```
      public java.util.concurrent.atomic.AtomicBoolean aDoMCS
      ```
  - ### Constructor Detail


    - #### PottsEngine

      ```
      public PottsEngine(Constants[] pConstants)
      ```


    - #### PottsEngine

      ```
      public PottsEngine(Constants pConstants)
      ```


    - #### PottsEngine

      ```
      public PottsEngine(Simulation pSimulation)
      ```


    - #### PottsEngine

      ```
      public PottsEngine(Constants pConstants,
                         Lattice pLattice)
      ```
  - ### Method Detail


    - #### copyCriterion

      ```
      public static boolean copyCriterion(double pDeltaEnergy,
                                          double pTemperature)
      ```

      Returns whether the copy from source to target pixel should be done. If
      Delta(Energy)< 0, then return true. Otherwise, return false with a
      Boltzmann probability that increases with temperature and with
      Delta(Energy).

      Parameters:
      :   `pDeltaEnergy` - the local energy after the pixels are swapped - the local
          energy before the pixels are swapped
      :   `pTemperature` - the original source pixel's temperature

      Returns:
      :   true if pixels must be swapped


    - #### runSimulation

      ```
      public void runSimulation()
      ```

      Run simulation for given Constants once the PottsEngine has been
      initialized.


    - #### getMCSCount

      ```
      public int getMCSCount()
      ```


    - #### getSpinCopyAttemptCount

      ```
      public int getSpinCopyAttemptCount()
      ```


    - #### getPixelIterator

      ```
      public Lattice.PixelIterator getPixelIterator()
      ```


    - #### getEnergyTypes

      ```
      public java.util.List<EnergyFunction.Energy> getEnergyTypes()
      ```


    - #### getConstants

      ```
      public Constants getConstants()
      ```

      Specified by:
      :   `getConstants` in interface `KnowsConstants`


    - #### getSimulationStartTime

      ```
      public long getSimulationStartTime()
      ```


    - #### getNumberSimulations

      ```
      public int getNumberSimulations()
      ```


    - #### getCurrentSimulationNumber

      ```
      public int getCurrentSimulationNumber()
      ```


    - #### setRecorded

      ```
      public void setRecorded(boolean pIsRecorded)
      ```


Skip navigation links


- Overview
- Package
- Class
- Use
- Tree
- Deprecated
- Index
- Help

- Prev Class
- Next Class

- Frames
- No Frames

- All Classes

- Summary:
- Nested |
- Field |
- Constr |
- Method

- Detail:
- Field |
- Constr |
- Method
