# Supplementary material for: Sorting at embryonic boundaries requires high heterotypic interfacial tension
Source: Nat Commun. 2017 Jul 31;8:157. doi: 10.1038/s41467-017-00146-x (PMC5537356; doi:10.1038/s41467-017-00146-x)
Supplement: Supplementary file 2 — Supplementary Software 1 [file 41467_2017_146_MOESM2_ESM.zip › PottsModel/SrcPottsModel/doc/engine/PottsEngine.StateVariables.html]

PottsEngine.StateVariables


JavaScript is disabled on your browser.


Skip navigation links


- Overview
- Package
- Class
- Use
- Tree
- Deprecated
- Index
- Help

- Prev Class
- Next Class

- Frames
- No Frames

- All Classes

- Summary:
- Nested |
- Field |
- Constr |
- Method

- Detail:
- Field |
- Constr |
- Method


engine

## Class PottsEngine.StateVariables

- java.lang.Object
- - engine.PottsEngine.StateVariables

- Enclosing class:
  :   PottsEngine

  ---

    

  ```
  public class PottsEngine.StateVariables
  extends java.lang.Object
  ```

  Initializes state variables (if they are objects, they are set to null)

  Author:
  :   eleyine

- - ### Field Summary

    Fields

    | Modifier and Type | Field and Description |
    | `Utils.EnergyTracker` | `aDeltaEnergyTracker` |
    | `Pixel` | `aPixelSwapSource` |
    | `Pixel` | `aPixelSwapTarget` |
    | `Pixel[]` | `aToRedraw` |
  - ### Constructor Summary

    Constructors

    | Constructor and Description |
    | `StateVariables()` |
  - ### Method Summary

    - ### Methods inherited from class java.lang.Object

      `equals, getClass, hashCode, notify, notifyAll, toString, wait, wait, wait`

- - ### Field Detail


    - #### aPixelSwapSource

      ```
      public Pixel aPixelSwapSource
      ```


    - #### aPixelSwapTarget

      ```
      public Pixel aPixelSwapTarget
      ```


    - #### aDeltaEnergyTracker

      ```
      public Utils.EnergyTracker aDeltaEnergyTracker
      ```


    - #### aToRedraw

      ```
      public Pixel[] aToRedraw
      ```
  - ### Constructor Detail


    - #### StateVariables

      ```
      public StateVariables()
      ```


Skip navigation links


- Overview
- Package
- Class
- Use
- Tree
- Deprecated
- Index
- Help

- Prev Class
- Next Class

- Frames
- No Frames

- All Classes

- Summary:
- Nested |
- Field |
- Constr |
- Method

- Detail:
- Field |
- Constr |
- Method
