# Supplementary material for: Sorting at embryonic boundaries requires high heterotypic interfacial tension
Source: Nat Commun. 2017 Jul 31;8:157. doi: 10.1038/s41467-017-00146-x (PMC5537356; doi:10.1038/s41467-017-00146-x)
Supplement: Supplementary file 2 — Supplementary Software 1 [file 41467_2017_146_MOESM2_ESM.zip › PottsModel/SrcPottsModel/doc/engine/PottsLogger.html]

PottsLogger


JavaScript is disabled on your browser.


Skip navigation links


- Overview
- Package
- Class
- Use
- Tree
- Deprecated
- Index
- Help

- Prev Class
- Next Class

- Frames
- No Frames

- All Classes

- Summary:
- Nested |
- Field |
- Constr |
- Method

- Detail:
- Field |
- Constr |
- Method


engine

## Class PottsLogger

- java.lang.Object
- - engine.PottsLogger

- All Implemented Interfaces:
  :   Observer

  ---

    

  ```
  public class PottsLogger
  extends java.lang.Object
  implements Observer
  ```

  Potts Automaton Logger.
  Additional logging settings can be set in config/log4j2.xml

  Author:
  :   Eleyine

- - ### Constructor Summary

    Constructors

    | Constructor and Description |
    | `PottsLogger()` |
  - ### Method Summary

    All Methods Instance Methods Concrete Methods

    | Modifier and Type | Method and Description |
    | `void` | `update(javax.management.Notification pNotification)` Determines what an observer should do upon notification that the observed object has changed. |

    - ### Methods inherited from class java.lang.Object

      `equals, getClass, hashCode, notify, notifyAll, toString, wait, wait, wait`

- - ### Constructor Detail


    - #### PottsLogger

      ```
      public PottsLogger()
      ```
  - ### Method Detail


    - #### update

      ```
      public void update(javax.management.Notification pNotification)
      ```

      Description copied from interface: `Observer`

      Determines what an observer should do upon notification that the observed object has changed.

      Specified by:
      :   `update` in interface `Observer`

      Parameters:
      :   `pNotification` - : Notification passed by the object being observed.


Skip navigation links


- Overview
- Package
- Class
- Use
- Tree
- Deprecated
- Index
- Help

- Prev Class
- Next Class

- Frames
- No Frames

- All Classes

- Summary:
- Nested |
- Field |
- Constr |
- Method

- Detail:
- Field |
- Constr |
- Method
