# Supplementary material for: Sorting at embryonic boundaries requires high heterotypic interfacial tension
Source: Nat Commun. 2017 Jul 31;8:157. doi: 10.1038/s41467-017-00146-x (PMC5537356; doi:10.1038/s41467-017-00146-x)
Supplement: Supplementary file 2 — Supplementary Software 1 [file 41467_2017_146_MOESM2_ESM.zip › PottsModel/SrcPottsModel/doc/engine/Simulation.html]

Simulation


JavaScript is disabled on your browser.


Skip navigation links


- Overview
- Package
- Class
- Use
- Tree
- Deprecated
- Index
- Help

- Prev Class
- Next Class

- Frames
- No Frames

- All Classes

- Summary:
- Nested |
- Field |
- Constr |
- Method

- Detail:
- Field |
- Constr |
- Method


engine

## Class Simulation

- java.lang.Object
- - engine.Simulation

- Direct Known Subclasses:
  :   BoundarySimulation, DefaultSimulation, DITHSimulation, EndoSimulation, FourByFourSimulation, GenerateRandomLattices, GranerGlazierSimulation, NegativeControl, SingleCell

  ---

    

  ```
  public abstract class Simulation
  extends java.lang.Object
  ```

- - ### Constructor Summary

    Constructors

    | Constructor and Description |
    | `Simulation()` |
  - ### Method Summary

    All Methods Instance Methods Abstract Methods Concrete Methods

    | Modifier and Type | Method and Description |
    | `abstract Constants[]` | `getConstants()` |
    | `void` | `run()` |

    - ### Methods inherited from class java.lang.Object

      `equals, getClass, hashCode, notify, notifyAll, toString, wait, wait, wait`

- - ### Constructor Detail


    - #### Simulation

      ```
      public Simulation()
      ```
  - ### Method Detail


    - #### run

      ```
      public void run()
      ```


    - #### getConstants

      ```
      public abstract Constants[] getConstants()
      ```


Skip navigation links


- Overview
- Package
- Class
- Use
- Tree
- Deprecated
- Index
- Help

- Prev Class
- Next Class

- Frames
- No Frames

- All Classes

- Summary:
- Nested |
- Field |
- Constr |
- Method

- Detail:
- Field |
- Constr |
- Method
