# Supplementary material for: Sorting at embryonic boundaries requires high heterotypic interfacial tension
Source: Nat Commun. 2017 Jul 31;8:157. doi: 10.1038/s41467-017-00146-x (PMC5537356; doi:10.1038/s41467-017-00146-x)
Supplement: Supplementary file 2 — Supplementary Software 1 [file 41467_2017_146_MOESM2_ESM.zip › PottsModel/SrcPottsModel/doc/engine/Statistic.html]

Statistic


JavaScript is disabled on your browser.


Skip navigation links


- Overview
- Package
- Class
- Use
- Tree
- Deprecated
- Index
- Help

- Prev Class
- Next Class

- Frames
- No Frames

- All Classes

- Summary:
- Nested |
- Field |
- Constr |
- Method

- Detail:
- Field |
- Constr |
- Method


engine

## Class Statistic

- java.lang.Object
- - engine.Statistic

- Direct Known Subclasses:
  :   CellEnergyStatistics, CellStatistic, CSVStatistic, DispersionIndex, EnergyStatistic, HBLStatistic, TypeSpecificCellStatistic, TypeSpecificStatistic

  ---

    

  ```
  public abstract class Statistic
  extends java.lang.Object
  ```

- - ### Nested Class Summary

    Nested Classes

    | Modifier and Type | Class and Description |
    | `static class` | `Statistic.Utils` |
  - ### Field Summary

    Fields

    | Modifier and Type | Field and Description |
    | `static int` | `DEFAULT_FREQUENCY` |
  - ### Constructor Summary

    Constructors

    | Constructor and Description |
    | `Statistic(PottsEngine engine, java.lang.String yAxisLabel)` |
    | `Statistic(PottsEngine engine, java.lang.String yAxisLabel, int frequency)` |
  - ### Method Summary

    All Methods Instance Methods Abstract Methods Concrete Methods

    | Modifier and Type | Method and Description |
    | `void` | `addToManagerStatistics(java.util.List<Statistic> statsList)` |
    | `void` | `attachPlotPanel(PlotPanel p)` |
    | `org.jfree.chart.axis.NumberAxis` | `getAxis()` |
    | `PottsEngine` | `getEngine()` |
    | `int` | `getFrequency()` |
    | `java.util.LinkedList<java.lang.Float>` | `getLastValues()` |
    | `abstract java.lang.String[]` | `getSeriesNames()` |
    | `java.lang.String` | `getYAxisLabel()` |
    | `boolean` | `isAreaRendered()` |
    | `boolean` | `isDisplayed()` |
    | `boolean` | `isSpinAttemptsObserved()` |
    | `abstract void` | `observe(PottsEngine.State paramState)` Observe values during MCS. |
    | `void` | `setRepeatFrequency(int newFrequency)` |
    | `void` | `wrapUp(int mcs)` Call at the end of a MCS |

    - ### Methods inherited from class java.lang.Object

      `equals, getClass, hashCode, notify, notifyAll, toString, wait, wait, wait`

- - ### Field Detail


    - #### DEFAULT\_FREQUENCY

      ```
      public static final int DEFAULT_FREQUENCY
      ```

      See Also:
      :   Constant Field Values
  - ### Constructor Detail


    - #### Statistic

      ```
      public Statistic(PottsEngine engine,
                       java.lang.String yAxisLabel,
                       int frequency)
      ```


    - #### Statistic

      ```
      public Statistic(PottsEngine engine,
                       java.lang.String yAxisLabel)
      ```
  - ### Method Detail


    - #### getYAxisLabel

      ```
      public java.lang.String getYAxisLabel()
      ```


    - #### getEngine

      ```
      public PottsEngine getEngine()
      ```


    - #### attachPlotPanel

      ```
      public void attachPlotPanel(PlotPanel p)
      ```


    - #### getSeriesNames

      ```
      public abstract java.lang.String[] getSeriesNames()
      ```


    - #### wrapUp

      ```
      public void wrapUp(int mcs)
      ```

      Call at the end of a MCS


    - #### observe

      ```
      public abstract void observe(PottsEngine.State paramState)
      ```

      Observe values during MCS.

      Parameters:
      :   `value` -


    - #### isSpinAttemptsObserved

      ```
      public boolean isSpinAttemptsObserved()
      ```


    - #### isAreaRendered

      ```
      public boolean isAreaRendered()
      ```


    - #### isDisplayed

      ```
      public boolean isDisplayed()
      ```


    - #### getAxis

      ```
      public org.jfree.chart.axis.NumberAxis getAxis()
      ```


    - #### addToManagerStatistics

      ```
      public void addToManagerStatistics(java.util.List<Statistic> statsList)
      ```


    - #### getLastValues

      ```
      public java.util.LinkedList<java.lang.Float> getLastValues()
      ```


    - #### setRepeatFrequency

      ```
      public void setRepeatFrequency(int newFrequency)
      ```


    - #### getFrequency

      ```
      public int getFrequency()
      ```


Skip navigation links


- Overview
- Package
- Class
- Use
- Tree
- Deprecated
- Index
- Help

- Prev Class
- Next Class

- Frames
- No Frames

- All Classes

- Summary:
- Nested |
- Field |
- Constr |
- Method

- Detail:
- Field |
- Constr |
- Method
