# Supplementary material for: Sorting at embryonic boundaries requires high heterotypic interfacial tension
Source: Nat Commun. 2017 Jul 31;8:157. doi: 10.1038/s41467-017-00146-x (PMC5537356; doi:10.1038/s41467-017-00146-x)
Supplement: Supplementary file 2 — Supplementary Software 1 [file 41467_2017_146_MOESM2_ESM.zip › PottsModel/SrcPottsModel/doc/engine/Statistic.Utils.html]

Statistic.Utils


JavaScript is disabled on your browser.


Skip navigation links


- Overview
- Package
- Class
- Use
- Tree
- Deprecated
- Index
- Help

- Prev Class
- Next Class

- Frames
- No Frames

- All Classes

- Summary:
- Nested |
- Field |
- Constr |
- Method

- Detail:
- Field |
- Constr |
- Method


engine

## Class Statistic.Utils

- java.lang.Object
- - engine.Statistic.Utils

- Enclosing class:
  :   Statistic

  ---

    

  ```
  public static class Statistic.Utils
  extends java.lang.Object
  ```

- - ### Constructor Summary

    Constructors

    | Constructor and Description |
    | `Utils()` |
  - ### Method Summary

    All Methods Static Methods Concrete Methods

    | Modifier and Type | Method and Description |
    | `static float` | `mean(float[] numbers)` |
    | `static double` | `mean(java.util.List<java.lang.Double> numbers)` |
    | `static double` | `median(java.util.LinkedList<java.lang.Double> lValues)` |
    | `static float` | `std(float[] numbers)` |
    | `static double` | `std(java.util.List<java.lang.Double> numbers)` |

    - ### Methods inherited from class java.lang.Object

      `equals, getClass, hashCode, notify, notifyAll, toString, wait, wait, wait`

- - ### Constructor Detail


    - #### Utils

      ```
      public Utils()
      ```
  - ### Method Detail


    - #### mean

      ```
      public static double mean(java.util.List<java.lang.Double> numbers)
      ```


    - #### std

      ```
      public static double std(java.util.List<java.lang.Double> numbers)
      ```


    - #### std

      ```
      public static float std(float[] numbers)
      ```


    - #### mean

      ```
      public static float mean(float[] numbers)
      ```


    - #### median

      ```
      public static double median(java.util.LinkedList<java.lang.Double> lValues)
      ```


Skip navigation links


- Overview
- Package
- Class
- Use
- Tree
- Deprecated
- Index
- Help

- Prev Class
- Next Class

- Frames
- No Frames

- All Classes

- Summary:
- Nested |
- Field |
- Constr |
- Method

- Detail:
- Field |
- Constr |
- Method
