# Supplementary material for: Sorting at embryonic boundaries requires high heterotypic interfacial tension
Source: Nat Commun. 2017 Jul 31;8:157. doi: 10.1038/s41467-017-00146-x (PMC5537356; doi:10.1038/s41467-017-00146-x)
Supplement: Supplementary file 2 — Supplementary Software 1 [file 41467_2017_146_MOESM2_ESM.zip › PottsModel/SrcPottsModel/doc/engine/StatisticsManager.html]

StatisticsManager


JavaScript is disabled on your browser.


Skip navigation links


- Overview
- Package
- Class
- Use
- Tree
- Deprecated
- Index
- Help

- Prev Class
- Next Class

- Frames
- No Frames

- All Classes

- Summary:
- Nested |
- Field |
- Constr |
- Method

- Detail:
- Field |
- Constr |
- Method


engine

## Class StatisticsManager

- java.lang.Object
- - mvc.AObservable
  - - engine.StatisticsManager

- All Implemented Interfaces:
  :   IObservable, Observer

  ---

    

  ```
  public class StatisticsManager
  extends AObservable
  implements Observer
  ```

  Manages statistics by initailizing and evaluating the Statistics according to the frequency
  defined by the user in the simulation settings file.

  Author:
  :   eleyine

- - ### Field Summary

    Fields

    | Modifier and Type | Field and Description |
    | `static java.lang.String` | `cNotificationSource` |
  - ### Constructor Summary

    Constructors

    | Constructor and Description |
    | `StatisticsManager(PottsEngine e)` |
  - ### Method Summary

    All Methods Instance Methods Concrete Methods

    | Modifier and Type | Method and Description |
    | `java.lang.String` | `getCSVHeader()` |
    | `javax.swing.JPanel` | `getPanel()` |
    | `boolean` | `isDeltaEnergyTracked()` |
    | `boolean` | `isSpinAttemptsObserved()` |
    | `void` | `setTracker(Utils.EnergyTracker t)` |
    | `void` | `update(javax.management.Notification pNotification)` Determines what an observer should do upon notification that the observed object has changed. |

    - ### Methods inherited from class mvc.AObservable

      `addObserver, getNotificationSequenceNumber, getNotificationSource, notifyObservers, removeAllObservers, removeObserver`
    - ### Methods inherited from class java.lang.Object

      `equals, getClass, hashCode, notify, notifyAll, toString, wait, wait, wait`

- - ### Field Detail


    - #### cNotificationSource

      ```
      public static java.lang.String cNotificationSource
      ```
  - ### Constructor Detail


    - #### StatisticsManager

      ```
      public StatisticsManager(PottsEngine e)
      ```
  - ### Method Detail


    - #### setTracker

      ```
      public void setTracker(Utils.EnergyTracker t)
      ```


    - #### isDeltaEnergyTracked

      ```
      public boolean isDeltaEnergyTracked()
      ```


    - #### isSpinAttemptsObserved

      ```
      public boolean isSpinAttemptsObserved()
      ```


    - #### getPanel

      ```
      public javax.swing.JPanel getPanel()
      ```


    - #### update

      ```
      public void update(javax.management.Notification pNotification)
      ```

      Description copied from interface: `Observer`

      Determines what an observer should do upon notification that the observed object has changed.

      Specified by:
      :   `update` in interface `Observer`

      Parameters:
      :   `pNotification` - : Notification passed by the object being observed.


    - #### getCSVHeader

      ```
      public java.lang.String getCSVHeader()
      ```


Skip navigation links


- Overview
- Package
- Class
- Use
- Tree
- Deprecated
- Index
- Help

- Prev Class
- Next Class

- Frames
- No Frames

- All Classes

- Summary:
- Nested |
- Field |
- Constr |
- Method

- Detail:
- Field |
- Constr |
- Method
