# Supplementary material for: Sorting at embryonic boundaries requires high heterotypic interfacial tension
Source: Nat Commun. 2017 Jul 31;8:157. doi: 10.1038/s41467-017-00146-x (PMC5537356; doi:10.1038/s41467-017-00146-x)
Supplement: Supplementary file 2 — Supplementary Software 1 [file 41467_2017_146_MOESM2_ESM.zip › PottsModel/SrcPottsModel/doc/engine/TypeSpecificNearestNeighborStatistic.html]

TypeSpecificNearestNeighborStatistic


JavaScript is disabled on your browser.


Skip navigation links


- Overview
- Package
- Class
- Use
- Tree
- Deprecated
- Index
- Help

- Prev Class
- Next Class

- Frames
- No Frames

- All Classes

- Summary:
- Nested |
- Field |
- Constr |
- Method

- Detail:
- Field |
- Constr |
- Method


engine

## Class TypeSpecificNearestNeighborStatistic

- java.lang.Object
- - engine.Statistic
  - - engine.TypeSpecificCellStatistic
    - - engine.TypeSpecificNearestNeighborStatistic

- ---

    

  ```
  public class TypeSpecificNearestNeighborStatistic
  extends TypeSpecificCellStatistic
  ```

- - ### Nested Class Summary

    - ### Nested classes/interfaces inherited from class engine.Statistic

      `Statistic.Utils`
  - ### Field Summary

    Fields

    | Modifier and Type | Field and Description |
    | `static java.lang.String` | `cMetricName` |

    - ### Fields inherited from class engine.Statistic

      `DEFAULT_FREQUENCY`
  - ### Constructor Summary

    Constructors

    | Constructor and Description |
    | `TypeSpecificNearestNeighborStatistic(PottsEngine pEngine, Cell.CellType[] pCellTypes, int pFrequency)` |
    | `TypeSpecificNearestNeighborStatistic(PottsEngine pEngine, Cell.CellType[] pCellTypes, int pFrequency, int pNumNeighbors)` |
  - ### Method Summary

    All Methods Instance Methods Concrete Methods

    | Modifier and Type | Method and Description |
    | `java.lang.String[]` | `getSeriesNames()` |
    | `void` | `observe(PottsEngine.State engineState)` Observe values during MCS. |

    - ### Methods inherited from class engine.Statistic

      `addToManagerStatistics, attachPlotPanel, getAxis, getEngine, getFrequency, getLastValues, getYAxisLabel, isAreaRendered, isDisplayed, isSpinAttemptsObserved, setRepeatFrequency, wrapUp`
    - ### Methods inherited from class java.lang.Object

      `equals, getClass, hashCode, notify, notifyAll, toString, wait, wait, wait`

- - ### Field Detail


    - #### cMetricName

      ```
      public static java.lang.String cMetricName
      ```
  - ### Constructor Detail


    - #### TypeSpecificNearestNeighborStatistic

      ```
      public TypeSpecificNearestNeighborStatistic(PottsEngine pEngine,
                                                  Cell.CellType[] pCellTypes,
                                                  int pFrequency,
                                                  int pNumNeighbors)
      ```


    - #### TypeSpecificNearestNeighborStatistic

      ```
      public TypeSpecificNearestNeighborStatistic(PottsEngine pEngine,
                                                  Cell.CellType[] pCellTypes,
                                                  int pFrequency)
      ```
  - ### Method Detail


    - #### observe

      ```
      public void observe(PottsEngine.State engineState)
      ```

      Description copied from class: `Statistic`

      Observe values during MCS.

      Specified by:
      :   `observe` in class `Statistic`


    - #### getSeriesNames

      ```
      public java.lang.String[] getSeriesNames()
      ```

      Overrides:
      :   `getSeriesNames` in class `TypeSpecificCellStatistic`


Skip navigation links


- Overview
- Package
- Class
- Use
- Tree
- Deprecated
- Index
- Help

- Prev Class
- Next Class

- Frames
- No Frames

- All Classes

- Summary:
- Nested |
- Field |
- Constr |
- Method

- Detail:
- Field |
- Constr |
- Method
