# Supplementary material for: Sorting at embryonic boundaries requires high heterotypic interfacial tension
Source: Nat Commun. 2017 Jul 31;8:157. doi: 10.1038/s41467-017-00146-x (PMC5537356; doi:10.1038/s41467-017-00146-x)
Supplement: Supplementary file 2 — Supplementary Software 1 [file 41467_2017_146_MOESM2_ESM.zip › PottsModel/SrcPottsModel/doc/engine/TypeSpecificNumNeighborsStatistic.html]

TypeSpecificNumNeighborsStatistic


JavaScript is disabled on your browser.


Skip navigation links


- Overview
- Package
- Class
- Use
- Tree
- Deprecated
- Index
- Help

- Prev Class
- Next Class

- Frames
- No Frames

- All Classes

- Summary:
- Nested |
- Field |
- Constr |
- Method

- Detail:
- Field |
- Constr |
- Method


engine

## Class TypeSpecificNumNeighborsStatistic

- java.lang.Object
- - engine.Statistic
  - - engine.TypeSpecificCellStatistic
    - - engine.TypeSpecificNumNeighborsStatistic

- ---

    

  ```
  public class TypeSpecificNumNeighborsStatistic
  extends TypeSpecificCellStatistic
  ```

- - ### Nested Class Summary

    - ### Nested classes/interfaces inherited from class engine.Statistic

      `Statistic.Utils`
  - ### Field Summary

    - ### Fields inherited from class engine.Statistic

      `DEFAULT_FREQUENCY`
  - ### Constructor Summary

    Constructors

    | Constructor and Description |
    | `TypeSpecificNumNeighborsStatistic(PottsEngine pEngine, java.lang.String pYAxisLabel, Cell.CellType[] pCellTypes, int pFrequency)` |
  - ### Method Summary

    All Methods Instance Methods Concrete Methods

    | Modifier and Type | Method and Description |
    | `void` | `observe(PottsEngine.State engineState)` Observe values during MCS. |

    - ### Methods inherited from class engine.TypeSpecificCellStatistic

      `getSeriesNames`
    - ### Methods inherited from class engine.Statistic

      `addToManagerStatistics, attachPlotPanel, getAxis, getEngine, getFrequency, getLastValues, getYAxisLabel, isAreaRendered, isDisplayed, isSpinAttemptsObserved, setRepeatFrequency, wrapUp`
    - ### Methods inherited from class java.lang.Object

      `equals, getClass, hashCode, notify, notifyAll, toString, wait, wait, wait`

- - ### Constructor Detail


    - #### TypeSpecificNumNeighborsStatistic

      ```
      public TypeSpecificNumNeighborsStatistic(PottsEngine pEngine,
                                               java.lang.String pYAxisLabel,
                                               Cell.CellType[] pCellTypes,
                                               int pFrequency)
      ```
  - ### Method Detail


    - #### observe

      ```
      public void observe(PottsEngine.State engineState)
      ```

      Description copied from class: `Statistic`

      Observe values during MCS.

      Specified by:
      :   `observe` in class `Statistic`


Skip navigation links


- Overview
- Package
- Class
- Use
- Tree
- Deprecated
- Index
- Help

- Prev Class
- Next Class

- Frames
- No Frames

- All Classes

- Summary:
- Nested |
- Field |
- Constr |
- Method

- Detail:
- Field |
- Constr |
- Method
