# Supplementary material for: Sorting at embryonic boundaries requires high heterotypic interfacial tension
Source: Nat Commun. 2017 Jul 31;8:157. doi: 10.1038/s41467-017-00146-x (PMC5537356; doi:10.1038/s41467-017-00146-x)
Supplement: Supplementary file 2 — Supplementary Software 1 [file 41467_2017_146_MOESM2_ESM.zip › PottsModel/SrcPottsModel/doc/engine/Utils.EnergyTracker.html]

Utils.EnergyTracker


JavaScript is disabled on your browser.


Skip navigation links


- Overview
- Package
- Class
- Use
- Tree
- Deprecated
- Index
- Help

- Prev Class
- Next Class

- Frames
- No Frames

- All Classes

- Summary:
- Nested |
- Field |
- Constr |
- Method

- Detail:
- Field |
- Constr |
- Method


engine

## Class Utils.EnergyTracker

- java.lang.Object
- - engine.Utils.EnergyTracker

- Enclosing class:
  :   Utils

  ---

    

  ```
  public static class Utils.EnergyTracker
  extends java.lang.Object
  ```

- - ### Constructor Summary

    Constructors

    | Constructor and Description |
    | `EnergyTracker(java.util.List<EnergyFunction.Energy> energyTypes)` |
  - ### Method Summary

    All Methods Instance Methods Concrete Methods

    | Modifier and Type | Method and Description |
    | `void` | `add(EnergyFunction.Energy t, double value)` |
    | `java.util.List<EnergyFunction.Energy>` | `getEnergyTypes()` |
    | `double` | `mean(EnergyFunction.Energy t)` |
    | `void` | `reset()` |
    | `double` | `std(EnergyFunction.Energy t)` |

    - ### Methods inherited from class java.lang.Object

      `equals, getClass, hashCode, notify, notifyAll, toString, wait, wait, wait`

- - ### Constructor Detail


    - #### EnergyTracker

      ```
      public EnergyTracker(java.util.List<EnergyFunction.Energy> energyTypes)
      ```
  - ### Method Detail


    - #### getEnergyTypes

      ```
      public java.util.List<EnergyFunction.Energy> getEnergyTypes()
      ```


    - #### add

      ```
      public void add(EnergyFunction.Energy t,
                      double value)
      ```


    - #### mean

      ```
      public double mean(EnergyFunction.Energy t)
      ```


    - #### std

      ```
      public double std(EnergyFunction.Energy t)
      ```


    - #### reset

      ```
      public void reset()
      ```


Skip navigation links


- Overview
- Package
- Class
- Use
- Tree
- Deprecated
- Index
- Help

- Prev Class
- Next Class

- Frames
- No Frames

- All Classes

- Summary:
- Nested |
- Field |
- Constr |
- Method

- Detail:
- Field |
- Constr |
- Method
