# Supplementary material for: Sorting at embryonic boundaries requires high heterotypic interfacial tension
Source: Nat Commun. 2017 Jul 31;8:157. doi: 10.1038/s41467-017-00146-x (PMC5537356; doi:10.1038/s41467-017-00146-x)
Supplement: Supplementary file 2 — Supplementary Software 1 [file 41467_2017_146_MOESM2_ESM.zip › PottsModel/SrcPottsModel/doc/gui/Arrow.html]

Arrow


---


|  |  |  |  |  |  |  |  |  |  |  |
| --- | --- | --- | --- | --- | --- | --- | --- | --- | --- | --- |
| |  |  |  |  |  |  |  |  | | --- | --- | --- | --- | --- | --- | --- | --- | | **Overview** | **Package** | **Class** | **Use** | **Tree** | **Deprecated** | **Index** | **Help** | | |  |
| PREV CLASS   **NEXT CLASS** | **FRAMES**    **NO FRAMES**     **All Classes** |
| SUMMARY: NESTED | FIELD | CONSTR | METHOD | DETAIL: FIELD | CONSTR | METHOD |


---


## gui Class Arrow

```
java.lang.Object
  gui.Arrow
```

---

``` public class Arrow extends java.lang.Object ```

---

| **Field Summary** | |
| --- | --- |
| `(package private) static float` | `aArrowLength` |
| `(package private)  java.awt.Rectangle` | `aBounds` |
| `(package private) static float` | `aStemLength` |
| `(package private)  Vector` | `aVector` |
| `(package private) static float` | `cArrowRatio` |
| `(package private)  java.awt.Color` | `cColor` |
| `(package private)  boolean` | `isLengthPredefined` |


| **Constructor Summary** | |
| --- | --- |
| `Arrow(Vector pVector)` |
| `Arrow(Vector pVector, java.awt.Color pColor)` |


| **Method Summary** | |
| --- | --- |
| `private static java.awt.geom.Path2D` | `arrowtip(java.awt.Point p)`             Returns triangle arrow tip shooting from point p. |
| `void` | `destroyPreviousState(java.awt.Graphics2D g, java.awt.Color bg)` |
| `void` | `draw(java.awt.Graphics2D g, java.awt.Color bg)` |
| `java.awt.Rectangle` | `getBounds()` |
| `static void` | `main(java.lang.String... args)` |
| `void` | `setLength(float length)` |

| **Methods inherited from class java.lang.Object** |
| --- |
| `clone, equals, finalize, getClass, hashCode, notify, notifyAll, toString, wait, wait, wait` |

| **Field Detail** |
| --- |

### aVector

```
final Vector aVector
```

---


### aArrowLength

```
static float aArrowLength
```

---


### cArrowRatio

```
static final float cArrowRatio
```

**See Also:**: Constant Field Values

---


### aStemLength

```
static float aStemLength
```

---


### cColor

```
java.awt.Color cColor
```

---


### aBounds

```
java.awt.Rectangle aBounds
```

---


### isLengthPredefined

```
boolean isLengthPredefined
```


| **Constructor Detail** |
| --- |

### Arrow

```
public Arrow(Vector pVector)
```

---


### Arrow

```
public Arrow(Vector pVector,
             java.awt.Color pColor)
```


| **Method Detail** |
| --- |

### arrowtip

```
private static java.awt.geom.Path2D arrowtip(java.awt.Point p)
```

:   Returns triangle arrow tip shooting from point p.

    :   **Returns:**

---


### draw

```
public void draw(java.awt.Graphics2D g,
                 java.awt.Color bg)
```

---


### getBounds

```
public java.awt.Rectangle getBounds()
```

---


### destroyPreviousState

```
public void destroyPreviousState(java.awt.Graphics2D g,
                                 java.awt.Color bg)
```

---


### main

```
public static void main(java.lang.String... args)
```

---


### setLength

```
public void setLength(float length)
```


---


|  |  |  |  |  |  |  |  |  |  |  |
| --- | --- | --- | --- | --- | --- | --- | --- | --- | --- | --- |
| |  |  |  |  |  |  |  |  | | --- | --- | --- | --- | --- | --- | --- | --- | | **Overview** | **Package** | **Class** | **Use** | **Tree** | **Deprecated** | **Index** | **Help** | | |  |
| PREV CLASS   **NEXT CLASS** | **FRAMES**    **NO FRAMES**     **All Classes** |
| SUMMARY: NESTED | FIELD | CONSTR | METHOD | DETAIL: FIELD | CONSTR | METHOD |


---
