# Supplementary material for: Sorting at embryonic boundaries requires high heterotypic interfacial tension
Source: Nat Commun. 2017 Jul 31;8:157. doi: 10.1038/s41467-017-00146-x (PMC5537356; doi:10.1038/s41467-017-00146-x)
Supplement: Supplementary file 2 — Supplementary Software 1 [file 41467_2017_146_MOESM2_ESM.zip › PottsModel/SrcPottsModel/doc/gui/CellDisplay.CellDisplayCenter.html]

CellDisplay.CellDisplayCenter


---


|  |  |  |  |  |  |  |  |  |  |  |
| --- | --- | --- | --- | --- | --- | --- | --- | --- | --- | --- |
| |  |  |  |  |  |  |  |  | | --- | --- | --- | --- | --- | --- | --- | --- | | **Overview** | **Package** | **Class** | **Use** | **Tree** | **Deprecated** | **Index** | **Help** | | |  |
| **PREV CLASS**   **NEXT CLASS** | **FRAMES**    **NO FRAMES**     **All Classes** |
| SUMMARY: NESTED | FIELD | CONSTR | METHOD | DETAIL: FIELD | CONSTR | METHOD |


---


## gui Class CellDisplay.CellDisplayCenter

```
java.lang.Object
  gui.CellDisplay.CellDisplayCenter
```

**Enclosing class:**: CellDisplay

---

``` private class CellDisplay.CellDisplayCenter extends java.lang.Object ```

---

| **Field Summary** | |
| --- | --- |
| `(package private)  java.awt.geom.Rectangle2D` | `aBounds` |
| `(package private)  java.awt.Point` | `aCenter` |
| `(package private)  java.awt.geom.Ellipse2D` | `aCenterEllipse` |
| `(package private)  java.awt.Color` | `aCenterEllipseColor` |
| `(package private)  int` | `aSize` |


| **Constructor Summary** | |
| --- | --- |
| `CellDisplay.CellDisplayCenter(java.awt.Point pCenter)` |


| **Method Summary** | |
| --- | --- |
| `void` | `draw(java.awt.Graphics2D g, java.awt.Color bg)` |
| `void` | `updateLocation()` |

| **Methods inherited from class java.lang.Object** |
| --- |
| `clone, equals, finalize, getClass, hashCode, notify, notifyAll, toString, wait, wait, wait` |

| **Field Detail** |
| --- |

### aCenterEllipseColor

```
final java.awt.Color aCenterEllipseColor
```

---


### aSize

```
final int aSize
```

**See Also:**: Constant Field Values

---


### aCenter

```
final java.awt.Point aCenter
```

---


### aBounds

```
java.awt.geom.Rectangle2D aBounds
```

---


### aCenterEllipse

```
java.awt.geom.Ellipse2D aCenterEllipse
```


| **Constructor Detail** |
| --- |

### CellDisplay.CellDisplayCenter

```
public CellDisplay.CellDisplayCenter(java.awt.Point pCenter)
```


| **Method Detail** |
| --- |

### updateLocation

```
public void updateLocation()
```

---


### draw

```
public void draw(java.awt.Graphics2D g,
                 java.awt.Color bg)
```


---


|  |  |  |  |  |  |  |  |  |  |  |
| --- | --- | --- | --- | --- | --- | --- | --- | --- | --- | --- |
| |  |  |  |  |  |  |  |  | | --- | --- | --- | --- | --- | --- | --- | --- | | **Overview** | **Package** | **Class** | **Use** | **Tree** | **Deprecated** | **Index** | **Help** | | |  |
| **PREV CLASS**   **NEXT CLASS** | **FRAMES**    **NO FRAMES**     **All Classes** |
| SUMMARY: NESTED | FIELD | CONSTR | METHOD | DETAIL: FIELD | CONSTR | METHOD |


---
