# Supplementary material for: Sorting at embryonic boundaries requires high heterotypic interfacial tension
Source: Nat Commun. 2017 Jul 31;8:157. doi: 10.1038/s41467-017-00146-x (PMC5537356; doi:10.1038/s41467-017-00146-x)
Supplement: Supplementary file 2 — Supplementary Software 1 [file 41467_2017_146_MOESM2_ESM.zip › PottsModel/SrcPottsModel/doc/gui/CellDisplay.html]

CellDisplay


JavaScript is disabled on your browser.


Skip navigation links


- Overview
- Package
- Class
- Use
- Tree
- Deprecated
- Index
- Help

- Prev Class
- Next Class

- Frames
- No Frames

- All Classes

- Summary:
- Nested |
- Field |
- Constr |
- Method

- Detail:
- Field |
- Constr |
- Method


gui

## Class CellDisplay

- java.lang.Object
- - gui.CellDisplay

- ---

    

  ```
  public class CellDisplay
  extends java.lang.Object
  ```

- - ### Constructor Summary

    Constructors

    | Constructor and Description |
    | `CellDisplay(Cell pCell)` |
  - ### Method Summary

    All Methods Instance Methods Concrete Methods

    | Modifier and Type | Method and Description |
    | `void` | `addOrRemovePixel(Pixel pPixel, boolean pIsAdded)` |
    | `void` | `draw(java.awt.Graphics2D g)` |
    | `java.awt.Point` | `getCenter()` |
    | `int` | `hashCode()` |

    - ### Methods inherited from class java.lang.Object

      `equals, getClass, notify, notifyAll, toString, wait, wait, wait`

- - ### Constructor Detail


    - #### CellDisplay

      ```
      public CellDisplay(Cell pCell)
      ```
  - ### Method Detail


    - #### draw

      ```
      public void draw(java.awt.Graphics2D g)
      ```


    - #### hashCode

      ```
      public int hashCode()
      ```

      Overrides:
      :   `hashCode` in class `java.lang.Object`


    - #### getCenter

      ```
      public java.awt.Point getCenter()
      ```


    - #### addOrRemovePixel

      ```
      public void addOrRemovePixel(Pixel pPixel,
                                   boolean pIsAdded)
      ```


Skip navigation links


- Overview
- Package
- Class
- Use
- Tree
- Deprecated
- Index
- Help

- Prev Class
- Next Class

- Frames
- No Frames

- All Classes

- Summary:
- Nested |
- Field |
- Constr |
- Method

- Detail:
- Field |
- Constr |
- Method
