# Supplementary material for: Sorting at embryonic boundaries requires high heterotypic interfacial tension
Source: Nat Commun. 2017 Jul 31;8:157. doi: 10.1038/s41467-017-00146-x (PMC5537356; doi:10.1038/s41467-017-00146-x)
Supplement: Supplementary file 2 — Supplementary Software 1 [file 41467_2017_146_MOESM2_ESM.zip › PottsModel/SrcPottsModel/doc/gui/class-use/ConfigurationPanel.Property.html]

Uses of Class gui.ConfigurationPanel.Property


---


|  |  |  |  |  |  |  |  |  |  |  |
| --- | --- | --- | --- | --- | --- | --- | --- | --- | --- | --- |
| |  |  |  |  |  |  |  |  | | --- | --- | --- | --- | --- | --- | --- | --- | | **Overview** | **Package** | **Class** | **Use** | **Tree** | **Deprecated** | **Index** | **Help** | | |  |
| PREV   NEXT | **FRAMES**    **NO FRAMES**     **All Classes** |


---


## **Uses of Class gui.ConfigurationPanel.Property**

| Packages that use ConfigurationPanel.Property | |
| --- | --- |
| **gui** |  |

| Uses of ConfigurationPanel.Property in gui | |
| --- | --- |

| Methods in gui that return ConfigurationPanel.Property | |
| --- | --- |
| `static ConfigurationPanel.Property` | `ConfigurationPanel.Property.valueOf(java.lang.String name)`             Returns the enum constant of this type with the specified name. |
| `static ConfigurationPanel.Property[]` | `ConfigurationPanel.Property.values()`             Returns an array containing the constants of this enum type, in the order they are declared. |

| Constructors in gui with parameters of type ConfigurationPanel.Property | |
| --- | --- |
| `ConfigurationPanel.NumberSpinner(ConfigurationPanel.Property p)` |
| `ConfigurationPanel.SpinnerPanel(java.lang.String title, ConfigurationPanel.Property[] properties)` |
| `ConfigurationPanel.ToggleButton(ConfigurationPanel.Property p)` |
| `ConfigurationPanel.TogglePanel(java.lang.String title, ConfigurationPanel.Property[] properties)` |

---


|  |  |  |  |  |  |  |  |  |  |  |
| --- | --- | --- | --- | --- | --- | --- | --- | --- | --- | --- |
| |  |  |  |  |  |  |  |  | | --- | --- | --- | --- | --- | --- | --- | --- | | **Overview** | **Package** | **Class** | **Use** | **Tree** | **Deprecated** | **Index** | **Help** | | |  |
| PREV   NEXT | **FRAMES**    **NO FRAMES**     **All Classes** |


---
