# Supplementary material for: Sorting at embryonic boundaries requires high heterotypic interfacial tension
Source: Nat Commun. 2017 Jul 31;8:157. doi: 10.1038/s41467-017-00146-x (PMC5537356; doi:10.1038/s41467-017-00146-x)
Supplement: Supplementary file 2 — Supplementary Software 1 [file 41467_2017_146_MOESM2_ESM.zip › PottsModel/SrcPottsModel/doc/gui/class-use/Hexagon.CubeCoordinates.html]

Uses of Class gui.Hexagon.CubeCoordinates


JavaScript is disabled on your browser.


Skip navigation links


- Overview
- Package
- Class
- Use
- Tree
- Deprecated
- Index
- Help

- Prev
- Next

- Frames
- No Frames

- All Classes

## Uses of Class gui.Hexagon.CubeCoordinates

- Packages that use Hexagon.CubeCoordinates

  | Package | Description |
  |  |  |
  | --- | --- |
  | gui |  |
- - ### Uses of Hexagon.CubeCoordinates in gui

    Methods in gui that return Hexagon.CubeCoordinates

    | Modifier and Type | Method and Description |
    |  |  |
    | --- | --- |
    | `Hexagon.CubeCoordinates` | Hexagon.CubeCoordinates.`getDiagonalNeighborCoordinates(IPositionManager.Position p)` |
    | `Hexagon.CubeCoordinates` | Hexagon.CubeCoordinates.`getNeighborCoordinates(PixelShape.Edge p)` |
    | `Hexagon.CubeCoordinates` | Hexagon.OffsetCoordinates.`toCube()` |
    | `Hexagon.CubeCoordinates` | Hexagon.AxialCoordinates.`toCube()` |

    Methods in gui with parameters of type Hexagon.CubeCoordinates

    | Modifier and Type | Method and Description |
    |  |  |
    | --- | --- |
    | `static double` | Hexagon.`distance(Hexagon.CubeCoordinates c1, Hexagon.CubeCoordinates c2)` |

Skip navigation links


- Overview
- Package
- Class
- Use
- Tree
- Deprecated
- Index
- Help

- Prev
- Next

- Frames
- No Frames

- All Classes
