# Supplementary material for: Sorting at embryonic boundaries requires high heterotypic interfacial tension
Source: Nat Commun. 2017 Jul 31;8:157. doi: 10.1038/s41467-017-00146-x (PMC5537356; doi:10.1038/s41467-017-00146-x)
Supplement: Supplementary file 2 — Supplementary Software 1 [file 41467_2017_146_MOESM2_ESM.zip › PottsModel/SrcPottsModel/doc/gui/class-use/PixelShape.Edge.html]

Uses of Class gui.PixelShape.Edge


JavaScript is disabled on your browser.


Skip navigation links


- Overview
- Package
- Class
- Use
- Tree
- Deprecated
- Index
- Help

- Prev
- Next

- Frames
- No Frames

- All Classes

## Uses of Class gui.PixelShape.Edge

- Packages that use PixelShape.Edge

  | Package | Description |
  |  |  |
  | --- | --- |
  | gui |  |
  | model |  |
- - ### Uses of PixelShape.Edge in gui

    Methods in gui that return PixelShape.Edge

    | Modifier and Type | Method and Description |
    |  |  |
    | --- | --- |
    | `PixelShape.Edge[]` | SquarePixelDisplay.`getValidEdges()` |
    | `abstract PixelShape.Edge[]` | PixelDisplay.`getValidEdges()` |
    | `PixelShape.Edge[]` | HexagonPixelDisplay.`getValidEdges()` |
    | `static PixelShape.Edge` | PixelShape.Edge.`valueOf(java.lang.String name)` Returns the enum constant of this type with the specified name. |
    | `static PixelShape.Edge[]` | PixelShape.Edge.`values()` Returns an array containing the constants of this enum type, in the order they are declared. |

    Methods in gui with parameters of type PixelShape.Edge

    | Modifier and Type | Method and Description |
    |  |  |
    | --- | --- |
    | `void` | PixelDisplay.`drawEdge(java.awt.Graphics2D g, PixelShape.Edge pEdge, boolean pActive)` Set edge as active/inactive and then draw it. |
    | `java.awt.geom.Line2D` | Square.`getEdge(PixelShape.Edge pEdge)` |
    | `java.awt.geom.Line2D` | PixelShape.`getEdge(PixelShape.Edge pEdge)` |
    | `java.awt.geom.Line2D` | Hexagon.`getEdge(PixelShape.Edge pEdge)` |
    | `int` | SquarePixelDisplay.`getEdgeIndex(PixelShape.Edge pEdge)` |
    | `int` | HexagonPixelDisplay.`getEdgeIndex(PixelShape.Edge pEdge)` |
    | `java.awt.geom.Line2D` | PixelDisplay.`getEdgeLine(PixelShape.Edge pEdge)` |
    | `Coordinates` | Square.`getNeighborCoordinates(PixelShape.Edge pEdge)` |
    | `Coordinates` | PixelShape.`getNeighborCoordinates(PixelShape.Edge pEdge)` |
    | `Coordinates` | Hexagon.`getNeighborCoordinates(PixelShape.Edge pEdge)` |
    | `Hexagon.OffsetCoordinates` | Hexagon.OffsetCoordinates.`getNeighborCoordinates(PixelShape.Edge p)` |
    | `Hexagon.AxialCoordinates` | Hexagon.AxialCoordinates.`getNeighborCoordinates(PixelShape.Edge p)` |
    | `Hexagon.CubeCoordinates` | Hexagon.CubeCoordinates.`getNeighborCoordinates(PixelShape.Edge p)` |
    | `boolean` | PixelDisplay.`isActive(PixelShape.Edge pEdge)` |
    | `boolean` | SquarePixelDisplay.`isValidEdge(PixelShape.Edge pEdge)` |
    | `abstract boolean` | PixelDisplay.`isValidEdge(PixelShape.Edge pEdge)` |
    | `boolean` | HexagonPixelDisplay.`isValidEdge(PixelShape.Edge pEdge)` |
    | `void` | PottsCanvas.`setEdge(int x, int y, PixelShape.Edge pEdge, boolean pActive)` Draw pixel at (x,y) with given color. |
  - ### Uses of PixelShape.Edge in model

    Methods in model with parameters of type PixelShape.Edge

    | Modifier and Type | Method and Description |
    |  |  |
    | --- | --- |
    | `CartesianCoordinates` | CartesianCoordinates.`getNeighborCoordinates(PixelShape.Edge p)` |

Skip navigation links


- Overview
- Package
- Class
- Use
- Tree
- Deprecated
- Index
- Help

- Prev
- Next

- Frames
- No Frames

- All Classes
