# Supplementary material for: Sorting at embryonic boundaries requires high heterotypic interfacial tension
Source: Nat Commun. 2017 Jul 31;8:157. doi: 10.1038/s41467-017-00146-x (PMC5537356; doi:10.1038/s41467-017-00146-x)
Supplement: Supplementary file 2 — Supplementary Software 1 [file 41467_2017_146_MOESM2_ESM.zip › PottsModel/SrcPottsModel/doc/gui/class-use/PixelShape.html]

Uses of Interface gui.PixelShape


JavaScript is disabled on your browser.


Skip navigation links


- Overview
- Package
- Class
- Use
- Tree
- Deprecated
- Index
- Help

- Prev
- Next

- Frames
- No Frames

- All Classes

## Uses of Interface gui.PixelShape

- Packages that use PixelShape

  | Package | Description |
  |  |  |
  | --- | --- |
  | gui |  |
- - ### Uses of PixelShape in gui

    Classes in gui that implement PixelShape

    | Modifier and Type | Class and Description |
    |  |  |
    | --- | --- |
    | `class` | `Hexagon` Flat topped hexagon. |
    | `class` | `Square` |

    Methods in gui that return PixelShape

    | Modifier and Type | Method and Description |
    |  |  |
    | --- | --- |
    | `PixelShape` | PixelDisplay.`getShape()` |

Skip navigation links


- Overview
- Package
- Class
- Use
- Tree
- Deprecated
- Index
- Help

- Prev
- Next

- Frames
- No Frames

- All Classes
