# Supplementary material for: Sorting at embryonic boundaries requires high heterotypic interfacial tension
Source: Nat Commun. 2017 Jul 31;8:157. doi: 10.1038/s41467-017-00146-x (PMC5537356; doi:10.1038/s41467-017-00146-x)
Supplement: Supplementary file 2 — Supplementary Software 1 [file 41467_2017_146_MOESM2_ESM.zip › PottsModel/SrcPottsModel/doc/gui/class-use/PixelShape.Type.html]

Uses of Class gui.PixelShape.Type


JavaScript is disabled on your browser.


Skip navigation links


- Overview
- Package
- Class
- Use
- Tree
- Deprecated
- Index
- Help

- Prev
- Next

- Frames
- No Frames

- All Classes

## Uses of Class gui.PixelShape.Type

- Packages that use PixelShape.Type

  | Package | Description |
  |  |  |
  | --- | --- |
  | gui |  |
  | model |  |
- - ### Uses of PixelShape.Type in gui

    Methods in gui that return PixelShape.Type

    | Modifier and Type | Method and Description |
    |  |  |
    | --- | --- |
    | `PixelShape.Type` | Square.`getShapeType()` |
    | `PixelShape.Type` | PixelShape.`getShapeType()` |
    | `PixelShape.Type` | Hexagon.`getShapeType()` |
    | `static PixelShape.Type` | PixelShape.Type.`valueOf(java.lang.String name)` Returns the enum constant of this type with the specified name. |
    | `static PixelShape.Type[]` | PixelShape.Type.`values()` Returns an array containing the constants of this enum type, in the order they are declared. |
  - ### Uses of PixelShape.Type in model

    Fields in model declared as PixelShape.Type

    | Modifier and Type | Field and Description |
    |  |  |
    | --- | --- |
    | `PixelShape.Type` | Constants.ParameterManager.`pixelType` |

    Methods in model with parameters of type PixelShape.Type

    | Modifier and Type | Method and Description |
    |  |  |
    | --- | --- |
    | `static double` | Pixel.`getAreaUnit(PixelShape.Type pType)` |
    | `static double` | Pixel.`getEdgeUnit(PixelShape.Type pType)` |
    | `static int` | Pixel.`getNumPixelEdges(PixelShape.Type pType)` |
    | `void` | Constants.`setPixelType(PixelShape.Type pType)` |

Skip navigation links


- Overview
- Package
- Class
- Use
- Tree
- Deprecated
- Index
- Help

- Prev
- Next

- Frames
- No Frames

- All Classes
