# Supplementary material for: Sorting at embryonic boundaries requires high heterotypic interfacial tension
Source: Nat Commun. 2017 Jul 31;8:157. doi: 10.1038/s41467-017-00146-x (PMC5537356; doi:10.1038/s41467-017-00146-x)
Supplement: Supplementary file 2 — Supplementary Software 1 [file 41467_2017_146_MOESM2_ESM.zip › PottsModel/SrcPottsModel/doc/gui/ConfigurationPanel.Property.html]

ConfigurationPanel.Property


---


|  |  |  |  |  |  |  |  |  |  |  |
| --- | --- | --- | --- | --- | --- | --- | --- | --- | --- | --- |
| |  |  |  |  |  |  |  |  | | --- | --- | --- | --- | --- | --- | --- | --- | | **Overview** | **Package** | **Class** | **Use** | **Tree** | **Deprecated** | **Index** | **Help** | | |  |
| **PREV CLASS**   **NEXT CLASS** | **FRAMES**    **NO FRAMES**     **All Classes** |
| SUMMARY: NESTED | ENUM CONSTANTS | FIELD | METHOD | DETAIL: ENUM CONSTANTS | FIELD | METHOD |


---


## gui Enum ConfigurationPanel.Property

```
java.lang.Object
  java.lang.Enum<ConfigurationPanel.Property>
      gui.ConfigurationPanel.Property
```

**All Implemented Interfaces:**: java.io.Serializable, java.lang.Comparable<ConfigurationPanel.Property>

**Enclosing class:**: ConfigurationPanel

---

``` private static enum ConfigurationPanel.Property extends java.lang.Enum<ConfigurationPanel.Property> ```

---

| **Enum Constant Summary** | |
| --- | --- |
| `areaElasticity` |
| `cellMediumEnergy` |
| `heatMapMode` |
| `perimeterElasticity` |
| `protrusionElasticity` |
| `targetArea` |
| `targetPerimeter` |


| **Method Summary** | |
| --- | --- |
| `static ConfigurationPanel.Property` | `valueOf(java.lang.String name)`             Returns the enum constant of this type with the specified name. |
| `static ConfigurationPanel.Property[]` | `values()`             Returns an array containing the constants of this enum type, in the order they are declared. |

| **Methods inherited from class java.lang.Enum** |
| --- |
| `clone, compareTo, equals, finalize, getDeclaringClass, hashCode, name, ordinal, toString, valueOf` |

| **Methods inherited from class java.lang.Object** |
| --- |
| `getClass, notify, notifyAll, wait, wait, wait` |

| **Enum Constant Detail** |
| --- |

### heatMapMode

```
public static final ConfigurationPanel.Property heatMapMode
```

---


### targetArea

```
public static final ConfigurationPanel.Property targetArea
```

---


### targetPerimeter

```
public static final ConfigurationPanel.Property targetPerimeter
```

---


### areaElasticity

```
public static final ConfigurationPanel.Property areaElasticity
```

---


### perimeterElasticity

```
public static final ConfigurationPanel.Property perimeterElasticity
```

---


### protrusionElasticity

```
public static final ConfigurationPanel.Property protrusionElasticity
```

---


### cellMediumEnergy

```
public static final ConfigurationPanel.Property cellMediumEnergy
```


| **Method Detail** |
| --- |

### values

```
public static ConfigurationPanel.Property[] values()
```

:   Returns an array containing the constants of this enum type, in
    the order they are declared. This method may be used to iterate
    over the constants as follows:

    ```
    for (ConfigurationPanel.Property c : ConfigurationPanel.Property.values())
        System.out.println(c);
    ```

    :   **Returns:**: an array containing the constants of this enum type, in the order they are declared

---


### valueOf

```
public static ConfigurationPanel.Property valueOf(java.lang.String name)
```

:   Returns the enum constant of this type with the specified name.
    The string must match *exactly* an identifier used to declare an
    enum constant in this type. (Extraneous whitespace characters are
    not permitted.)

    :   **Parameters:**: `name` - the name of the enum constant to be returned. **Returns:**: the enum constant with the specified name **Throws:**: `java.lang.IllegalArgumentException` - if this enum type has no constant with the specified name: `java.lang.NullPointerException` - if the argument is null


---


|  |  |  |  |  |  |  |  |  |  |  |
| --- | --- | --- | --- | --- | --- | --- | --- | --- | --- | --- |
| |  |  |  |  |  |  |  |  | | --- | --- | --- | --- | --- | --- | --- | --- | | **Overview** | **Package** | **Class** | **Use** | **Tree** | **Deprecated** | **Index** | **Help** | | |  |
| **PREV CLASS**   **NEXT CLASS** | **FRAMES**    **NO FRAMES**     **All Classes** |
| SUMMARY: NESTED | ENUM CONSTANTS | FIELD | METHOD | DETAIL: ENUM CONSTANTS | FIELD | METHOD |


---
