# Supplementary material for: Sorting at embryonic boundaries requires high heterotypic interfacial tension
Source: Nat Commun. 2017 Jul 31;8:157. doi: 10.1038/s41467-017-00146-x (PMC5537356; doi:10.1038/s41467-017-00146-x)
Supplement: Supplementary file 2 — Supplementary Software 1 [file 41467_2017_146_MOESM2_ESM.zip › PottsModel/SrcPottsModel/doc/gui/Hexagon.html]

Hexagon


JavaScript is disabled on your browser.


Skip navigation links


- Overview
- Package
- Class
- Use
- Tree
- Deprecated
- Index
- Help

- Prev Class
- Next Class

- Frames
- No Frames

- All Classes

- Summary:
- Nested |
- Field |
- Constr |
- Method

- Detail:
- Field |
- Constr |
- Method


gui

## Class Hexagon

- java.lang.Object
- - gui.Hexagon

- All Implemented Interfaces:
  :   PixelShape

  ---

    

  ```
  public class Hexagon
  extends java.lang.Object
  implements PixelShape
  ```

  Flat topped hexagon.

  Author:
  :   eleyine

- - ### Nested Class Summary

    Nested Classes

    | Modifier and Type | Class and Description |
    | `class` | `Hexagon.AxialCoordinates` |
    | `class` | `Hexagon.CubeCoordinates` |
    | `class` | `Hexagon.OffsetCoordinates` Even-q vertical layout. |

    - ### Nested classes/interfaces inherited from interface gui.PixelShape

      `PixelShape.Edge, PixelShape.Type`
  - ### Constructor Summary

    Constructors

    | Constructor and Description |
    | `Hexagon(int pX, int pY, int pSize)` |
  - ### Method Summary

    All Methods Static Methods Instance Methods Concrete Methods

    | Modifier and Type | Method and Description |
    | `static double` | `distance(Hexagon.AxialCoordinates c1, Hexagon.AxialCoordinates c2)` |
    | `static double` | `distance(Hexagon.CubeCoordinates c1, Hexagon.CubeCoordinates c2)` |
    | `static double` | `distance(Hexagon.OffsetCoordinates c1, Hexagon.OffsetCoordinates c2)` |
    | `java.awt.Shape` | `getAWTShape()` |
    | `java.awt.Point` | `getCenter()` |
    | `Coordinates` | `getCoordinates()` |
    | `java.awt.Point` | `getCorner(int i)` |
    | `java.awt.geom.Line2D` | `getEdge(PixelShape.Edge pEdge)` |
    | `Coordinates` | `getNeighborCoordinates(PixelShape.Edge pEdge)` |
    | `PixelShape.Type` | `getShapeType()` |
    | `double` | `getX()` Get Hexagon center's Cartesian X coordinate. |
    | `double` | `getY()` Get Hexagon center's Cartesian Y coordinate. |

    - ### Methods inherited from class java.lang.Object

      `equals, getClass, hashCode, notify, notifyAll, toString, wait, wait, wait`

- - ### Constructor Detail


    - #### Hexagon

      ```
      public Hexagon(int pX,
                     int pY,
                     int pSize)
      ```
  - ### Method Detail


    - #### getX

      ```
      public double getX()
      ```

      Get Hexagon center's Cartesian X coordinate.


    - #### getY

      ```
      public double getY()
      ```

      Get Hexagon center's Cartesian Y coordinate.


    - #### getCorner

      ```
      public java.awt.Point getCorner(int i)
      ```


    - #### getCenter

      ```
      public java.awt.Point getCenter()
      ```

      Specified by:
      :   `getCenter` in interface `PixelShape`


    - #### getAWTShape

      ```
      public java.awt.Shape getAWTShape()
      ```

      Specified by:
      :   `getAWTShape` in interface `PixelShape`


    - #### getEdge

      ```
      public java.awt.geom.Line2D getEdge(PixelShape.Edge pEdge)
      ```

      Specified by:
      :   `getEdge` in interface `PixelShape`


    - #### distance

      ```
      public static double distance(Hexagon.CubeCoordinates c1,
                                    Hexagon.CubeCoordinates c2)
      ```


    - #### distance

      ```
      public static double distance(Hexagon.AxialCoordinates c1,
                                    Hexagon.AxialCoordinates c2)
      ```


    - #### distance

      ```
      public static double distance(Hexagon.OffsetCoordinates c1,
                                    Hexagon.OffsetCoordinates c2)
      ```


    - #### getCoordinates

      ```
      public Coordinates getCoordinates()
      ```

      Specified by:
      :   `getCoordinates` in interface `PixelShape`


    - #### getNeighborCoordinates

      ```
      public Coordinates getNeighborCoordinates(PixelShape.Edge pEdge)
      ```

      Specified by:
      :   `getNeighborCoordinates` in interface `PixelShape`


    - #### getShapeType

      ```
      public PixelShape.Type getShapeType()
      ```

      Specified by:
      :   `getShapeType` in interface `PixelShape`


Skip navigation links


- Overview
- Package
- Class
- Use
- Tree
- Deprecated
- Index
- Help

- Prev Class
- Next Class

- Frames
- No Frames

- All Classes

- Summary:
- Nested |
- Field |
- Constr |
- Method

- Detail:
- Field |
- Constr |
- Method
