# Supplementary material for: Sorting at embryonic boundaries requires high heterotypic interfacial tension
Source: Nat Commun. 2017 Jul 31;8:157. doi: 10.1038/s41467-017-00146-x (PMC5537356; doi:10.1038/s41467-017-00146-x)
Supplement: Supplementary file 2 — Supplementary Software 1 [file 41467_2017_146_MOESM2_ESM.zip › PottsModel/SrcPottsModel/doc/gui/Hexagon.OffsetCoordinates.html]

Hexagon.OffsetCoordinates


JavaScript is disabled on your browser.


Skip navigation links


- Overview
- Package
- Class
- Use
- Tree
- Deprecated
- Index
- Help

- Prev Class
- Next Class

- Frames
- No Frames

- All Classes

- Summary:
- Nested |
- Field |
- Constr |
- Method

- Detail:
- Field |
- Constr |
- Method


gui

## Class Hexagon.OffsetCoordinates

- java.lang.Object
- - gui.Hexagon.OffsetCoordinates

- All Implemented Interfaces:
  :   Coordinates

  Enclosing class:
  :   Hexagon

  ---

    

  ```
  public class Hexagon.OffsetCoordinates
  extends java.lang.Object
  implements Coordinates
  ```

  Even-q vertical layout.

  Author:
  :   eleyine

- - ### Constructor Summary

    Constructors

    | Constructor and Description |
    | `OffsetCoordinates(double q, double r)` |
  - ### Method Summary

    All Methods Instance Methods Concrete Methods

    | Modifier and Type | Method and Description |
    | `Hexagon.OffsetCoordinates` | `getNeighborCoordinates(PixelShape.Edge p)` |
    | `double` | `getX()` |
    | `double` | `getY()` |
    | `double` | `getZ()` |
    | `void` | `setX(double pX)` |
    | `void` | `setY(double pY)` |
    | `void` | `setZ(double pZ)` |
    | `Hexagon.AxialCoordinates` | `toAxial()` |
    | `Hexagon.CubeCoordinates` | `toCube()` |
    | `java.lang.String` | `toString()` |

    - ### Methods inherited from class java.lang.Object

      `equals, getClass, hashCode, notify, notifyAll, wait, wait, wait`

- - ### Constructor Detail


    - #### OffsetCoordinates

      ```
      public OffsetCoordinates(double q,
                               double r)
      ```
  - ### Method Detail


    - #### toCube

      ```
      public Hexagon.CubeCoordinates toCube()
      ```


    - #### toAxial

      ```
      public Hexagon.AxialCoordinates toAxial()
      ```


    - #### getNeighborCoordinates

      ```
      public Hexagon.OffsetCoordinates getNeighborCoordinates(PixelShape.Edge p)
      ```


    - #### getX

      ```
      public double getX()
      ```

      Specified by:
      :   `getX` in interface `Coordinates`


    - #### getY

      ```
      public double getY()
      ```

      Specified by:
      :   `getY` in interface `Coordinates`


    - #### getZ

      ```
      public double getZ()
      ```

      Specified by:
      :   `getZ` in interface `Coordinates`


    - #### toString

      ```
      public java.lang.String toString()
      ```

      Specified by:
      :   `toString` in interface `Coordinates`

      Overrides:
      :   `toString` in class `java.lang.Object`


    - #### setX

      ```
      public void setX(double pX)
      ```

      Specified by:
      :   `setX` in interface `Coordinates`


    - #### setY

      ```
      public void setY(double pY)
      ```

      Specified by:
      :   `setY` in interface `Coordinates`


    - #### setZ

      ```
      public void setZ(double pZ)
      ```

      Specified by:
      :   `setZ` in interface `Coordinates`


Skip navigation links


- Overview
- Package
- Class
- Use
- Tree
- Deprecated
- Index
- Help

- Prev Class
- Next Class

- Frames
- No Frames

- All Classes

- Summary:
- Nested |
- Field |
- Constr |
- Method

- Detail:
- Field |
- Constr |
- Method
