# Supplementary material for: Sorting at embryonic boundaries requires high heterotypic interfacial tension
Source: Nat Commun. 2017 Jul 31;8:157. doi: 10.1038/s41467-017-00146-x (PMC5537356; doi:10.1038/s41467-017-00146-x)
Supplement: Supplementary file 2 — Supplementary Software 1 [file 41467_2017_146_MOESM2_ESM.zip › PottsModel/SrcPottsModel/doc/gui/package-frame.html]

gui


# gui

## Interfaces

- EngineObserverPanel
- PixelShape

## Classes

- CellDisplay
- ConfigurationInformationPanel
- ConfigurationPanel
- ConfigurationStartMenu
- Console
- Hexagon
- HexagonPixelDisplay
- PixelDisplay
- PlotPanel
- PottsCanvas
- PottsFrame
- PottsToolbar
- SnapshotManager
- Square
- SquarePixelDisplay
- StatusBar
- Utils

## Enums

- PixelShape.Edge
- PixelShape.Type
- PottsFrame.Action
