# Supplementary material for: Sorting at embryonic boundaries requires high heterotypic interfacial tension
Source: Nat Commun. 2017 Jul 31;8:157. doi: 10.1038/s41467-017-00146-x (PMC5537356; doi:10.1038/s41467-017-00146-x)
Supplement: Supplementary file 2 — Supplementary Software 1 [file 41467_2017_146_MOESM2_ESM.zip › PottsModel/SrcPottsModel/doc/gui/package-summary.html]

gui


JavaScript is disabled on your browser.


Skip navigation links


- Overview
- Package
- Class
- Use
- Tree
- Deprecated
- Index
- Help

- Prev Package
- Next Package

- Frames
- No Frames

- All Classes

# Package gui

- Interface Summary

  | Interface | Description |
  |  |  |
  | --- | --- |
  | EngineObserverPanel |  |
  | PixelShape | Graphics Managment. |
- Class Summary

  | Class | Description |
  |  |  |
  | --- | --- |
  | CellDisplay |  |
  | ConfigurationInformationPanel |  |
  | ConfigurationPanel |  |
  | ConfigurationStartMenu |  |
  | Console |  |
  | Hexagon | Flat topped hexagon. |
  | HexagonPixelDisplay |  |
  | PixelDisplay | Abstract class managing active edges and pixel colors. |
  | PlotPanel |  |
  | PottsCanvas | Graphical representation of the Potts Model Lattice. |
  | PottsFrame |  |
  | PottsToolbar |  |
  | SnapshotManager | Graphical representation of the Potts Model Lattice. |
  | Square |  |
  | SquarePixelDisplay |  |
  | StatusBar |  |
  | Utils |  |
- Enum Summary

  | Enum | Description |
  |  |  |
  | --- | --- |
  | PixelShape.Edge |  |
  | PixelShape.Type |  |
  | PottsFrame.Action |  |

Skip navigation links


- Overview
- Package
- Class
- Use
- Tree
- Deprecated
- Index
- Help

- Prev Package
- Next Package

- Frames
- No Frames

- All Classes
