# Supplementary material for: Sorting at embryonic boundaries requires high heterotypic interfacial tension
Source: Nat Commun. 2017 Jul 31;8:157. doi: 10.1038/s41467-017-00146-x (PMC5537356; doi:10.1038/s41467-017-00146-x)
Supplement: Supplementary file 2 — Supplementary Software 1 [file 41467_2017_146_MOESM2_ESM.zip › PottsModel/SrcPottsModel/doc/gui/package-tree.html]

gui Class Hierarchy


JavaScript is disabled on your browser.


Skip navigation links


- Overview
- Package
- Class
- Use
- Tree
- Deprecated
- Index
- Help

- Prev
- Next

- Frames
- No Frames

- All Classes

# Hierarchy For Package gui

Package Hierarchies:

- All Packages

## Class Hierarchy

- java.lang.Object
  - gui.CellDisplay
  - java.awt.Component (implements java.awt.image.ImageObserver, java.awt.MenuContainer, java.io.Serializable)
    - java.awt.Container
      - javax.swing.JComponent (implements java.io.Serializable)
        - javax.swing.JPanel (implements javax.accessibility.Accessible)
          - gui.ConfigurationInformationPanel (implements gui.EngineObserverPanel)
          - gui.ConfigurationPanel
          - gui.Console
          - gui.PlotPanel (implements mvc.Observer)
          - gui.PottsCanvas (implements mvc.Observer, java.beans.PropertyChangeListener)
          - gui.PottsToolbar
          - gui.StatusBar (implements gui.EngineObserverPanel, mvc.Observer)
      - java.awt.Window (implements javax.accessibility.Accessible)
        - java.awt.Frame (implements java.awt.MenuContainer)
          - javax.swing.JFrame (implements javax.accessibility.Accessible, javax.swing.RootPaneContainer, javax.swing.WindowConstants)
            - gui.PottsFrame (implements java.awt.event.ActionListener, mvc.Observer)
  - gui.ConfigurationStartMenu
  - gui.Hexagon (implements gui.PixelShape)
  - gui.Hexagon.AxialCoordinates (implements model.Coordinates)
  - gui.Hexagon.CubeCoordinates (implements model.Coordinates)
  - gui.Hexagon.OffsetCoordinates (implements model.Coordinates)
  - gui.PixelDisplay
    - gui.HexagonPixelDisplay
    - gui.SquarePixelDisplay
  - gui.SnapshotManager (implements mvc.Observer)
  - gui.Square (implements gui.PixelShape)
  - gui.Utils

## Interface Hierarchy

- gui.EngineObserverPanel
- gui.PixelShape

## Enum Hierarchy

- java.lang.Object
  - java.lang.Enum<E> (implements java.lang.Comparable<T>, java.io.Serializable)
    - gui.PottsFrame.Action
    - gui.PixelShape.Edge
    - gui.PixelShape.Type

Skip navigation links


- Overview
- Package
- Class
- Use
- Tree
- Deprecated
- Index
- Help

- Prev
- Next

- Frames
- No Frames

- All Classes
