# Supplementary material for: Sorting at embryonic boundaries requires high heterotypic interfacial tension
Source: Nat Commun. 2017 Jul 31;8:157. doi: 10.1038/s41467-017-00146-x (PMC5537356; doi:10.1038/s41467-017-00146-x)
Supplement: Supplementary file 2 — Supplementary Software 1 [file 41467_2017_146_MOESM2_ESM.zip › PottsModel/SrcPottsModel/doc/gui/package-use.html]

Uses of Package gui


JavaScript is disabled on your browser.


Skip navigation links


- Overview
- Package
- Class
- Use
- Tree
- Deprecated
- Index
- Help

- Prev
- Next

- Frames
- No Frames

- All Classes

# Uses of Package gui

- Packages that use gui

  | Package | Description |
  |  |  |
  | --- | --- |
  | engine |  |
  | gui |  |
  | model |  |
- Classes in gui used by engine

  | Class and Description |
  |  |
  | --- |
  | PlotPanel |
- Classes in gui used by gui

  | Class and Description |
  |  |
  | --- |
  | EngineObserverPanel |
  | Hexagon.AxialCoordinates |
  | Hexagon.CubeCoordinates |
  | Hexagon.OffsetCoordinates Even-q vertical layout. |
  | PixelDisplay Abstract class managing active edges and pixel colors. |
  | PixelShape Graphics Managment. |
  | PixelShape.Edge |
  | PixelShape.Type |
  | PottsCanvas Graphical representation of the Potts Model Lattice. |
  | PottsFrame |
  | PottsFrame.Action |
- Classes in gui used by model

  | Class and Description |
  |  |
  | --- |
  | CellDisplay |
  | PixelDisplay Abstract class managing active edges and pixel colors. |
  | PixelShape.Edge |
  | PixelShape.Type |
  | PottsCanvas Graphical representation of the Potts Model Lattice. |

Skip navigation links


- Overview
- Package
- Class
- Use
- Tree
- Deprecated
- Index
- Help

- Prev
- Next

- Frames
- No Frames

- All Classes
