# Supplementary material for: Sorting at embryonic boundaries requires high heterotypic interfacial tension
Source: Nat Commun. 2017 Jul 31;8:157. doi: 10.1038/s41467-017-00146-x (PMC5537356; doi:10.1038/s41467-017-00146-x)
Supplement: Supplementary file 2 — Supplementary Software 1 [file 41467_2017_146_MOESM2_ESM.zip › PottsModel/SrcPottsModel/doc/gui/PixelDisplay.html]

PixelDisplay


JavaScript is disabled on your browser.


Skip navigation links


- Overview
- Package
- Class
- Use
- Tree
- Deprecated
- Index
- Help

- Prev Class
- Next Class

- Frames
- No Frames

- All Classes

- Summary:
- Nested |
- Field |
- Constr |
- Method

- Detail:
- Field |
- Constr |
- Method


gui

## Class PixelDisplay

- java.lang.Object
- - gui.PixelDisplay

- Direct Known Subclasses:
  :   HexagonPixelDisplay, SquarePixelDisplay

  ---

    

  ```
  public abstract class PixelDisplay
  extends java.lang.Object
  ```

  Abstract class managing active edges and pixel colors.

  Author:
  :   eleyine

- - ### Field Summary

    Fields

    | Modifier and Type | Field and Description |
    | `java.awt.Color` | `aColor` |
  - ### Constructor Summary

    Constructors

    | Constructor and Description |
    | `PixelDisplay(int pX, int pY, int pUnitSize)` Initialize Pixel at location (x, y) |
    | `PixelDisplay(int pX, int pY, int pUnitSize, java.awt.Color pColor)` |
  - ### Method Summary

    All Methods Instance Methods Abstract Methods Concrete Methods

    | Modifier and Type | Method and Description |
    | `void` | `drawColor(java.awt.Graphics2D g)` |
    | `void` | `drawEdge(java.awt.Graphics2D g, PixelShape.Edge pEdge, boolean pActive)` Set edge as active/inactive and then draw it. |
    | `void` | `drawEdges(java.awt.Graphics2D g)` |
    | `java.awt.Shape` | `getAWTShape()` |
    | `java.awt.geom.Line2D` | `getEdgeLine(PixelShape.Edge pEdge)` |
    | `abstract int` | `getNumEdges()` |
    | `PixelShape` | `getShape()` |
    | `abstract PixelShape.Edge[]` | `getValidEdges()` |
    | `boolean` | `isActive(PixelShape.Edge pEdge)` |
    | `abstract boolean` | `isValidEdge(PixelShape.Edge pEdge)` |
    | `java.lang.String` | `toString()` |

    - ### Methods inherited from class java.lang.Object

      `equals, getClass, hashCode, notify, notifyAll, wait, wait, wait`

- - ### Field Detail


    - #### aColor

      ```
      public java.awt.Color aColor
      ```
  - ### Constructor Detail


    - #### PixelDisplay

      ```
      public PixelDisplay(int pX,
                          int pY,
                          int pUnitSize)
      ```

      Initialize Pixel at location (x, y)

      Parameters:
      :   `x` -
      :   `y` -


    - #### PixelDisplay

      ```
      public PixelDisplay(int pX,
                          int pY,
                          int pUnitSize,
                          java.awt.Color pColor)
      ```
  - ### Method Detail


    - #### drawEdge

      ```
      public void drawEdge(java.awt.Graphics2D g,
                           PixelShape.Edge pEdge,
                           boolean pActive)
      ```

      Set edge as active/inactive and then draw it.


    - #### drawEdges

      ```
      public void drawEdges(java.awt.Graphics2D g)
      ```


    - #### drawColor

      ```
      public void drawColor(java.awt.Graphics2D g)
      ```


    - #### getAWTShape

      ```
      public java.awt.Shape getAWTShape()
      ```


    - #### getEdgeLine

      ```
      public java.awt.geom.Line2D getEdgeLine(PixelShape.Edge pEdge)
      ```


    - #### isActive

      ```
      public boolean isActive(PixelShape.Edge pEdge)
      ```


    - #### getShape

      ```
      public PixelShape getShape()
      ```


    - #### toString

      ```
      public java.lang.String toString()
      ```

      Overrides:
      :   `toString` in class `java.lang.Object`


    - #### getValidEdges

      ```
      public abstract PixelShape.Edge[] getValidEdges()
      ```


    - #### isValidEdge

      ```
      public abstract boolean isValidEdge(PixelShape.Edge pEdge)
      ```


    - #### getNumEdges

      ```
      public abstract int getNumEdges()
      ```


Skip navigation links


- Overview
- Package
- Class
- Use
- Tree
- Deprecated
- Index
- Help

- Prev Class
- Next Class

- Frames
- No Frames

- All Classes

- Summary:
- Nested |
- Field |
- Constr |
- Method

- Detail:
- Field |
- Constr |
- Method
