# Supplementary material for: Sorting at embryonic boundaries requires high heterotypic interfacial tension
Source: Nat Commun. 2017 Jul 31;8:157. doi: 10.1038/s41467-017-00146-x (PMC5537356; doi:10.1038/s41467-017-00146-x)
Supplement: Supplementary file 2 — Supplementary Software 1 [file 41467_2017_146_MOESM2_ESM.zip › PottsModel/SrcPottsModel/doc/gui/PixelShape.Edge.html]

PixelShape.Edge


JavaScript is disabled on your browser.


Skip navigation links


- Overview
- Package
- Class
- Use
- Tree
- Deprecated
- Index
- Help

- Prev Class
- Next Class

- Frames
- No Frames

- All Classes

- Summary:
- Nested |
- Enum Constants |
- Field |
- Method

- Detail:
- Enum Constants |
- Field |
- Method


gui

## Enum PixelShape.Edge

- java.lang.Object
- - java.lang.Enum<PixelShape.Edge>
  - - gui.PixelShape.Edge

- All Implemented Interfaces:
  :   java.io.Serializable, java.lang.Comparable<PixelShape.Edge>

  Enclosing interface:
  :   PixelShape

  ---

    

  ```
  public static enum PixelShape.Edge
  extends java.lang.Enum<PixelShape.Edge>
  ```

- - ### Enum Constant Summary

    Enum Constants

    | Enum Constant and Description |
    | `bottom` |
    | `bottomLeft` |
    | `bottomRight` |
    | `left` |
    | `right` |
    | `top` |
    | `topLeft` |
    | `topRight` |
  - ### Method Summary

    All Methods Static Methods Concrete Methods

    | Modifier and Type | Method and Description |
    | `static PixelShape.Edge` | `valueOf(java.lang.String name)` Returns the enum constant of this type with the specified name. |
    | `static PixelShape.Edge[]` | `values()` Returns an array containing the constants of this enum type, in the order they are declared. |

    - ### Methods inherited from class java.lang.Enum

      `compareTo, equals, getDeclaringClass, hashCode, name, ordinal, toString, valueOf`
    - ### Methods inherited from class java.lang.Object

      `getClass, notify, notifyAll, wait, wait, wait`

- - ### Enum Constant Detail


    - #### top

      ```
      public static final PixelShape.Edge top
      ```


    - #### right

      ```
      public static final PixelShape.Edge right
      ```


    - #### bottom

      ```
      public static final PixelShape.Edge bottom
      ```


    - #### left

      ```
      public static final PixelShape.Edge left
      ```


    - #### topRight

      ```
      public static final PixelShape.Edge topRight
      ```


    - #### bottomRight

      ```
      public static final PixelShape.Edge bottomRight
      ```


    - #### bottomLeft

      ```
      public static final PixelShape.Edge bottomLeft
      ```


    - #### topLeft

      ```
      public static final PixelShape.Edge topLeft
      ```
  - ### Method Detail


    - #### values

      ```
      public static PixelShape.Edge[] values()
      ```

      Returns an array containing the constants of this enum type, in
      the order they are declared. This method may be used to iterate
      over the constants as follows:

      ```
      for (PixelShape.Edge c : PixelShape.Edge.values())
          System.out.println(c);
      ```

      Returns:
      :   an array containing the constants of this enum type, in the order they are declared


    - #### valueOf

      ```
      public static PixelShape.Edge valueOf(java.lang.String name)
      ```

      Returns the enum constant of this type with the specified name.
      The string must match *exactly* an identifier used to declare an
      enum constant in this type. (Extraneous whitespace characters are
      not permitted.)

      Parameters:
      :   `name` - the name of the enum constant to be returned.

      Returns:
      :   the enum constant with the specified name

      Throws:
      :   `java.lang.IllegalArgumentException` - if this enum type has no constant with the specified name
      :   `java.lang.NullPointerException` - if the argument is null


Skip navigation links


- Overview
- Package
- Class
- Use
- Tree
- Deprecated
- Index
- Help

- Prev Class
- Next Class

- Frames
- No Frames

- All Classes

- Summary:
- Nested |
- Enum Constants |
- Field |
- Method

- Detail:
- Enum Constants |
- Field |
- Method
