# Supplementary material for: Sorting at embryonic boundaries requires high heterotypic interfacial tension
Source: Nat Commun. 2017 Jul 31;8:157. doi: 10.1038/s41467-017-00146-x (PMC5537356; doi:10.1038/s41467-017-00146-x)
Supplement: Supplementary file 2 — Supplementary Software 1 [file 41467_2017_146_MOESM2_ESM.zip › PottsModel/SrcPottsModel/doc/gui/PixelShape.html]

PixelShape


JavaScript is disabled on your browser.


Skip navigation links


- Overview
- Package
- Class
- Use
- Tree
- Deprecated
- Index
- Help

- Prev Class
- Next Class

- Frames
- No Frames

- All Classes

- Summary:
- Nested |
- Field |
- Constr |
- Method

- Detail:
- Field |
- Constr |
- Method


gui

## Interface PixelShape

- All Known Implementing Classes:
  :   Hexagon, Square

  ---

    

  ```
  public interface PixelShape
  ```

  Graphics Managment.

  Author:
  :   eleyine

- - ### Nested Class Summary

    Nested Classes

    | Modifier and Type | Interface and Description |
    | `static class` | `PixelShape.Edge` |
    | `static class` | `PixelShape.Type` |
  - ### Method Summary

    All Methods Instance Methods Abstract Methods

    | Modifier and Type | Method and Description |
    | `java.awt.Shape` | `getAWTShape()` |
    | `java.awt.Point` | `getCenter()` |
    | `Coordinates` | `getCoordinates()` |
    | `java.awt.geom.Line2D` | `getEdge(PixelShape.Edge pEdge)` |
    | `Coordinates` | `getNeighborCoordinates(PixelShape.Edge pEdge)` |
    | `PixelShape.Type` | `getShapeType()` |

- - ### Method Detail


    - #### getCoordinates

      ```
      Coordinates getCoordinates()
      ```


    - #### getNeighborCoordinates

      ```
      Coordinates getNeighborCoordinates(PixelShape.Edge pEdge)
      ```


    - #### getEdge

      ```
      java.awt.geom.Line2D getEdge(PixelShape.Edge pEdge)
      ```


    - #### getCenter

      ```
      java.awt.Point getCenter()
      ```


    - #### getAWTShape

      ```
      java.awt.Shape getAWTShape()
      ```


    - #### getShapeType

      ```
      PixelShape.Type getShapeType()
      ```


Skip navigation links


- Overview
- Package
- Class
- Use
- Tree
- Deprecated
- Index
- Help

- Prev Class
- Next Class

- Frames
- No Frames

- All Classes

- Summary:
- Nested |
- Field |
- Constr |
- Method

- Detail:
- Field |
- Constr |
- Method
