# Supplementary material for: Sorting at embryonic boundaries requires high heterotypic interfacial tension
Source: Nat Commun. 2017 Jul 31;8:157. doi: 10.1038/s41467-017-00146-x (PMC5537356; doi:10.1038/s41467-017-00146-x)
Supplement: Supplementary file 2 — Supplementary Software 1 [file 41467_2017_146_MOESM2_ESM.zip › PottsModel/SrcPottsModel/doc/gui/PottsCanvas.html]

PottsCanvas


JavaScript is disabled on your browser.


Skip navigation links


- Overview
- Package
- Class
- Use
- Tree
- Deprecated
- Index
- Help

- Prev Class
- Next Class

- Frames
- No Frames

- All Classes

- Summary:
- Nested |
- Field |
- Constr |
- Method

- Detail:
- Field |
- Constr |
- Method


gui

## Class PottsCanvas

- java.lang.Object
- - java.awt.Component
  - - java.awt.Container
    - - javax.swing.JComponent
      - - javax.swing.JPanel
        - - gui.PottsCanvas

- All Implemented Interfaces:
  :   java.awt.image.ImageObserver, java.awt.MenuContainer, java.beans.PropertyChangeListener, java.io.Serializable, java.util.EventListener, javax.accessibility.Accessible, Observer

  ---

    

  ```
  public class PottsCanvas
  extends javax.swing.JPanel
  implements java.beans.PropertyChangeListener, Observer
  ```

  Graphical representation of the Potts Model Lattice. Notation: ScreenPixel:
  pixel on the screen PottsPixel: pixel on the spin lattice Screen:
  distance on the screen Potts: distance on the spin lattice

  Author:
  :   Eleyine

  See Also:
  :   Serialized Form

- - ### Nested Class Summary

    - ### Nested classes/interfaces inherited from class javax.swing.JComponent

      `javax.swing.JComponent.AccessibleJComponent`
    - ### Nested classes/interfaces inherited from class java.awt.Component

      `java.awt.Component.BaselineResizeBehavior`
  - ### Field Summary

    Fields

    | Modifier and Type | Field and Description |
    | `static int` | `aNumEdges` |
    | `int` | `aPottsHeight` |
    | `int` | `aPottsWidth` |

    - ### Fields inherited from class javax.swing.JComponent

      `TOOL_TIP_TEXT_KEY, UNDEFINED_CONDITION, WHEN_ANCESTOR_OF_FOCUSED_COMPONENT, WHEN_FOCUSED, WHEN_IN_FOCUSED_WINDOW`
    - ### Fields inherited from class java.awt.Component

      `BOTTOM_ALIGNMENT, CENTER_ALIGNMENT, LEFT_ALIGNMENT, RIGHT_ALIGNMENT, TOP_ALIGNMENT`
    - ### Fields inherited from interface java.awt.image.ImageObserver

      `ABORT, ALLBITS, ERROR, FRAMEBITS, HEIGHT, PROPERTIES, SOMEBITS, WIDTH`
  - ### Constructor Summary

    Constructors

    | Constructor and Description |
    | `PottsCanvas(int pPottsWidth, int pPottsHeight, Constants c)` Constructs a Cartesian grid that can carry (pPottsWidth x pPottsHeight) PottsPixels a.k.a Spins. |
  - ### Method Summary

    All Methods Static Methods Instance Methods Concrete Methods

    | Modifier and Type | Method and Description |
    | `void` | `attachFrame(PottsFrame pFrame)` |
    | `java.awt.Color` | `getColorAt(int x, int y)` Get Potts Model Pixel color at position (x, y). |
    | `static int` | `getEdgeIndex(int x, int y, int e, int n, int m)` Returns edge index |
    | `static int` | `getShapeIndex(int x, int y, int n)` |
    | `static int[]` | `getXY(int i, int n)` |
    | `static int[]` | `getXYE(int i, int n)` |
    | `void` | `initializeMatrix(Lattice pLattice)` |
    | `PixelDisplay` | `locateClickedPixel(java.awt.Point pClickedPoint)` Return pixel clicked. |
    | `static void` | `main(java.lang.String[] args)` |
    | `void` | `paintComponent(java.awt.Graphics g)` |
    | `void` | `propertyChange(java.beans.PropertyChangeEvent evt)` |
    | `void` | `saveSnapshot(java.lang.String filename)` |
    | `void` | `setColorAt(int x, int y, java.awt.Color pixelColor)` Draw pixel at (x,y) with given color. |
    | `void` | `setEdge(int x, int y, PixelShape.Edge pEdge, boolean pActive)` Draw pixel at (x,y) with given color. |
    | `void` | `update(javax.management.Notification pNotification)` Determines what an observer should do upon notification that the observed object has changed. |

    - ### Methods inherited from class javax.swing.JPanel

      `getAccessibleContext, getUI, getUIClassID, setUI, updateUI`
    - ### Methods inherited from class javax.swing.JComponent

      `addAncestorListener, addNotify, addVetoableChangeListener, computeVisibleRect, contains, createToolTip, disable, enable, firePropertyChange, firePropertyChange, firePropertyChange, getActionForKeyStroke, getActionMap, getAlignmentX, getAlignmentY, getAncestorListeners, getAutoscrolls, getBaseline, getBaselineResizeBehavior, getBorder, getBounds, getClientProperty, getComponentPopupMenu, getConditionForKeyStroke, getDebugGraphicsOptions, getDefaultLocale, getFontMetrics, getGraphics, getHeight, getInheritsPopupMenu, getInputMap, getInputMap, getInputVerifier, getInsets, getInsets, getListeners, getLocation, getMaximumSize, getMinimumSize, getNextFocusableComponent, getPopupLocation, getPreferredSize, getRegisteredKeyStrokes, getRootPane, getSize, getToolTipLocation, getToolTipText, getToolTipText, getTopLevelAncestor, getTransferHandler, getVerifyInputWhenFocusTarget, getVetoableChangeListeners, getVisibleRect, getWidth, getX, getY, grabFocus, hide, isDoubleBuffered, isLightweightComponent, isManagingFocus, isOpaque, isOptimizedDrawingEnabled, isPaintingForPrint, isPaintingTile, isRequestFocusEnabled, isValidateRoot, paint, paintImmediately, paintImmediately, print, printAll, putClientProperty, registerKeyboardAction, registerKeyboardAction, removeAncestorListener, removeNotify, removeVetoableChangeListener, repaint, repaint, requestDefaultFocus, requestFocus, requestFocus, requestFocusInWindow, resetKeyboardActions, reshape, revalidate, scrollRectToVisible, setActionMap, setAlignmentX, setAlignmentY, setAutoscrolls, setBackground, setBorder, setComponentPopupMenu, setDebugGraphicsOptions, setDefaultLocale, setDoubleBuffered, setEnabled, setFocusTraversalKeys, setFont, setForeground, setInheritsPopupMenu, setInputMap, setInputVerifier, setMaximumSize, setMinimumSize, setNextFocusableComponent, setOpaque, setPreferredSize, setRequestFocusEnabled, setToolTipText, setTransferHandler, setVerifyInputWhenFocusTarget, setVisible, unregisterKeyboardAction, update`
    - ### Methods inherited from class java.awt.Container

      `add, add, add, add, add, addContainerListener, addPropertyChangeListener, addPropertyChangeListener, applyComponentOrientation, areFocusTraversalKeysSet, countComponents, deliverEvent, doLayout, findComponentAt, findComponentAt, getComponent, getComponentAt, getComponentAt, getComponentCount, getComponents, getComponentZOrder, getContainerListeners, getFocusTraversalKeys, getFocusTraversalPolicy, getLayout, getMousePosition, insets, invalidate, isAncestorOf, isFocusCycleRoot, isFocusCycleRoot, isFocusTraversalPolicyProvider, isFocusTraversalPolicySet, layout, list, list, locate, minimumSize, paintComponents, preferredSize, printComponents, remove, remove, removeAll, removeContainerListener, setComponentZOrder, setFocusCycleRoot, setFocusTraversalPolicy, setFocusTraversalPolicyProvider, setLayout, transferFocusDownCycle, validate`
    - ### Methods inherited from class java.awt.Component

      `action, add, addComponentListener, addFocusListener, addHierarchyBoundsListener, addHierarchyListener, addInputMethodListener, addKeyListener, addMouseListener, addMouseMotionListener, addMouseWheelListener, bounds, checkImage, checkImage, contains, createImage, createImage, createVolatileImage, createVolatileImage, dispatchEvent, enable, enableInputMethods, firePropertyChange, firePropertyChange, firePropertyChange, firePropertyChange, firePropertyChange, getBackground, getBounds, getColorModel, getComponentListeners, getComponentOrientation, getCursor, getDropTarget, getFocusCycleRootAncestor, getFocusListeners, getFocusTraversalKeysEnabled, getFont, getForeground, getGraphicsConfiguration, getHierarchyBoundsListeners, getHierarchyListeners, getIgnoreRepaint, getInputContext, getInputMethodListeners, getInputMethodRequests, getKeyListeners, getLocale, getLocation, getLocationOnScreen, getMouseListeners, getMouseMotionListeners, getMousePosition, getMouseWheelListeners, getName, getParent, getPeer, getPropertyChangeListeners, getPropertyChangeListeners, getSize, getToolkit, getTreeLock, gotFocus, handleEvent, hasFocus, imageUpdate, inside, isBackgroundSet, isCursorSet, isDisplayable, isEnabled, isFocusable, isFocusOwner, isFocusTraversable, isFontSet, isForegroundSet, isLightweight, isMaximumSizeSet, isMinimumSizeSet, isPreferredSizeSet, isShowing, isValid, isVisible, keyDown, keyUp, list, list, list, location, lostFocus, mouseDown, mouseDrag, mouseEnter, mouseExit, mouseMove, mouseUp, move, nextFocus, paintAll, postEvent, prepareImage, prepareImage, remove, removeComponentListener, removeFocusListener, removeHierarchyBoundsListener, removeHierarchyListener, removeInputMethodListener, removeKeyListener, removeMouseListener, removeMouseMotionListener, removeMouseWheelListener, removePropertyChangeListener, removePropertyChangeListener, repaint, repaint, repaint, resize, resize, setBounds, setBounds, setComponentOrientation, setCursor, setDropTarget, setFocusable, setFocusTraversalKeysEnabled, setIgnoreRepaint, setLocale, setLocation, setLocation, setName, setSize, setSize, show, show, size, toString, transferFocus, transferFocusBackward, transferFocusUpCycle`
    - ### Methods inherited from class java.lang.Object

      `equals, getClass, hashCode, notify, notifyAll, wait, wait, wait`

- - ### Field Detail


    - #### aPottsWidth

      ```
      public final int aPottsWidth
      ```


    - #### aPottsHeight

      ```
      public final int aPottsHeight
      ```


    - #### aNumEdges

      ```
      public static final int aNumEdges
      ```
  - ### Constructor Detail


    - #### PottsCanvas

      ```
      public PottsCanvas(int pPottsWidth,
                         int pPottsHeight,
                         Constants c)
      ```

      Constructs a Cartesian grid that can carry (pPottsWidth x pPottsHeight)
      PottsPixels a.k.a Spins. The grid itself has default pixel dimensions.

      Parameters:
      :   `pPottsWidth` - the width of the spin lattice
      :   `pPottsHeight` - the height of the spin lattice.
  - ### Method Detail


    - #### attachFrame

      ```
      public void attachFrame(PottsFrame pFrame)
      ```


    - #### initializeMatrix

      ```
      public void initializeMatrix(Lattice pLattice)
      ```


    - #### setColorAt

      ```
      public void setColorAt(int x,
                             int y,
                             java.awt.Color pixelColor)
      ```

      Draw pixel at (x,y) with given color.

      Parameters:
      :   `x` - x coordinate of the PottsPixel
      :   `y` - y coordinate of the PottsPixel
      :   `pixelColor` -


    - #### setEdge

      ```
      public void setEdge(int x,
                          int y,
                          PixelShape.Edge pEdge,
                          boolean pActive)
      ```

      Draw pixel at (x,y) with given color.

      Parameters:
      :   `x` - x coordinate of the PottsPixel
      :   `y` - y coordinate of the PottsPixel
      :   `pixelColor` -


    - #### getColorAt

      ```
      public java.awt.Color getColorAt(int x,
                                       int y)
      ```

      Get Potts Model Pixel color at position (x, y).

      Parameters:
      :   `x` - x coordinate of the PottsPixel
      :   `y` - y coordinate of the PottsPixel

      Returns:
      :   the Color of the PottsPixel at position (x,y).


    - #### locateClickedPixel

      ```
      public PixelDisplay locateClickedPixel(java.awt.Point pClickedPoint)
      ```

      Return pixel clicked. Different strategies based on pixel shape.

      Parameters:
      :   `pMouseX` -
      :   `pMouseY` -

      Returns:


    - #### paintComponent

      ```
      public void paintComponent(java.awt.Graphics g)
      ```

      Overrides:
      :   `paintComponent` in class `javax.swing.JComponent`


    - #### propertyChange

      ```
      public void propertyChange(java.beans.PropertyChangeEvent evt)
      ```

      Specified by:
      :   `propertyChange` in interface `java.beans.PropertyChangeListener`


    - #### update

      ```
      public void update(javax.management.Notification pNotification)
      ```

      Description copied from interface: `Observer`

      Determines what an observer should do upon notification that the observed object has changed.

      Specified by:
      :   `update` in interface `Observer`

      Parameters:
      :   `pNotification` - : Notification passed by the object being observed.


    - #### saveSnapshot

      ```
      public void saveSnapshot(java.lang.String filename)
      ```


    - #### getEdgeIndex

      ```
      public static int getEdgeIndex(int x,
                                     int y,
                                     int e,
                                     int n,
                                     int m)
      ```

      Returns edge index

      Parameters:
      :   `x` -
      :   `y` -
      :   `e` -
      :   `n` -
      :   `m` -

      Returns:


    - #### getShapeIndex

      ```
      public static int getShapeIndex(int x,
                                      int y,
                                      int n)
      ```


    - #### getXYE

      ```
      public static int[] getXYE(int i,
                                 int n)
      ```


    - #### getXY

      ```
      public static int[] getXY(int i,
                                int n)
      ```


    - #### main

      ```
      public static void main(java.lang.String[] args)
      ```


Skip navigation links


- Overview
- Package
- Class
- Use
- Tree
- Deprecated
- Index
- Help

- Prev Class
- Next Class

- Frames
- No Frames

- All Classes

- Summary:
- Nested |
- Field |
- Constr |
- Method

- Detail:
- Field |
- Constr |
- Method
