# Supplementary material for: Sorting at embryonic boundaries requires high heterotypic interfacial tension
Source: Nat Commun. 2017 Jul 31;8:157. doi: 10.1038/s41467-017-00146-x (PMC5537356; doi:10.1038/s41467-017-00146-x)
Supplement: Supplementary file 2 — Supplementary Software 1 [file 41467_2017_146_MOESM2_ESM.zip › PottsModel/SrcPottsModel/doc/gui/PottsFrame.html]

PottsFrame


JavaScript is disabled on your browser.


Skip navigation links


- Overview
- Package
- Class
- Use
- Tree
- Deprecated
- Index
- Help

- Prev Class
- Next Class

- Frames
- No Frames

- All Classes

- Summary:
- Nested |
- Field |
- Constr |
- Method

- Detail:
- Field |
- Constr |
- Method


gui

## Class PottsFrame

- java.lang.Object
- - java.awt.Component
  - - java.awt.Container
    - - java.awt.Window
      - - java.awt.Frame
        - - javax.swing.JFrame
          - - gui.PottsFrame

- All Implemented Interfaces:
  :   java.awt.event.ActionListener, java.awt.image.ImageObserver, java.awt.MenuContainer, java.io.Serializable, java.util.EventListener, javax.accessibility.Accessible, javax.swing.RootPaneContainer, javax.swing.WindowConstants, Observer

  ---

    

  ```
  public class PottsFrame
  extends javax.swing.JFrame
  implements Observer, java.awt.event.ActionListener
  ```

  See Also:
  :   Serialized Form

- - ### Nested Class Summary

    Nested Classes

    | Modifier and Type | Class and Description |
    | `static class` | `PottsFrame.Action` |

    - ### Nested classes/interfaces inherited from class java.awt.Window

      `java.awt.Window.Type`
    - ### Nested classes/interfaces inherited from class java.awt.Component

      `java.awt.Component.BaselineResizeBehavior`
  - ### Field Summary

    Fields

    | Modifier and Type | Field and Description |
    | `java.util.concurrent.atomic.AtomicBoolean` | `aAutomaticMode` |
    | `java.util.concurrent.atomic.AtomicBoolean` | `aMCSAndPause` |
    | `java.util.concurrent.atomic.AtomicBoolean` | `aPaused` |
    | `java.util.concurrent.atomic.AtomicBoolean` | `aSwapAndPause` |

    - ### Fields inherited from class javax.swing.JFrame

      `EXIT_ON_CLOSE`
    - ### Fields inherited from class java.awt.Frame

      `CROSSHAIR_CURSOR, DEFAULT_CURSOR, E_RESIZE_CURSOR, HAND_CURSOR, ICONIFIED, MAXIMIZED_BOTH, MAXIMIZED_HORIZ, MAXIMIZED_VERT, MOVE_CURSOR, N_RESIZE_CURSOR, NE_RESIZE_CURSOR, NORMAL, NW_RESIZE_CURSOR, S_RESIZE_CURSOR, SE_RESIZE_CURSOR, SW_RESIZE_CURSOR, TEXT_CURSOR, W_RESIZE_CURSOR, WAIT_CURSOR`
    - ### Fields inherited from class java.awt.Component

      `BOTTOM_ALIGNMENT, CENTER_ALIGNMENT, LEFT_ALIGNMENT, RIGHT_ALIGNMENT, TOP_ALIGNMENT`
    - ### Fields inherited from interface javax.swing.WindowConstants

      `DISPOSE_ON_CLOSE, DO_NOTHING_ON_CLOSE, HIDE_ON_CLOSE`
    - ### Fields inherited from interface java.awt.image.ImageObserver

      `ABORT, ALLBITS, ERROR, FRAMEBITS, HEIGHT, PROPERTIES, SOMEBITS, WIDTH`
  - ### Constructor Summary

    Constructors

    | Constructor and Description |
    | `PottsFrame(PottsEngine pEngine)` |
  - ### Method Summary

    All Methods Instance Methods Concrete Methods

    | Modifier and Type | Method and Description |
    | `void` | `actionPerformed(java.awt.event.ActionEvent e)` |
    | `void` | `addCanvas(PottsCanvas pCanvas)` |
    | `void` | `addTabToSidePane(java.lang.String name, javax.swing.JPanel p)` |
    | `void` | `displayCellInformation(int pX, int pY)` |
    | `void` | `displayPixelInformation(int pX, int pY)` |
    | `void` | `reset()` |
    | `void` | `setAutomaticMode(boolean pOn)` |
    | `void` | `showInfo()` |
    | `void` | `showStats()` |
    | `void` | `update(javax.management.Notification pNotification)` Determines what an observer should do upon notification that the observed object has changed. |
    | `void` | `updateStatisticsManager(StatisticsManager pStatsManager)` |

    - ### Methods inherited from class javax.swing.JFrame

      `getAccessibleContext, getContentPane, getDefaultCloseOperation, getGlassPane, getGraphics, getJMenuBar, getLayeredPane, getRootPane, getTransferHandler, isDefaultLookAndFeelDecorated, remove, repaint, setContentPane, setDefaultCloseOperation, setDefaultLookAndFeelDecorated, setGlassPane, setIconImage, setJMenuBar, setLayeredPane, setLayout, setTransferHandler, update`
    - ### Methods inherited from class java.awt.Frame

      `addNotify, getCursorType, getExtendedState, getFrames, getIconImage, getMaximizedBounds, getMenuBar, getState, getTitle, isResizable, isUndecorated, remove, removeNotify, setBackground, setCursor, setExtendedState, setMaximizedBounds, setMenuBar, setOpacity, setResizable, setShape, setState, setTitle, setUndecorated`
    - ### Methods inherited from class java.awt.Window

      `addPropertyChangeListener, addPropertyChangeListener, addWindowFocusListener, addWindowListener, addWindowStateListener, applyResourceBundle, applyResourceBundle, createBufferStrategy, createBufferStrategy, dispose, getBackground, getBufferStrategy, getFocusableWindowState, getFocusCycleRootAncestor, getFocusOwner, getFocusTraversalKeys, getIconImages, getInputContext, getListeners, getLocale, getModalExclusionType, getMostRecentFocusOwner, getOpacity, getOwnedWindows, getOwner, getOwnerlessWindows, getShape, getToolkit, getType, getWarningString, getWindowFocusListeners, getWindowListeners, getWindows, getWindowStateListeners, hide, isActive, isAlwaysOnTop, isAlwaysOnTopSupported, isAutoRequestFocus, isFocusableWindow, isFocusCycleRoot, isFocused, isLocationByPlatform, isOpaque, isShowing, isValidateRoot, pack, paint, postEvent, removeWindowFocusListener, removeWindowListener, removeWindowStateListener, reshape, setAlwaysOnTop, setAutoRequestFocus, setBounds, setBounds, setCursor, setFocusableWindowState, setFocusCycleRoot, setIconImages, setLocation, setLocation, setLocationByPlatform, setLocationRelativeTo, setMinimumSize, setModalExclusionType, setSize, setSize, setType, setVisible, show, toBack, toFront`
    - ### Methods inherited from class java.awt.Container

      `add, add, add, add, add, addContainerListener, applyComponentOrientation, areFocusTraversalKeysSet, countComponents, deliverEvent, doLayout, findComponentAt, findComponentAt, getAlignmentX, getAlignmentY, getComponent, getComponentAt, getComponentAt, getComponentCount, getComponents, getComponentZOrder, getContainerListeners, getFocusTraversalPolicy, getInsets, getLayout, getMaximumSize, getMinimumSize, getMousePosition, getPreferredSize, insets, invalidate, isAncestorOf, isFocusCycleRoot, isFocusTraversalPolicyProvider, isFocusTraversalPolicySet, layout, list, list, locate, minimumSize, paintComponents, preferredSize, print, printComponents, remove, removeAll, removeContainerListener, setComponentZOrder, setFocusTraversalKeys, setFocusTraversalPolicy, setFocusTraversalPolicyProvider, setFont, transferFocusDownCycle, validate`
    - ### Methods inherited from class java.awt.Component

      `action, add, addComponentListener, addFocusListener, addHierarchyBoundsListener, addHierarchyListener, addInputMethodListener, addKeyListener, addMouseListener, addMouseMotionListener, addMouseWheelListener, bounds, checkImage, checkImage, contains, contains, createImage, createImage, createVolatileImage, createVolatileImage, disable, dispatchEvent, enable, enable, enableInputMethods, firePropertyChange, firePropertyChange, firePropertyChange, firePropertyChange, firePropertyChange, firePropertyChange, getBaseline, getBaselineResizeBehavior, getBounds, getBounds, getColorModel, getComponentListeners, getComponentOrientation, getCursor, getDropTarget, getFocusListeners, getFocusTraversalKeysEnabled, getFont, getFontMetrics, getForeground, getGraphicsConfiguration, getHeight, getHierarchyBoundsListeners, getHierarchyListeners, getIgnoreRepaint, getInputMethodListeners, getInputMethodRequests, getKeyListeners, getLocation, getLocation, getLocationOnScreen, getMouseListeners, getMouseMotionListeners, getMousePosition, getMouseWheelListeners, getName, getParent, getPeer, getPropertyChangeListeners, getPropertyChangeListeners, getSize, getSize, getTreeLock, getWidth, getX, getY, gotFocus, handleEvent, hasFocus, imageUpdate, inside, isBackgroundSet, isCursorSet, isDisplayable, isDoubleBuffered, isEnabled, isFocusable, isFocusOwner, isFocusTraversable, isFontSet, isForegroundSet, isLightweight, isMaximumSizeSet, isMinimumSizeSet, isPreferredSizeSet, isValid, isVisible, keyDown, keyUp, list, list, list, location, lostFocus, mouseDown, mouseDrag, mouseEnter, mouseExit, mouseMove, mouseUp, move, nextFocus, paintAll, prepareImage, prepareImage, printAll, removeComponentListener, removeFocusListener, removeHierarchyBoundsListener, removeHierarchyListener, removeInputMethodListener, removeKeyListener, removeMouseListener, removeMouseMotionListener, removeMouseWheelListener, removePropertyChangeListener, removePropertyChangeListener, repaint, repaint, repaint, requestFocus, requestFocusInWindow, resize, resize, revalidate, setComponentOrientation, setDropTarget, setEnabled, setFocusable, setFocusTraversalKeysEnabled, setForeground, setIgnoreRepaint, setLocale, setMaximumSize, setName, setPreferredSize, show, size, toString, transferFocus, transferFocusBackward, transferFocusUpCycle`
    - ### Methods inherited from class java.lang.Object

      `equals, getClass, hashCode, notify, notifyAll, wait, wait, wait`
    - ### Methods inherited from interface java.awt.MenuContainer

      `getFont, postEvent`

- - ### Field Detail


    - #### aPaused

      ```
      public java.util.concurrent.atomic.AtomicBoolean aPaused
      ```


    - #### aSwapAndPause

      ```
      public java.util.concurrent.atomic.AtomicBoolean aSwapAndPause
      ```


    - #### aMCSAndPause

      ```
      public java.util.concurrent.atomic.AtomicBoolean aMCSAndPause
      ```


    - #### aAutomaticMode

      ```
      public java.util.concurrent.atomic.AtomicBoolean aAutomaticMode
      ```
  - ### Constructor Detail


    - #### PottsFrame

      ```
      public PottsFrame(PottsEngine pEngine)
      ```
  - ### Method Detail


    - #### reset

      ```
      public void reset()
      ```


    - #### showInfo

      ```
      public void showInfo()
      ```


    - #### showStats

      ```
      public void showStats()
      ```


    - #### addTabToSidePane

      ```
      public void addTabToSidePane(java.lang.String name,
                                   javax.swing.JPanel p)
      ```


    - #### addCanvas

      ```
      public void addCanvas(PottsCanvas pCanvas)
      ```


    - #### update

      ```
      public void update(javax.management.Notification pNotification)
      ```

      Description copied from interface: `Observer`

      Determines what an observer should do upon notification that the observed object has changed.

      Specified by:
      :   `update` in interface `Observer`

      Parameters:
      :   `pNotification` - : Notification passed by the object being observed.


    - #### setAutomaticMode

      ```
      public void setAutomaticMode(boolean pOn)
      ```


    - #### displayPixelInformation

      ```
      public void displayPixelInformation(int pX,
                                          int pY)
      ```


    - #### displayCellInformation

      ```
      public void displayCellInformation(int pX,
                                         int pY)
      ```


    - #### actionPerformed

      ```
      public void actionPerformed(java.awt.event.ActionEvent e)
      ```

      Specified by:
      :   `actionPerformed` in interface `java.awt.event.ActionListener`


    - #### updateStatisticsManager

      ```
      public void updateStatisticsManager(StatisticsManager pStatsManager)
      ```


Skip navigation links


- Overview
- Package
- Class
- Use
- Tree
- Deprecated
- Index
- Help

- Prev Class
- Next Class

- Frames
- No Frames

- All Classes

- Summary:
- Nested |
- Field |
- Constr |
- Method

- Detail:
- Field |
- Constr |
- Method
