# Supplementary material for: Sorting at embryonic boundaries requires high heterotypic interfacial tension
Source: Nat Commun. 2017 Jul 31;8:157. doi: 10.1038/s41467-017-00146-x (PMC5537356; doi:10.1038/s41467-017-00146-x)
Supplement: Supplementary file 2 — Supplementary Software 1 [file 41467_2017_146_MOESM2_ESM.zip › PottsModel/SrcPottsModel/doc/gui/SnapshotManager.html]

SnapshotManager


JavaScript is disabled on your browser.


Skip navigation links


- Overview
- Package
- Class
- Use
- Tree
- Deprecated
- Index
- Help

- Prev Class
- Next Class

- Frames
- No Frames

- All Classes

- Summary:
- Nested |
- Field |
- Constr |
- Method

- Detail:
- Field |
- Constr |
- Method


gui

## Class SnapshotManager

- java.lang.Object
- - gui.SnapshotManager

- All Implemented Interfaces:
  :   Observer

  ---

    

  ```
  public class SnapshotManager
  extends java.lang.Object
  implements Observer
  ```

  Graphical representation of the Potts Model Lattice. Notation: ScreenPixel:
  pixel on the screen PottsPixel: pixel on the spin lattice Screen:
  distance on the screen Potts: distance on the spin lattice

  Author:
  :   Eleyine

- - ### Field Summary

    Fields

    | Modifier and Type | Field and Description |
    | `static int` | `aNumEdges` |
    | `int` | `aPottsHeight` |
    | `int` | `aPottsWidth` |
  - ### Constructor Summary

    Constructors

    | Constructor and Description |
    | `SnapshotManager(int width, int height, Constants c)` Constructs a Cartesian grid that can carry (pPottsWidth x pPottsHeight) PottsPixels a.k.a Spins. |
  - ### Method Summary

    All Methods Instance Methods Concrete Methods

    | Modifier and Type | Method and Description |
    | `void` | `initializeMatrix(Lattice pLattice)` |
    | `void` | `repaint(Lattice pLattice)` |
    | `void` | `saveSnapshot(Lattice pLattice, java.lang.String filename)` |
    | `void` | `update(javax.management.Notification pNotification)` Determines what an observer should do upon notification that the observed object has changed. |

    - ### Methods inherited from class java.lang.Object

      `equals, getClass, hashCode, notify, notifyAll, toString, wait, wait, wait`

- - ### Field Detail


    - #### aPottsWidth

      ```
      public final int aPottsWidth
      ```


    - #### aPottsHeight

      ```
      public final int aPottsHeight
      ```


    - #### aNumEdges

      ```
      public static final int aNumEdges
      ```
  - ### Constructor Detail


    - #### SnapshotManager

      ```
      public SnapshotManager(int width,
                             int height,
                             Constants c)
      ```

      Constructs a Cartesian grid that can carry (pPottsWidth x pPottsHeight)
      PottsPixels a.k.a Spins. The grid itself has default pixel dimensions.

      Parameters:
      :   `pPottsWidth` - the width of the spin lattice
      :   `pPottsHeight` - the height of the spin lattice.
  - ### Method Detail


    - #### initializeMatrix

      ```
      public void initializeMatrix(Lattice pLattice)
      ```


    - #### repaint

      ```
      public void repaint(Lattice pLattice)
      ```


    - #### update

      ```
      public void update(javax.management.Notification pNotification)
      ```

      Description copied from interface: `Observer`

      Determines what an observer should do upon notification that the observed object has changed.

      Specified by:
      :   `update` in interface `Observer`

      Parameters:
      :   `pNotification` - : Notification passed by the object being observed.


    - #### saveSnapshot

      ```
      public void saveSnapshot(Lattice pLattice,
                               java.lang.String filename)
      ```


Skip navigation links


- Overview
- Package
- Class
- Use
- Tree
- Deprecated
- Index
- Help

- Prev Class
- Next Class

- Frames
- No Frames

- All Classes

- Summary:
- Nested |
- Field |
- Constr |
- Method

- Detail:
- Field |
- Constr |
- Method
