# Supplementary material for: Sorting at embryonic boundaries requires high heterotypic interfacial tension
Source: Nat Commun. 2017 Jul 31;8:157. doi: 10.1038/s41467-017-00146-x (PMC5537356; doi:10.1038/s41467-017-00146-x)
Supplement: Supplementary file 2 — Supplementary Software 1 [file 41467_2017_146_MOESM2_ESM.zip › PottsModel/SrcPottsModel/doc/gui/SquarePixelDisplay.html]

SquarePixelDisplay


JavaScript is disabled on your browser.


Skip navigation links


- Overview
- Package
- Class
- Use
- Tree
- Deprecated
- Index
- Help

- Prev Class
- Next Class

- Frames
- No Frames

- All Classes

- Summary:
- Nested |
- Field |
- Constr |
- Method

- Detail:
- Field |
- Constr |
- Method


gui

## Class SquarePixelDisplay

- java.lang.Object
- - gui.PixelDisplay
  - - gui.SquarePixelDisplay

- ---

    

  ```
  public class SquarePixelDisplay
  extends PixelDisplay
  ```

- - ### Field Summary

    - ### Fields inherited from class gui.PixelDisplay

      `aColor`
  - ### Constructor Summary

    Constructors

    | Constructor and Description |
    | `SquarePixelDisplay(int pX, int pY, int pUnitSize)` |
    | `SquarePixelDisplay(int pX, int pY, int pUnitSize, java.awt.Color pColor)` |
  - ### Method Summary

    All Methods Instance Methods Concrete Methods

    | Modifier and Type | Method and Description |
    | `int` | `getEdgeIndex(PixelShape.Edge pEdge)` |
    | `int` | `getNumEdges()` |
    | `PixelShape.Edge[]` | `getValidEdges()` |
    | `boolean` | `isValidEdge(PixelShape.Edge pEdge)` |

    - ### Methods inherited from class gui.PixelDisplay

      `drawColor, drawEdge, drawEdges, getAWTShape, getEdgeLine, getShape, isActive, toString`
    - ### Methods inherited from class java.lang.Object

      `equals, getClass, hashCode, notify, notifyAll, wait, wait, wait`

- - ### Constructor Detail


    - #### SquarePixelDisplay

      ```
      public SquarePixelDisplay(int pX,
                                int pY,
                                int pUnitSize,
                                java.awt.Color pColor)
      ```


    - #### SquarePixelDisplay

      ```
      public SquarePixelDisplay(int pX,
                                int pY,
                                int pUnitSize)
      ```
  - ### Method Detail


    - #### getEdgeIndex

      ```
      public int getEdgeIndex(PixelShape.Edge pEdge)
      ```

      See Also:
      :   `getEdge`


    - #### isValidEdge

      ```
      public boolean isValidEdge(PixelShape.Edge pEdge)
      ```

      Specified by:
      :   `isValidEdge` in class `PixelDisplay`


    - #### getValidEdges

      ```
      public PixelShape.Edge[] getValidEdges()
      ```

      Specified by:
      :   `getValidEdges` in class `PixelDisplay`


    - #### getNumEdges

      ```
      public int getNumEdges()
      ```

      Specified by:
      :   `getNumEdges` in class `PixelDisplay`


Skip navigation links


- Overview
- Package
- Class
- Use
- Tree
- Deprecated
- Index
- Help

- Prev Class
- Next Class

- Frames
- No Frames

- All Classes

- Summary:
- Nested |
- Field |
- Constr |
- Method

- Detail:
- Field |
- Constr |
- Method
