# Supplementary material for: Sorting at embryonic boundaries requires high heterotypic interfacial tension
Source: Nat Commun. 2017 Jul 31;8:157. doi: 10.1038/s41467-017-00146-x (PMC5537356; doi:10.1038/s41467-017-00146-x)
Supplement: Supplementary file 3 — Supplementary Information [file 41467_2017_146_MOESM3_ESM.pdf]

File Name: Peer Review File
Description:

File Name: Supplementary Information
Description: Supplementary Figures, Supplementary Tables, Supplementary Methods, Supplementary References.

File Name: Supplementary Software 1
Description: Java code of the Modified Potts Model

Supplementary Information

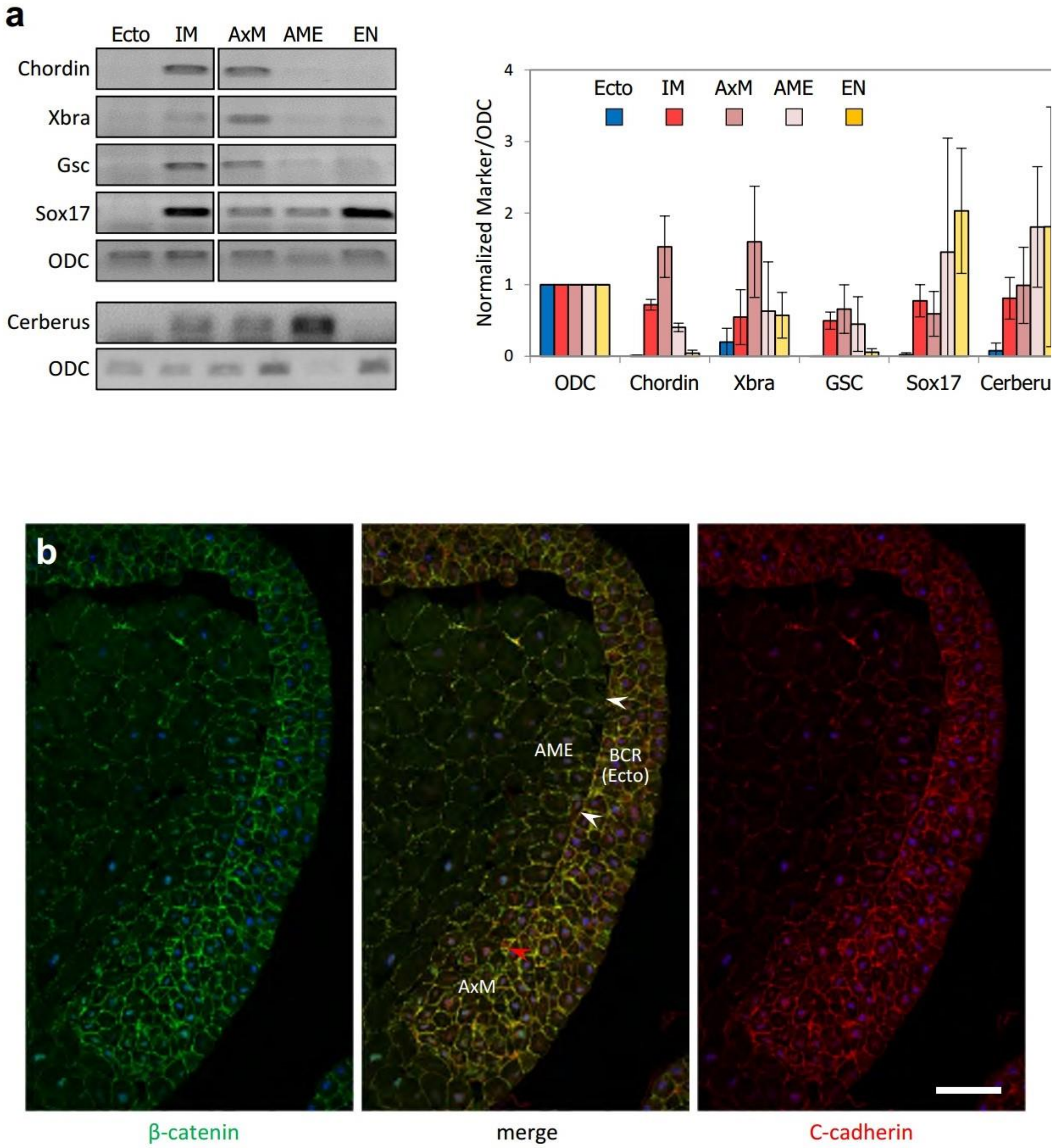

Supplementary Figure 1 | Characterization of induced mesoderm. (**a**) Characterization of dissected tissues by RT-PCR. Cerberus is a marker for anterior mesendoderm, Gsc, and Chordin for prechordal mesoderm, Xbra for chordal mesoderm, Sox17 is enriched in the endoderm, but also expressed at lower levels in the mesoderm. Ubiquitously expressed ODC was used as a standard. Quantification is from 3 independent experiments. Error bars are s.d. (**b**) C-cadherin (red) and β-catenin (green) immunostaining in the gastrula embryo. Nuclei were stained with Hoechst (blue). BCR (Ecto), ectoderm blastocoel roof; AME, anterior mesendoderm; AxM, axial mesoderm. Arrowheads point to the ectoderm-mesoderm boundary. The axial mesoderm is still mostly at the blastopore lip, but starts to participate to the nascent boundary (red arrowhead) and already displays separation activity from ectoderm in vitro [1].

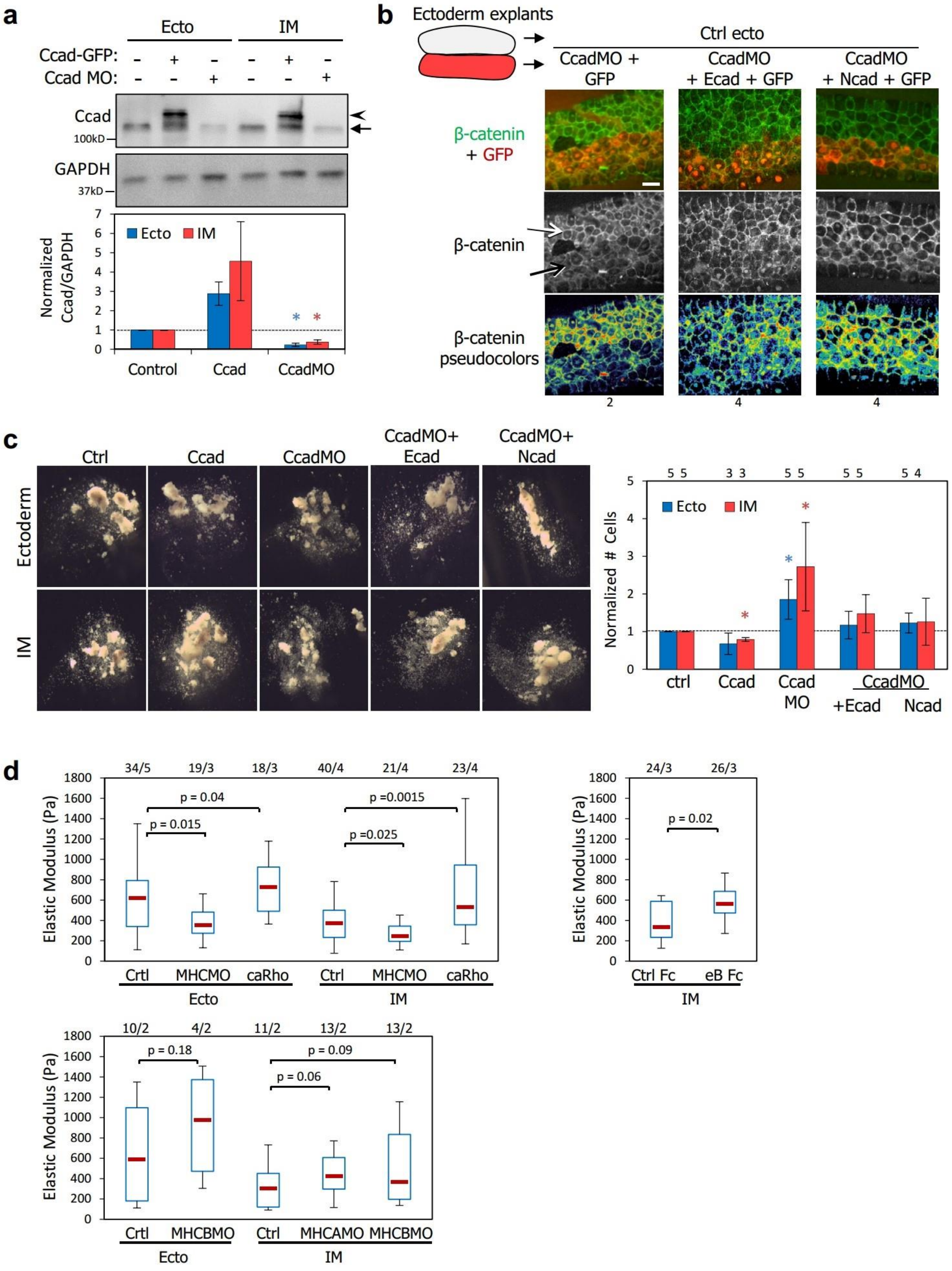
a
Ecto
IM
Ccad-GFP:
Ccad MO:
Ccad
100kD
GAPDH
37kD
Normalized Ccad/GAPDH
Ecto
IM
Control
Ccad
CcadMO
b
Ectoderm explants
Ctrl ecto
CcadMO + GFP
CcadMO + Ecad + GFP
CcadMO + Ncad + GFP
β-catenin + GFP
β-catenin
β-catenin pseudocolors
2
4
4
c
Ctrl
Ccad
CcadMO
CcadMO+ Ecad
CcadMO+ Ncad
Ectoderm
IM
Normalized # Cells
5 5
3 3
5 5
5 5
5 4
Ecto
IM
ctrl
Ccad
Ccad MO
CcadMO
+Ecad
Ncad
d
34/5
19/3
18/3
40/4
21/4
23/4
p = 0.04
p = 0.015
p =0.0015
p =0.025
Elastic Modulus (Pa)
Crtl
MHCMO
caRho
Ctrl
MHCMO
caRho
Ecto
IM
24/3
26/3
p = 0.02
Ctrl Fc
eB Fc
IM
10/2
4/2
11/2
13/2
13/2
p = 0.18
p = 0.09
p = 0.06
Crtl
MHCBMO
Ctrl
MHCAMO
MHCBMO
Ecto
IM

Supplementary Figure 2 | Experimental manipulation adhesion and actomyosin contractility. (**a**) Cadherin overexpression and depletion. Comparison by immunoblot of C-cadherin protein levels in control tissues and tissues from embryos injected with C-cadherin-GFP mRNA or C-Cadherin MO. Arrow, endogenous C-cadherin; arrowhead: Ccadherin-GFP. Quantification was performed from three independent experiments. Individual comparisons were done using Games-Howell test (* P<0.05) after a significant One-way Welch ANOVA (P<0.0001; unequal variances). Error bars are s.d. (**b**) Validation of cadherin depletion and replacement. C-cadherin was depleted by MO injection and replaced with E- or N-cadherin by co-injection of the corresponding mRNA. GFP mRNA was co-injected as a tracer. Manipulated ectoderm explants were juxtaposed to wild type non-injected ectoderm explants for direct comparison of β-catenin membrane immunostaining, used as a general marker for cadherin-based adhesive structures. Pseudocolors were added to help visualizing the decrease of β-catenin signal caused by CadMO and the rescue by expression of E- or N-cadherin. Numbers of samples indicated at the bottom. Scale bar, 30µm. (**c**) Effect of C-cadherin overexpression, depletion, and replacement on ectoderm and mesoderm cohesion. The same conditions as in (b) were tested for resistance to dissociation. Graph shows mean values and error bars are s.d. Number of experiments is given above the graph. Individual comparisons were done using Games-Howell test (* P<0.05) after a significant one-way Welch ANOVA P<0.0001; for unequal variances). (**d**) Manipulation of cortical contractility. Top left panel: Effect of MHC2A+B depletion (MHCMOs) and Rho activation (caRhoA mRNA) on the elastic modulus of ectoderm and induced mesoderm cells. Data are from 5 independent experiments. Right: Effect of Eph stimulation by soluble ephrinB3-Fc ligand. Lower left: Single myosin isoform depletion (MHC2A or MHC2B MO) did not cause a decrease in elastic modulus, suggesting that each isoform is able to compensate for the loss of the other. Box plots as in main Fig.2e. The number of cells tested/independent experiment is above the graph. Individual comparisons were done using Tukey's Hs.d test after a significant One-way ANOVA (P=5.7e-08).

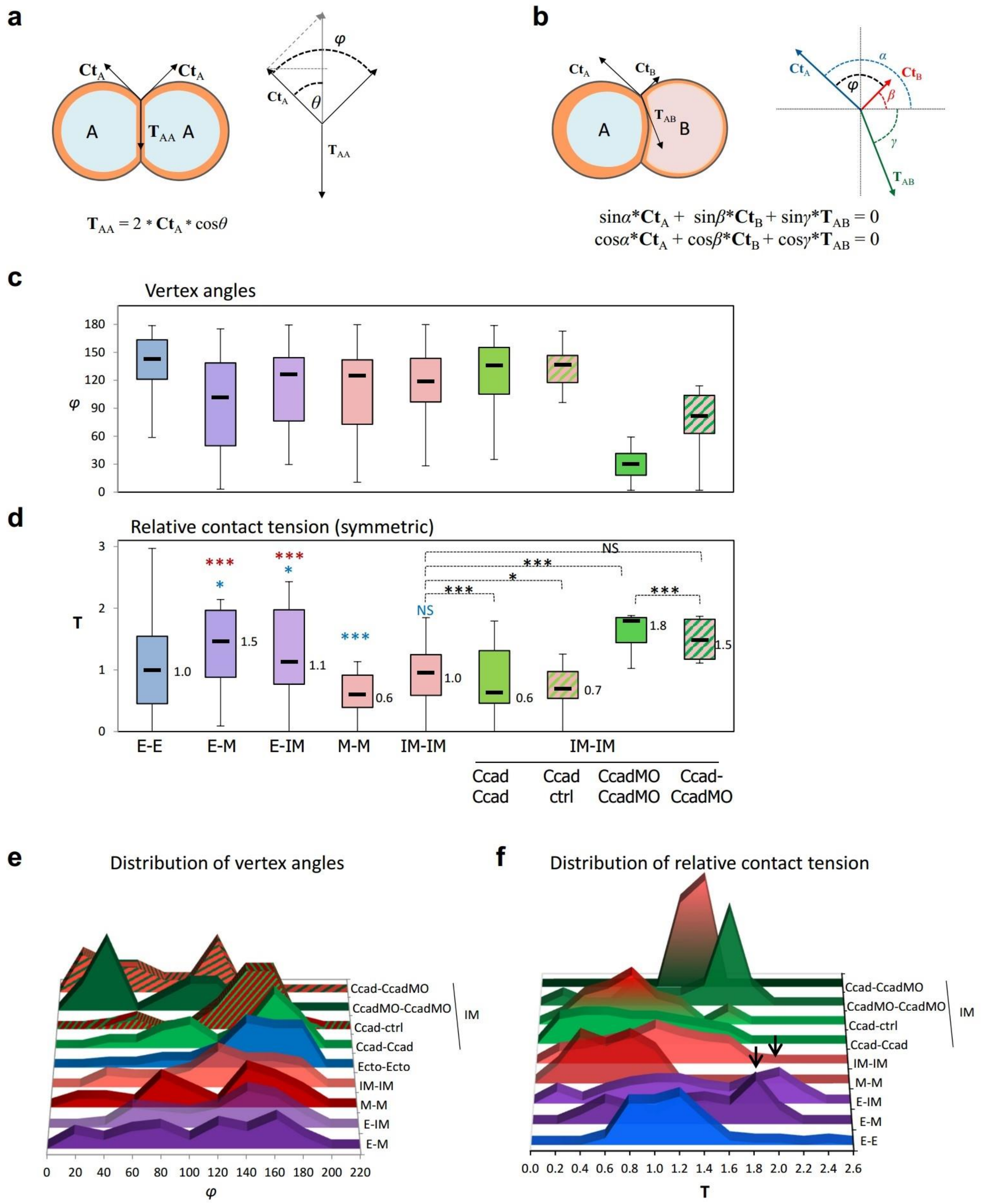
a
Ct_A
T_{AA}
A
φ
θ
$T_{AA} = 2 * Ct_A * \cos\theta$
b
Ct_B
T_{AB}
B
α
β
γ
$\sin\alpha*Ct_A + \sin\beta*Ct_B + \sin\gamma*T_{AB} = 0$
$\cos\alpha*Ct_A + \cos\beta*Ct_B + \cos\gamma*T_{AB} = 0$
c
Vertex angles
d
Relative contact tension (symmetric)
E-E
E-M
E-IM
M-M
IM-IM
Ccad Ccad
Ccad ctrl
CcadMO CcadMO
Ccad- CcadMO
e
Distribution of vertex angles
Ccad-CcadMO
CcadMO-CcadMO
Ccad-ctrl
Ccad-Ccad
Ecto-Ecto
IM
f
Distribution of relative contact tension
T

g

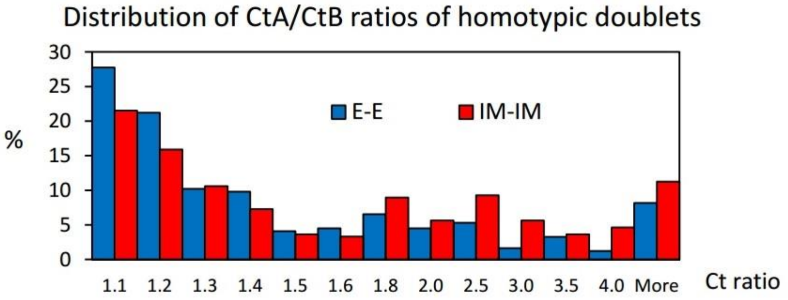
Distribution of CtA/CtB ratios of homotypic doublets
%
E-E
IM-IM
30
25
20
15
10
5
0
1.1
1.2
1.3
1.4
1.5
1.6
1.8
2.0
2.5
3.0
3.5
4.0
More
Ct ratio

h

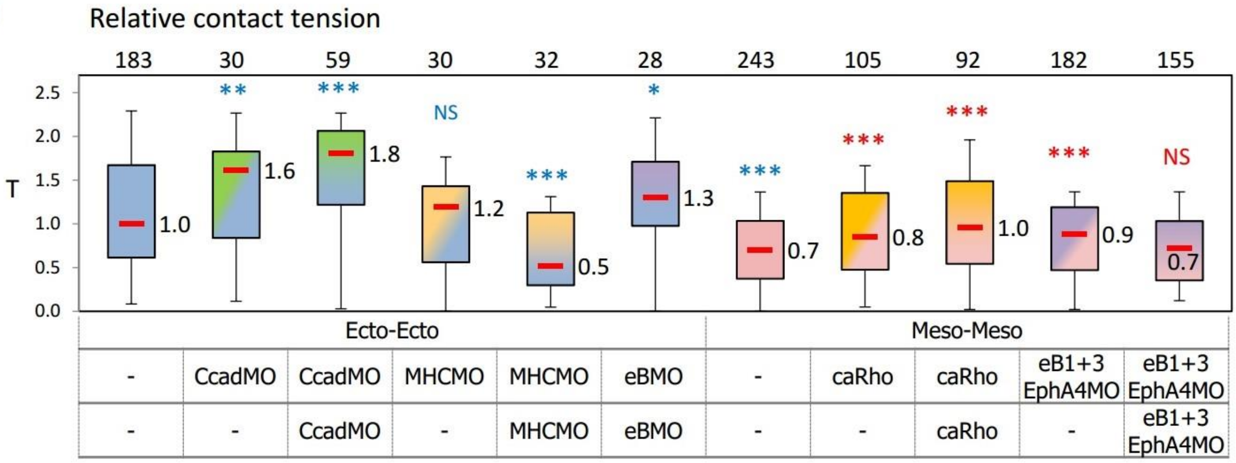
Relative contact tension
183 30 59 30 32 28 243 105 92 182 155
T
2.5
2.0
1.5
1.0
0.5
0.0
**
***
NS
***
*
***
***
***
***
NS
1.0
1.6
1.8
1.2
0.5
1.3
0.7
0.8
1.0
0.9
0.7
Ecto-Ecto
Meso-Meso
- CcadMO CcadMO MHCMO MHCMO eBMO - caRho caRho eB1+3 EphA4MO eB1+3 EphA4MO
- - CcadMO - MHCMO eBMO - - caRho - eB1+3 EphA4MO

i

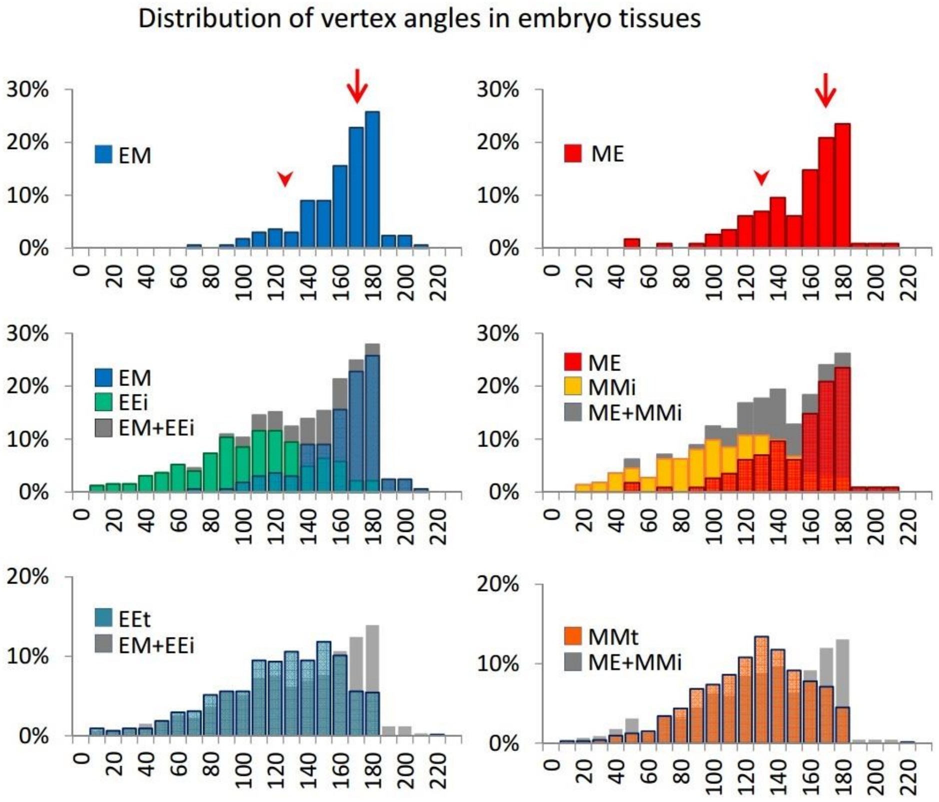
Distribution of vertex angles in embryo tissues
30%
20%
10%
0%
EM
ME
EM
EEi
EM+EEi
ME
MMi
ME+MMi
EEt
EM+EEi
MMt
ME+MMi
0
20
40
60
80
100
120
140
160
180
200
220

Supplementary Figure 3: Estimates of contact tension on cell doublets. (**a**) Simplified model of an ideal symmetric doublet of two cells with identical **Ct**. Contact tension $\mathbf{T}_{AA}$ is classically calculated based on **Ct** and on the angle θ, which is here half of φ, the angle formed by the two free cell surfaces at the contact vertex. (**b**) Model considering the asymmetry of cell doublets. The model considers doublet composed of two cells A and B with distinct cortical tensions $\mathbf{Ct}_A$ and $\mathbf{Ct}_B$. and a contact tension $\mathbf{T}_{AB}$. The direction of each force vector $\mathbf{Ct}_A$, $\mathbf{Ct}_B$, and $\mathbf{T}_{AB}$ is defined by the respective angle α, β or γ (φ was equal to α-β). The two equations expressing the equilibrium of the three forces were used to calculate the relative length of the three vectors. Even for homotypic doublets, relative **Ct** widely varied for each tissue (see graph panel g), consistent with high variations in the elastic modulus of single cells (main Fig.2e). Individual $\mathbf{Ct}_A$ and $\mathbf{Ct}_B$ were estimated using the median of the respective measured modulus and the $\mathbf{Ct}_A/\mathbf{Ct}_B$, the ratio calculated based on α, β, and γ. Relative contact tensions were all expressed relative to the median ectoderm homotypic tension, set to 1. Summary graphs and statistics are presented in the main Fig.3c. (**c**). φ measurements (symmetric model) for various doublet combinations (see main figure 3c). (**d**) Corresponding estimated relative contact tensions **T**. $\mathbf{Ct}_A$ is proportional to the measured elastic modulus (Fig.2e). For heterotypic doublets, $\mathbf{Ct}_A$ wasapproximated as the average of the ectoderm and mesoderm/IM **Ct**s. All tensions were expressed relative to the median value of the ectoderm homotypic contact tension, set at 1. Color code for statistical differences as in Fig.3c. (**e**) Histograms of angle φ distributions. (**f**) Distributions of relative T (asymmetric model, panel b and main Fig.3c). Arrows point to secondary peaks of heterotypic contacts with particularly high tension. (**g**) Quantification of heterogeneity in **Ct** between cells of homotypic doublet pairs. The model presented in panel b was used to calculate the ratio between the $\mathbf{Ct}_A$ and $\mathbf{Ct}_B$ for both ectoderm (E-E) and IM homotypic doublets. The number of doublets was 265 for E-E and 336 for IM-IM. The histogram presents the distribution of these ratios. In both cases many doublets showed a ratio larger than 1.5, and in 5-10% of the doublets, one of the cell was more than four times tenser than the other. (**h**) Effect of various manipulations on contact tension between cell doublets. Estimates considered the asymmetry of cell doublets. The conditions included depletion of C-cadherin (CcadMO), of myosin (MHCMO), and ephrinB1+3 (eBMO) in the ectoderm, caRho expression and ephrinB1+B3 (eB1+3) expression together with EphA4 depletion (A4MO) in the mesoderm. Note that the increased tension observed for ectoderm doublets upon ephrin depletion is consistent with previous observations of a pro-adhesive activity of ephrins in this tissue2. Color code for statistical differences as in main Fig.3c. (**i**) Estimates of contact tension in sections of whole embryos: Distributions of different types of angles, see main Fig.3d-g. Top histograms show the distributions of angles between heterotypic contacts. These distributions appear bimodal, with the main peak close to 180° (arrow), indicating high tension/low adhesion, and a second smaller peak closer to 120° (arrowhead), indicative of low tension similar to the tension within the tissues. The middle and bottom panels present respectively the distributions of angles formed by heterotypic and homotypic contacts at vertices bordering the boundary interface, and those formed by homotypic contacts within the two tissues. For comparison, we superimposed histograms that combine of all angles formed at boundary vertices (*EM+EE*i and *ME+MM*i). They emphasize the striking asymmetry at these vertices.

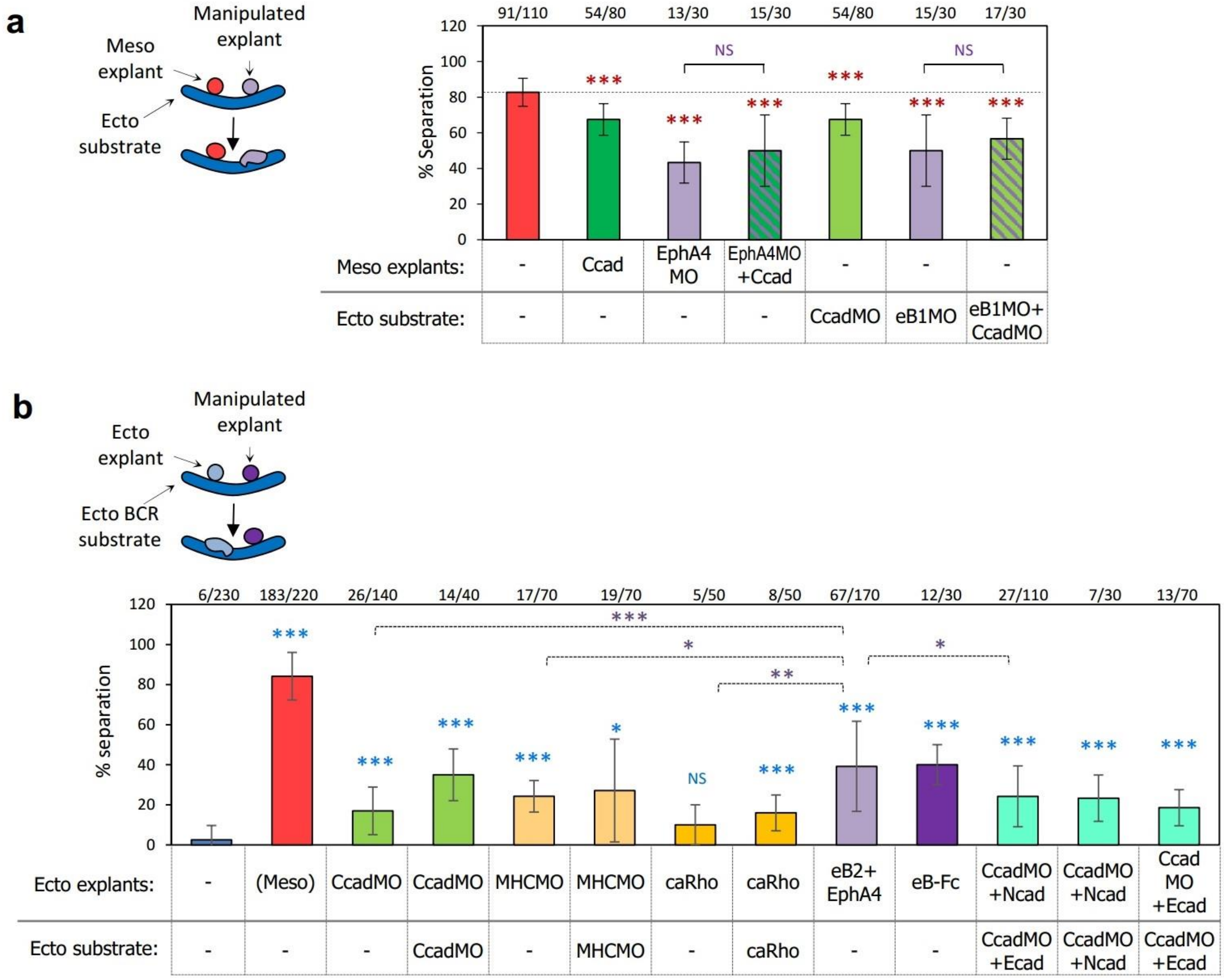

Supplementary Figure 4 | Effect of manipulations of myosin activity, cadherin levels, cadherin isoforms and ephrin-Eph signaling on separation. (**a**) Effect of combined manipulations on separation of mesoderm explants from ectoderm BCR. EphA4 depletion in the mesoderm inhibited separation by about 50%. Combining EphA4 depletion with C-cadherin overexpression (Cad) did not reinforce the phenotype, despite the fact that the latter manipulation should dampen adhesive differences between the two tissues. Similarly, inhibition of separation by ephrin depletion in the BCR was not increased by simultaneous cadherin depletion (CcadMO), another condition that should level adhesive differences. (**b**) Induction of ectoderm-ectoderm separation. Endogenous mesoderm explants were used as a positive control (red). Stimulation of ephrin-Eph signaling, either by expression of ephrinB2 and EphA4, which are normally expressed in the mesoderm, or by treatment of explants with soluble ephrin-Fc yielded the strongest separation. Decreasing cadherin levels (CcadMO), decreasing respectively increasing tension by myosin depletion (MHCMO) or caRho expression, or substituting C-cadherin with E- or N-cadherin all caused some degree of separation. However, in all cases, separation was at least as strong when the same manipulation was performed simultaneously on the explants and on the BCR, indicating that the effect was not due to tissue differences, unlike predicted by DAH, DITH or SAH. Graphs show mean values, error bars s.d. The numbers on top corresponds to the number of explants that remained separated/total explants (10 explants per independent replicate). Individual comparisons to control mesoderm, control ectoderm, or eprhinB2+EphA4 (respectively red, blue and purple asterisks) were done using one-sided Student's t-test.

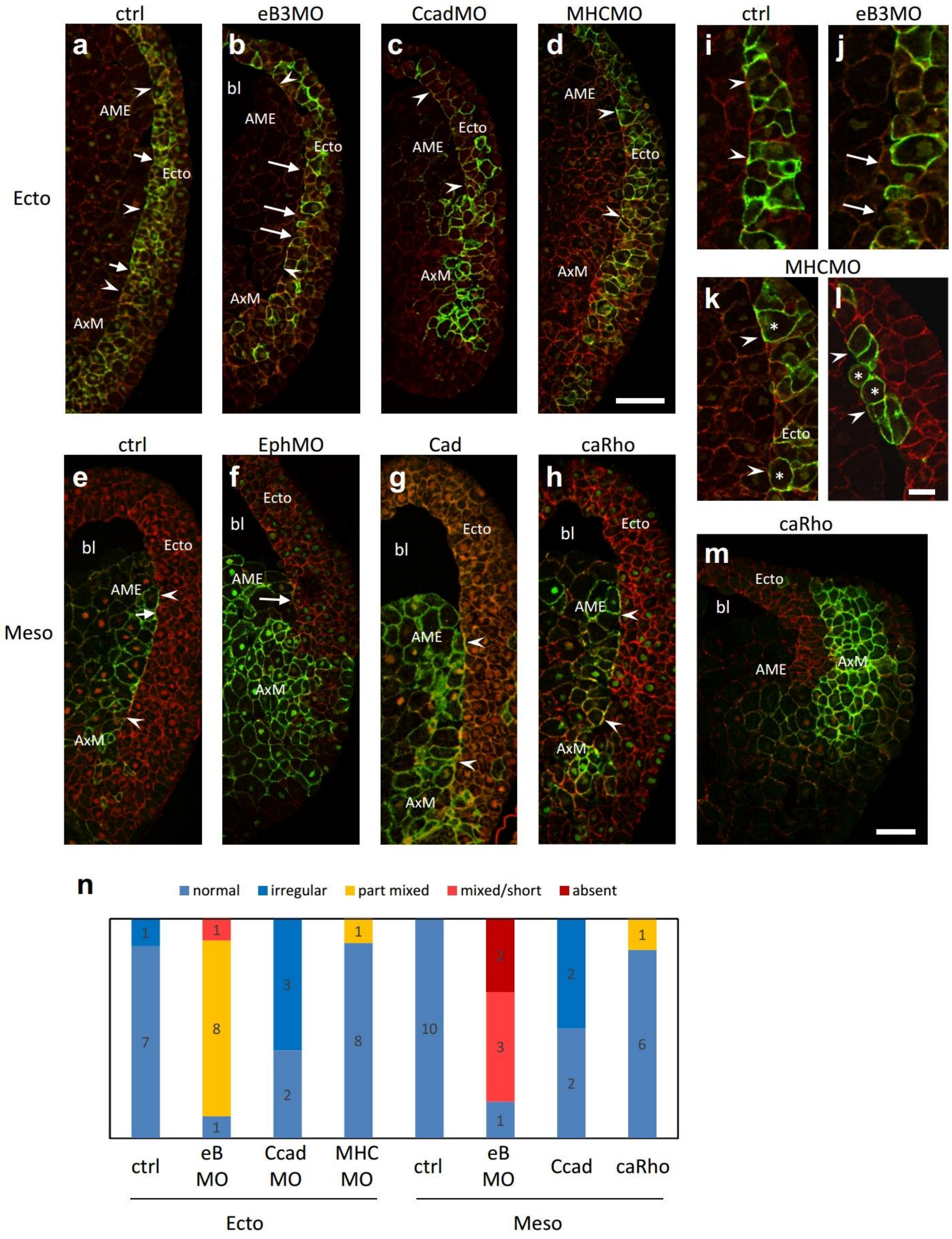

ctrl
eB3MO
CcadMO
MHCMO
ctrl
eB3MO
a
b
c
d
i
j
Ecto
AME
AxM
bl
MHCMO
k
l
ctrl
EphMO
Cad
caRho
e
f
g
h
Meso
caRho
m
n
normal
irregular
part mixed
mixed/short
absent
1
7
1
8
1
3
2
1
8
10
2
3
1
2
2
1
6
ctrl
eB MO
Ccad MO
MHC MO
ctrl
eB MO
Ccad
caRho
Ecto
Meso

Supplementary Figure 5 | Effect of targeted manipulations on the endogenous ectoderm-mesoderm boundary. Animal or dorsal-equatorial injections at the 4 to 8-cell stage allowed to preferentially target either the ectoderm or the mesoderm, respectively. Injected cells were marked by expression of membrane-targeted GFP. Sagittal cryosections from gastrula embryos were immunostained for GFP and β-catenin, used to visualize cell outlines and analyze the morphology of the boundary. Note that this analysis is less unambiguous than the explant-based assay, because the appearance of the boundary requires normal gastrulation movements, in particular mesoderm involution. Thus a boundary phenotype may not necessarily be due to inhibition of the process of separation per se, but to other gastrulation defects. Interfering with adhesion or myosin activity are likely conditions that may have such global effects. This is also the reason why we routinely perform the explant separation assay using mesoderm from the early gastrula dorsal lip, i.e. just before the start of involution, thus insuring that the exact same region is tested, irrespective of potential later defects in mesoderm migration. (**a-h**) Representative examples. Arrowheads point to the boundary. Arrows point to boundary irregularities/defects. The normal boundary is never perfectly smooth, but displays sparse indents (**a,e**, arrows), consistent with temporary phases of reattachment during the cycles of ephrin-Eph-dependent repulsion. Ephrin/Eph depletion led to boundary defects that ranged from partial mixing (**b**) to its complete absence (**f**). Dampening adhesive differences either by cadherin depletion in the ectoderm or its overexpression in the mesoderm had little effect on the boundary (**c,g**). Likewise, the boundary would still form in embryos with myosin depleted-ectoderm or with caRho-expressing mesoderm (**d,h**). The only instances where cadherin or myosin manipulations appeared to impact on the boundary were those where both ectoderm and mesoderm tissues had been simultaneously targeted, indicating that these defects were not related to DAH/DITH. Panel c shows an example where the upper part of the boundary is intact and corresponds to a region where GFP, marker for cadherin MO injection, is mostly found in the ectoderm, while in the lower part both ectoderm and mesoderm have received cadherin MO, and the boundary is completely absent. (**i-l**) Details of boundaries. Asterisks in k and l mark cells with different degrees of rounded shape reflecting loss of cell-cell adhesion. (**m**) Example of caRho-expressing embryo with severe gastrulation defects. caRho-expressing mesoderm has failed altogether to involute. Ecto, ectoderm; AME, anterior mesendoderm; AxM, axial mesoderm; bl, blastocoel. (**n**) Summary of boundary phenotypes, scored in categories of increasing severity. Partially mixed (part mixed) indicates that portions of the boundary were lost while others appeared intact (e.g. panel b). Numbers of embryos are indicated in the columns. Embryos in which both tissues had GFP signal and embryos showing obvious involution defects as in panel m were not included.

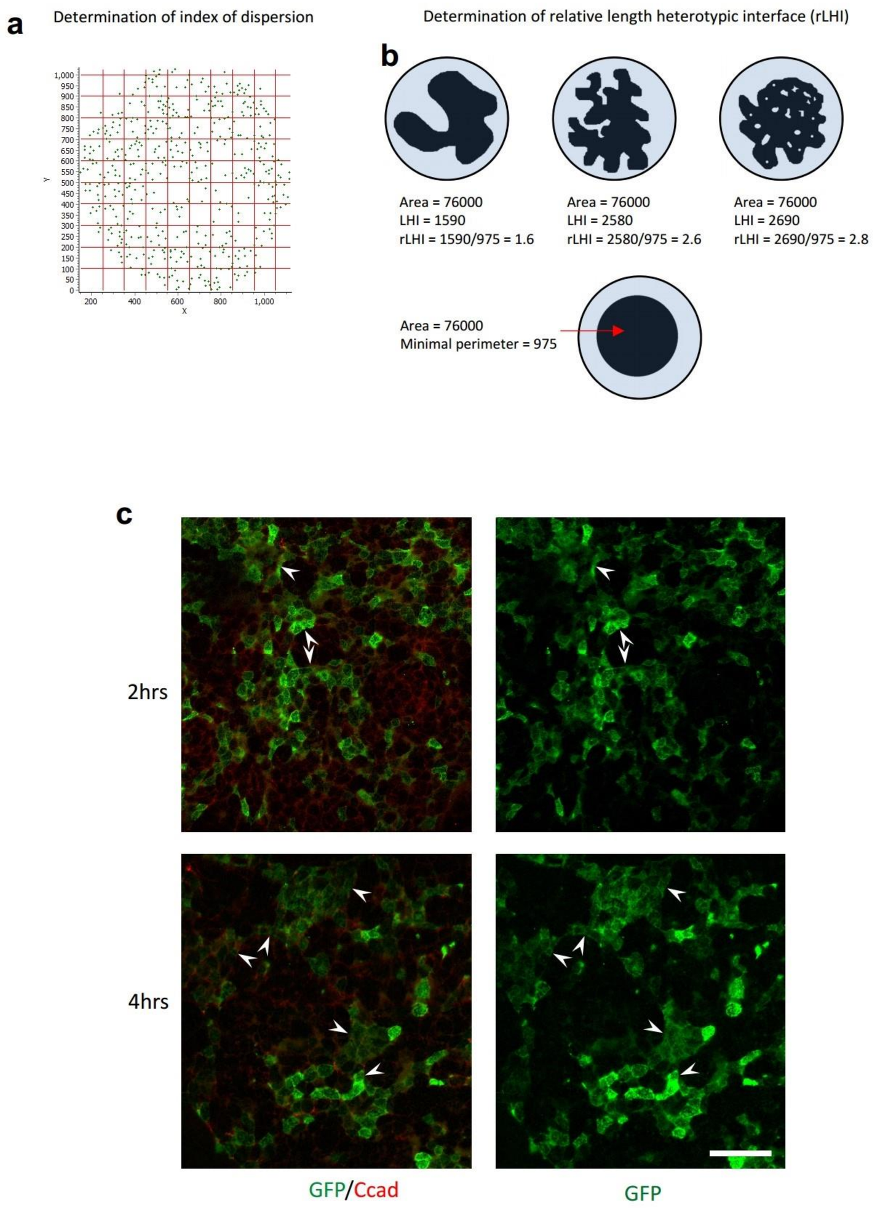

Supplementary Figure 6 | Reaggregation assay. (**a**) Example of grid used to measure the index of dispersion, here of a negative control aggregate. The green spots correspond to the positions of the labelled nuclei. (**b**) Principle of measurement of the relative length of heterotypic interface used to quantify the degree of separation in the reaggregation experiments of Fig.5: The diagrams represent symbolized aggregates (light blue circles) in which the labelled cells occupy the same area (black surface), but with different distributions. These illustrations show how LHI can be increased by irregular/convoluted interfaces (center) and by incomplete sorting of single or small groups of cells (right). Real aggregates varied in terms of size and of the relative number of cells of the two populations. In order to standardize their LHI, we calculated for each aggregate the theoretical minimal interface, i.e. the perimeter of a circle corresponding to the total surface of the labelled cells (lower diagrman). The relative LHI (rLHI) was defined as LHI divided by this minimal perimeter. Note that the complementary calculations based on the surface of the unlabeled cell population gave similar results (not shown). (**c**) Intermediate stages of reaggregation. Images of mixed ectoderm-IM aggregates fixed after 2 and 4 hrs. IM cells expressed GFP. Cryosections were immunostained for GFP (green) and β-catenin (red), as general marker of cell outlines. Left panel, merged images; right panels, green channel. Segregation starts by formation a small clusters, which will later coalesce in large groups. Even at these early stages, tissue segregation is detectable at a small scale by the presence of smooth heterotypic interfaces (arrowheads). Scale bar 200µm.

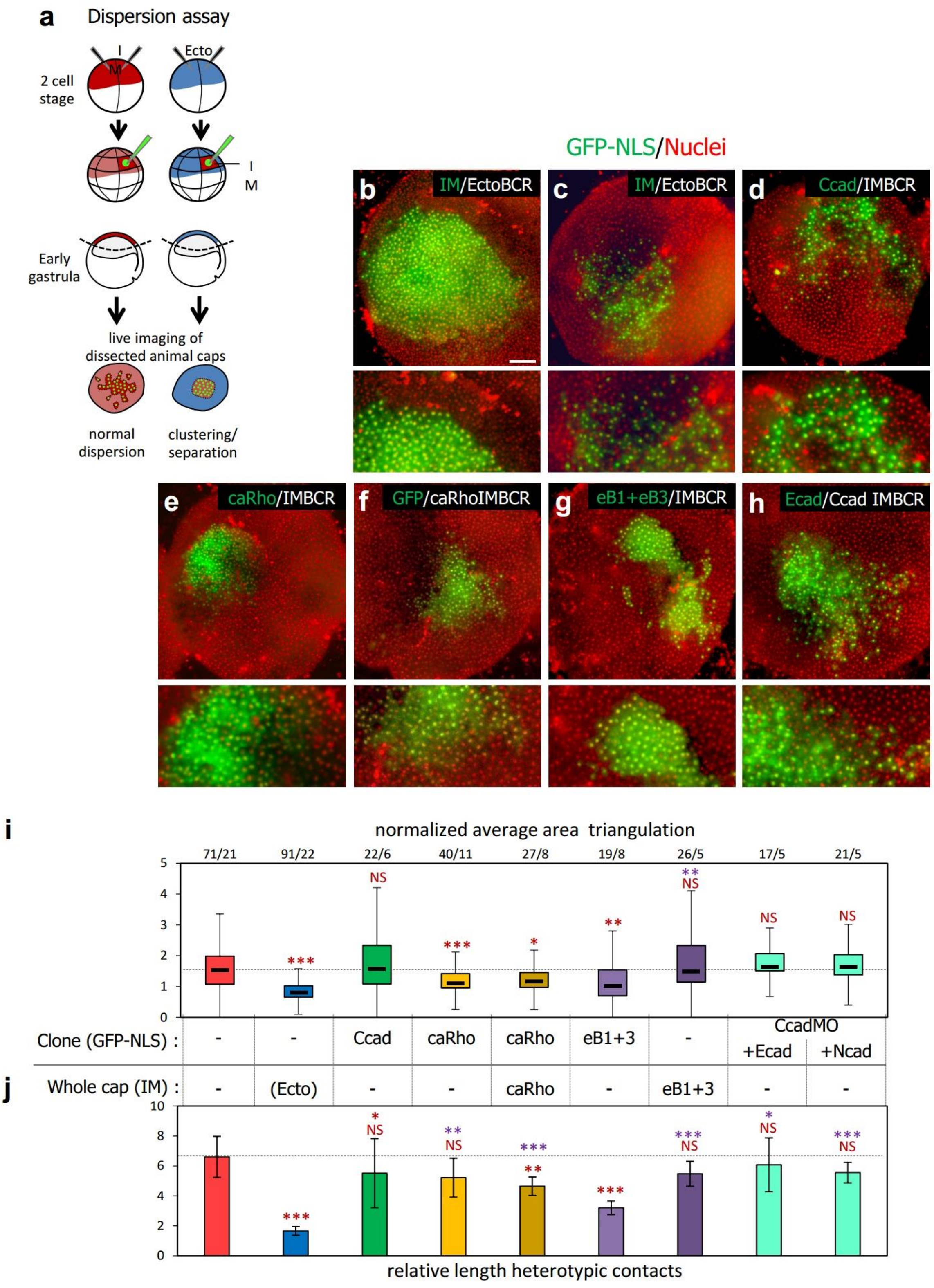
a
Dispersion assay
IM
Ecto
2 cell stage
Early gastrula
live imaging of dissected animal caps
normal dispersion
clustering/ separation
GFP-NLS/Nuclei
b IM/EctoBCR
c IM/EctoBCR
d Ccad/IMBCR
e caRho/IMBCR
f GFP/caRhoIMBCR
g eB1+eB3/IMBCR
h Ecad/Ccad IMBCR
i
normalized average area triangulation
71/21
91/22
22/6
40/11
27/8
19/8
26/5
17/5
21/5
Clone (GFP-NLS) :
Ccad
caRho
eB1+3
CcadMO
+Ecad
+Ncad
j
Whole cap (IM) :
(Ecto)
relative length heterotypic contacts

Supplementary Figure 7 | Dispersion assay. (**a**) Diagram of the assay. A clone of experimentally manipulated cells was produced within the BCR by injection of a single blastomere at the 32-cell stage. The distribution of the labelled progeny was determined at the early gastrula stage when endogenous separation occurs. (**b-h**) Representative examples. Live fluorescence images. Nuclei were stained with Hoechst, pseudocolored in red. Lower panels are enlargements of part of the main image. (**i**) Quantification of dispersion using Delaunay triangulation and represented as a Tukey boxplot. Comparisons to mesoderm (red) and to ephrin expression (purple) were done using one-sided Student's t-test. Numbers above, total number of BCRs. (**j**) Quantification of the relative heterotypic contact length. (**b**) When the clone was induced to a mesodermal fate, cells formed a tight cluster sharply delineated from the surrounding ectodermal BCR. (**c**) When the entire BCR was induced to mesoderm, the labelled clone was broadly dispersed. (**d-h**) Manipulation of a clone within an IMBCR. (**d**) C-cadherin overexpression had no effect. (**e,f**) caRho expression impaired dispersion (**e**), but a similar effect was observed when caRho was expressed throughout the IMBCR (**f**), indicating that the effect was not related to a difference in tension between the clone and the surrounding cells, but most likely to interference with intercellular migration. (**g**) Clones of ephrinB1/B3-expressing mesoderm cells remained confined in a compact area of the IMBCR. Note that control expression of the same ephrins throughout the entire IMBCR led to normal dispersion, demonstrating that, unlike caRho, ephrin expression did not impair intercellular migration, but generated a genuine process of cell sorting (**i,j**). (**h**) Replacement of C-cadherin with E- or N-cadherin had no effect. Scale bar, 200µm.

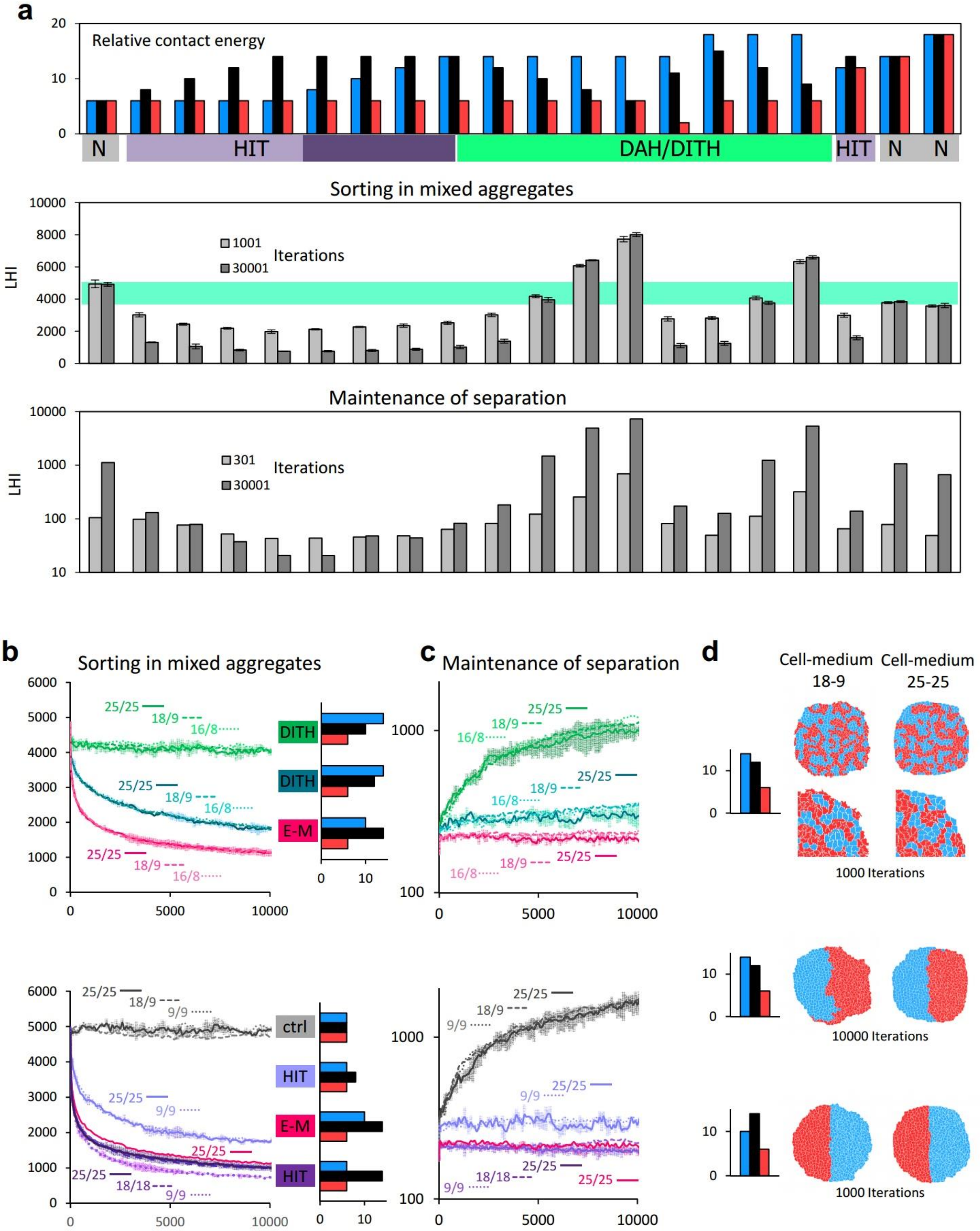
a
Relative contact energy
N
HIT
DAH/DITH
HIT
N
N
Sorting in mixed aggregates
LHI
1001
30001
Iterations
Maintenance of separation
301
30001
Iterations
b
Sorting in mixed aggregates
DITH
DITH
E-M
ctrl
HIT
E-M
HIT
c
Maintenance of separation
d
Cell-medium 18-9
Cell-medium 25-25
1000 Iterations
10000 Iterations
1000 Iterations

Supplementary Figure 8 | Simulation of cell sorting and of maintenance of separation.
(**a**) Systematic comparison of different situations. Top: Relative energies, corresponding to contact tensions, organized according to the different models: HIT, with identical homotypic tension and higher heterotypic tension; HIT with different homotypic tensions, comparable to the situation for endogenous ectoderm and mesoderm; DITH, with differences in tension between the two cell populations, and an intermediate heterotypic tension; N, negative control conditions, all energies set identical. Middle and bottom graphs: Results for sorting from mixed aggregates and maintenance of separation. For mixed aggregates, values below the green stripe indicate significant shortening of LHI, thus separation. Some conditions led to LHI lengthening, thus increased dispersion. They all belonged to the DITH category and were characterized by a heterotypic tension set closer to the lowest homotypic tensions. For maintenance of separation, a low increase in LHI corresponded to efficient maintenance of separation. Larger increases corresponded to decreased boundary straightness and cell mixing. (**b-d**) Effect of cell to medium energies. These energies represent the cortical tension at free cell surfaces (Ct). (**b-c**) LHI curves for sorting and maintenance of segregation for selected conditions. Contact energy values are shown as bar graphs. Cell to medium energies are indicated next to the curves. Two types of setting were used: equal (25, 18 and 9) or unequal energies (18/9 and 16/8). HIT scenarios were only given equal energies. Note that while estimates from ectoderm-ectoderm and IM-IM doublets indicated that Cts tended to be close to T, a clear relationship could not be established due to the large variability of individual Ct/T ratios. Curves are averages from three independent simulations. For clarity error bars (s.d.) are only shown for selected conditions. The condition ectoderm-mesodem (E-M) with 25/25 values was included in all graphs to help visual their comparison. (**d**) Examples of snapshots for sorting and maintenance of separation. Varying cell to medium energies had mainly an influence on the global shape of the aggregate, which became more irregular when the energies was lowered. However cell sorting was only marginally affected, as shown by the close matching of the LHI curves.

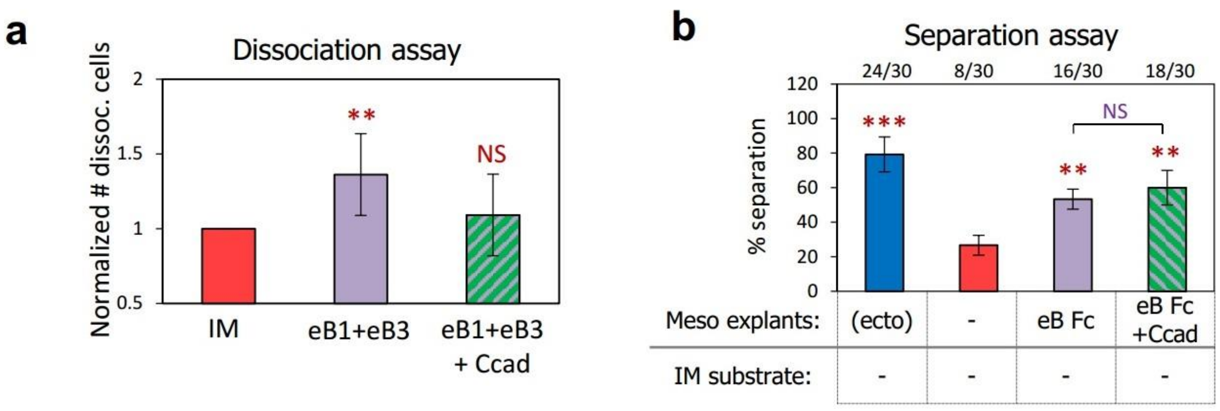

Supplementary Figure 9 | Combined effects of cadherin expression and ephrin signaling.
(**a**) Dissociation assay showing decreased cell-cell adhesion for mesoderm explants upon ephrin ectopic expression and rescue by C-cadherin coexpression. Results are average from eight independent experiments. (**b**) Separation of mesoderm tissues induced by treatment of the explants with soluble ephrin-Fc is not sensitive to C-cadherin overexpression. Numbers on top correspond to separated/total number of explants, from three experiments. Student's t-test, color code as above.

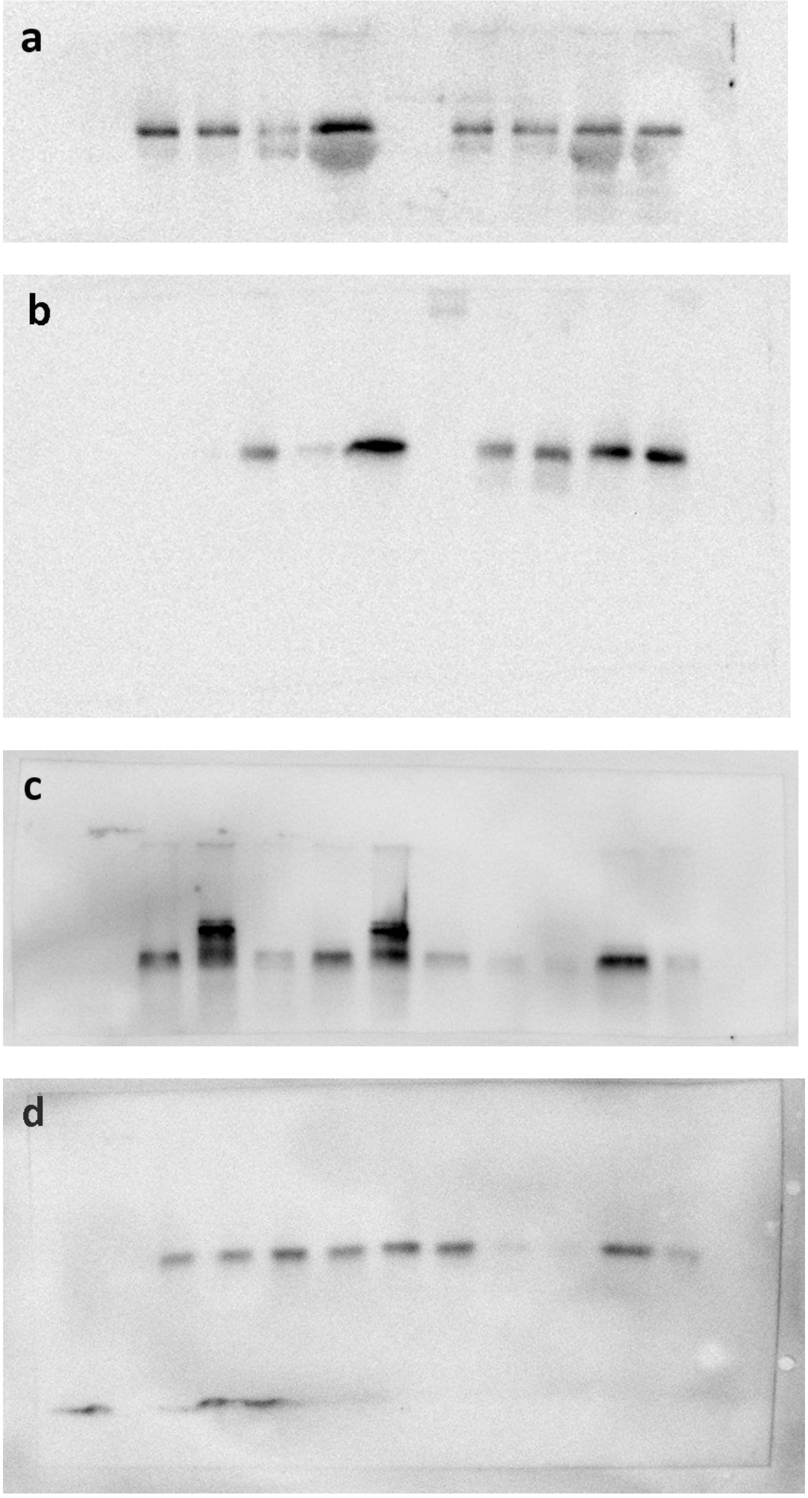

Supplementary Figure 10 | Uncropped immunoblots. Membranes transferred from a single gel were cut horizontally to probe simultaneously the top part for cadherin and the bottom part for GAPDH. (**a**) Cadherin, Fig.2c. (**b**) GAPDH, Fig.2c. (**c**) Cadherin, supplementary Fig.2a. (**d**) GAPDH, supplementary Fig.2a.

Supplementary Table 1 | List of morpholinos

Morpholino	Sequence	Amount injected at 2 cell stage (ng)
Control	5'-CCTCTTACCTCAGTTACAATTTATA	20
C-Cadherin	5'-CCACCGTCCCGAACGAAGCCTACAT	20
ephrinB1	5'-GGAGCCCTTCCATCCGCACAGGTGG	20
ephrinB2	5'-ACACCGAGTCCCCGCTCAGTGCCAT	20
EphA4	5'-AGATGCCATGTACAATCCCAGCCAT	20
EphB4	5'-ACAGGAGGAGGAGCCAGAGATCCAT	20
Myosin Heavy Chain 2A	5'-GATACTTGTCCACATCTGTTTGTGC	20
Myosin Heavy Chain 2B	5'-CTTCCTGCCCTGGTCTCTGTGATCAT	20

Supplementary Table 2. List of mRNA

mRNA	mRNA injected per blastomere at 2 cell stage (pg)
caActR	1000
β-Catenin	100
C-Cadherin	1000
E-Cadherin	1000
N-Cadherin	1000
EphrinB1	250
EphrinB2	250
EphrinB3	500
EphA4	500
RhoAV (caRho)	100
RhoAN (dnRho)	100
GAP-GFP	100
GAP-Cherry	100
NLS-YFP	100
β-Gal	1000

Supplementary Table 3. List of primers used for RT-PCR

Gene	Sequence	Temp (°C)	Cycles	Reference
ODC	For:5'-GTCAATGATGGAGTGTATGGATC<br>Rev:5'-TCCATTCCGCTCTCCTGAGCAC	61	23	3
Brachyury	For: 5'-GGATCGTTATCACCTCTG<br>Rev:5'-GTGTAGTCTGTAGCAGCA	53.7	27	4
Cerberus	For: 5'-GCTGAACTATTTGATTCCACC<br>Rev:5'-ATGGCTTGTATTCTGTGGGGC	62	28	5
Chordin	For: 5'-ATGCAGTGTCCCCCCATC<br>Rev:5'-GCAGTGCATAACTCCGAA	55	27	---
Goosecoid	For: 5'-ACAACTGGAAGCACTGGA<br>Rev:5'-TCTTATTCCAGAGGAACC	52	27	5
Sox17	For: 5'-GGACGAGTGCCAGATGATG<br>Rev:5'-CTGGCAAGTACATCTGTAC	57	25	6

Supplementary Material: Mathematical simulations

Calculation of relative contact tensions

Estimates of relative contact tensions were based on the principle that the geometry of the cell membranes at cell vertices reflects the equilibrium between the tensile forces exerted by the cell cortices. For a doublet formed of cell A and cell B (Supplementary Fig.3b), the equilibrium involved the cortical tensions at the two free cell surfaces ($\mathbf{Ct}_A$ and $\mathbf{Ct}_B$) and the contact tension at cell-cell interface ($\mathbf{T}_{AB}$). Note that $\mathbf{T}_{AB}$ is defined as the sum of the cortical tensions of each cell at the contact ($\mathbf{Ct}_{A'}$ and $\mathbf{Ct}_{B'}$) and of the negative contribution due to cell-cell adhesion (-ω). Note also that cadherin adhesions decrease myosin contractility along cell-cell contacts [7], thus $\mathbf{Ct}_{A'}<\mathbf{Ct}_A$ and $\mathbf{Ct}_{B'}<\mathbf{Ct}_B$). For the purpose of the present calculations, however, $\mathbf{T}_{AB}$ could be considered as a whole, without the need to dissect it into its tensile and adhesive components.

The force equilibrium was expressed by two equations:

(1) $\sin a * \mathbf{Ct}_A + \sin\beta * \mathbf{Ct}_B + \sin\gamma * \mathbf{T}_{AB} = 0$

and

(2) $\cos a * \mathbf{Ct}_A + \cos\beta * \mathbf{Ct}_B + \cos\gamma * \mathbf{T}_{AB} = 0$

Angles a, β and γ corresponded to the orientation of each force vector. Each of these angles was measured as the tangent to an arc fitted to the cell membrane at the cell vertex.

Based on equations (1) and (2), we could use the three angles to calculate the ratios between the contact tension and each of the cortical tensions:

(3) $\mathbf{T}_{AB}/\mathbf{Ct}_A = (\sin\beta - \sin a * \cos\beta/\cos a)/(\sin a * \cos\gamma/\cos a - \sin\gamma)$

and

(4) $\mathbf{T}_{AB}/\mathbf{Ct}_B = (\sin a - \sin\beta * \cos a/\cos\beta)/(\sin\beta * \cos\gamma/\cos\beta - \sin\gamma)$

These two ratios also allowed us to calculate the relative ratio between $\mathbf{Ct}$s:

(5) $\mathbf{Ct}_A/\mathbf{Ct}_B = (\mathbf{T}_{AB}/\mathbf{Ct}_B)/(\mathbf{T}_{AB}/\mathbf{Ct}_A)$

To compare the tensions between different types of doublets, we calculated a relative contact tension rel$\mathbf{T}$ (abbrevatiated simply $\mathbf{T}$ in the text and legends), which took into account the measured elastic modulus (E) corresponding to each cell type.

AFM measurements had shown the E of individual cells was highly variable, which was confirmed by the measurements of the ratio $\mathbf{Ct}_A/\mathbf{Ct}_B$ of homotypic doublets ($\mathbf{Ct}_A/\mathbf{Ct}_B$ was higher than 2-fold in respectively 18%, 16% and 33% of ectoderm, mesoderm and IM doublets). The E of each cell of a doublet being unknown, we made the assumption that the average of the cortical tensions of two cells of a homotypic doublet $(\mathbf{Ct}_A+\mathbf{Ct}_B)/2$ was proportional to the median of measured E.

Relative contact tension rel$\mathbf{T}_{AB}$ was calculated with the equation:

(6) $\text{rel}\mathbf{T}_{AB} = 2*E_A*(\mathbf{T}_{AB}/\mathbf{Ct}_A)/(1+((\mathbf{T}_{AB}/\mathbf{Ct}_A)/(T_{AB}/\mathbf{Ct}_B)) + 2*E_B*(\mathbf{T}_{AB}/\mathbf{Ct}_B)/(1+((\mathbf{T}_{AB}/\mathbf{Ct}_B)/(T_{AB}/\mathbf{Ct}_A))$

All rel$\mathbf{T}$s were then expressed relative to the rel$\mathbf{T}$ of ectoderm homotypic doublets
(median (rel$\mathbf{T}_{E\text{-}E}$) = 1.0).

We used the same principle of force equilibration to estimate relative tensions within the embryo tissues (Fig.3d-g). Here the three force vectors corresponded to the three contact tensions acting at the vertex. The equations (3),(4) and (5) were similarly used to calculate the three relative tensions at vertices within the tissue (all homotypic contacts, either $\mathbf{T}_{EEt}$ or $\mathbf{T}_{MMt}$), and at vertices abutting the ectoderm-mesoderm boundary (two heterotypic contacts and one homotypic contact, either T_{EM} and $\mathbf{T}_{EEi}$, or $\mathbf{T}_{ME}$ and $\mathbf{T}_{MMi}$).

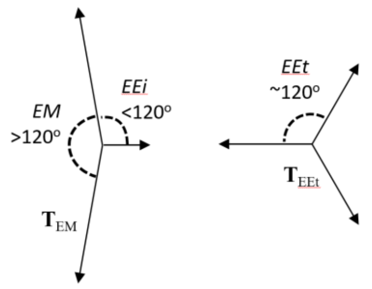

The calculated tension ratios for homotypic contacts within the tissues were close to 1.0, consistent with a relatively homogenous force distribution in each tissue. We found a small but significant difference between the medians of the relative tensions at heterotypic contacts ($\mathbf{T}_{EM}$ and $\mathbf{T}_{ME}$), which indicated that the ectoderm was more tensile than the mesoderm. The ration between the two homotypic tensions was estimated based on the equations:

(7) $\mathbf{T}_{MM} = 2*\cos(ME/2)*\mathbf{T}_{ME}$ and

(8) $\mathbf{T}_{EE} = 2*\cos(EM/2)*\mathbf{T}_{EM}$

Given that $\mathbf{T}_{ME} = \mathbf{T}_{EM}$:

(9) $\mathbf{T}_{MM}/\mathbf{T}_{EE} = \cos(EM/2)/\cos(ME/2)$

By inputting the median values of angles *EM* and *ME*, we obtained:
$\mathbf{T}_{MM}/\mathbf{T}_{EE} = \cos(78.4^{o})/ \cos(81.4^{o}) = 0.20/0.15 = 1.34$

Simulations using a modified Potts Model
Principle and settings

The model was based on a Markov chain Monte Carlo method, where the overall energy of the system is calculated at all iterations. Cells are connected domains of pixels on a lattice that evolves according to a set of probabilistic rules. Transition probabilities from one configuration to the next are dictated by a Hamiltonian, a function that associates an energy to each configuration. This function is a sum of energies resulting from cell-cell contact energies, cell-medium contact energies and deviations from cell size constraints (target area). The energy is highest at the cell-to-medium interface (only cortical contractility, no adhesion), and lowest for contacts with high adhesion/low myosin contractility. At each iteration, one random pixel changes identity and the resulting energy of the system is calculated. Changes that result in lower energy (more favourable state) are accepted in all cases while those that increase the energy are accepted with a probability based on $\Delta E/T$, where ΔE= the change in energy and T = temperature, see below. We devised a custom framework written in Java to run the simulations featured in this paper.

We defined parameters that remained constant in all conditions, which included the target area (40 pixels), a cell-to-medium contact energy (set at 25 unless stated otherwise, see legends and supplementary Fig.8), and temperature (representative of cell motility) set to 10. For simulation of cell sorting from a mixed aggregate, the starting matrix was produced by first running a short simulation for a chessboard matrix where all cell-cell contacts had identical energies, which led to a round "aggregate" with irregular cell shapes. Cell identity was then allocated randomly on this matrix. To produce replicated simulations, independent random matrices were prepared for each replicate. Each simulation was run for at least 30000 Monte Carlo steps, at which point sorting had largely plateaued. The results were then calculated as the average of the three parallel simulations. For simulation of maintenance of separation, the initial matrix was made of a round aggregate split in two halves by a straight boundary.

Description of the cellular Potts model and implementations

The *cellular Potts model* (CPM) or *Glazier-Graner-Hogeweg model* is an adaptation of an equilibrium model from ferromagnetic statistical physics to seemingly mimic biological patterns observed during development [8,9]. The authors modified the Metropolis algorithm, a time-discrete Markov chain Monte Carlo method, to drive the system from an initial arbitrary state towards a stationary state whereby macroscopic scale patterns emerge from the local and interdependent interactions of individual units. In this section, we summarize the Metropolis algorithm used by Glazier and Graner in their original papers [8,9] and then describe how we implemented it in our code.

1. Cellular and Subcellular Structures

The CPM is a two-dimensional $N \times M$ lattice model that evolves by updating *pixel* $s_{i,j}$ properties, also termed *node* or *site*, according to probabilitic rules ($< i,j >= x \in S$, $|S| = MN$). A biologiacl *cell* c_k is represented by a domain of simply connected pixels with the same cell index $k \in W = \{0,1,...,n\}$. The cell index 0 is assigned to a pixel if it belongs to medium.

Cells of different *types* $t \in \Lambda$ undergo membrane fluctuations by expanding or shrinking their volume $A(c_k)$ and surface length $P(c_k)$[1]. A cell increases its volume by occupying an adjacent lattice site while simultaneously decreasing a neighbouring cell's volume by one pixel. This occupation of a lattice site by a cell is designated as a *spin copy*, a term borrowed from the original ferromagnetic Potts model.

2. Dynamics and Hamiltonian

The state of the system is described by configurations $\eta \in X = WS$ each corresponding to a many- to-one mapping of lattice sites to cell indices. Transitions probabilities from one configuration to the other are dictated by a *Hamiltonian*, a function $H(\eta)$ that associates an *energy* to each configuration. This function is the sum of several terms controlling single aspects of the cells' interdependence structure. The CPM-Hamiltonian proposed by Glaner and Grazier uses two terms: the cell-type-dependent *surface interaction energy* $H^S(\eta)$ and the *volume constraint* $H^A(\eta)$ (Supplementary Table 4). In addition, many CPM extensions include a surface length constraint $H^P(\eta)$ {Glazier, 1993 #2328}.

Supplementary Table 4

ENERGY	EQUATION	DESCRIPTION
Cell volume energy	$h^A(c)=\delta_A \cdot (a_0 - a)^2$	where a is cell c's volume, a_0 is the target cell volume and δ_A is the strength or *elastic- ity* of the volume constraint
Volume energy	$H^A(\eta) = \sum_{c_k \in W} h^A(c_k)$	This function is the sum of volume energies for all cells
Cell surface length energy	$h^P(c) = \delta_P \cdot (p_0 - p)^2$	where p is cell c's surface length, p_0 is the target surface length and δ_P is the strength or *elasticity* of the surface length constraint
Surface length energy	$H^P(\eta) = \sum_{c_k \in W} h^P(c_k)$	This function is the sum of surface length energies for all cells.
Pixel interaction energy	If $c(s) = c(s')$, $J(s, s') = 0$<br>Otherwise, $J(s, s') = j(\tau(s), \tau(s'))$	$\tau(s)$ corresponds to pixel s's cell type and $c(s)$ to its cell index. = $j(t, t')$corresponds to the interaction energy value between cell types t and t'
Surface interaction energy	$H_S(\eta) = \sum_{s,s'\ neighbors} J(s,s')$	This function is the sum of individual interaction energies between pixel neighbors.

[1] Given that it is a 2D model, in our code, we use the term « surface » to describe the cell volume and « perimeter » to describe the cell surface.

3. The modified Metropolis algorithm

Minimal version:

Initialize a $M \times N$ lattice with random configuration η;
For each *Monte Carlo Step* **do**
    for *count:* $M \times N$ **do**
    Pick a random pixel s;
    Amongst s's neighbors, pick a random pixel s';
    Compute $\Delta H = H(\eta') - H(\eta)$ where η' is the configuration resulting from *copying* s onto s';
    Set $\eta := \eta'$ with probability P as defined in equation 1 or ignore the transition with probability $1 - P$;
    end
end

Description of the algorithm

For each Monte Carlo step (MCS), a number of spin copies is attempted[2]. At each spin copy attempt, a random pixel s is chosen along with one of its random neighbors s'. The change in energy ΔH resulting from s' adopting the cell index of s (i.e. s *copying* itself onto s') is then evaluated and the spin copy attempt is accepted according to a Boltzmann transition probability P dependent on temperature $T > 0$ (Eq. 1, Algorithm 1). The temperature parameter T is the biological analogue of the energy thermal fluctuations in statistical physics and may be used as a measure of cell motility. In our simulations, the temperature is constant.

Equation 1: a spin copy acceptance function

$$\begin{cases} \Delta H \leq 0 : P = 1 \\ \Delta H > 0 : P = e^{\frac{-\Delta H}{T}} \end{cases} \qquad \text{where } T = \text{temperature}$$

This spin copy acceptance function ensures that unfavorable changes may be kept all the while driving the system to adopt an energetically minimal stationary configuration (at least, locally minimal). The acceptance of unfavorable changes is crucial in the intermediate steps of cell sorting since it enables the system to cross potential energy barriers separating two minima of energy.

[2] In the Glazier-Graner CPM, $16 \times M \times N$ spin copies are attempted per MCS. However, in our simulations, only $M \times N$ spin copies are attempted, 16 being perceived to be an arbitrary factor.

Supplementary References

1 Wacker, S., Grimm, K., Joos, T. & Winklbauer, R. Development and Control of Tissue Separation at Gastrulation in Xenopus. *Dev Biol* **224**, 428-439 (2000).
2 Rohani, N., Canty, L., Luu, O., Fagotto, F. & Winklbauer, R. EphrinB/EphB signaling controls embryonic germ layer separation by contact-induced cell detachment. *PLoS Biol* **9**, e1000597 (2011).
3 Schohl, A. & Fagotto, F. A role for maternal beta-catenin in early mesoderm induction in Xenopus. *EMBO J* **22**, 3303-3313 (2003).
4 Rupp, R. A., Snider, L. & Weintraub, H. Xenopus embryos regulate the nuclear localization of XMyoD. *Genes Dev* **8**, 1311-1323 (1994).
5 Bouwmeester, T., Kim, S., Sasai, Y., Lu, B. & De Robertis, E. M. Cerberus is a head-inducing secreted factor expressed in the anterior endoderm of Spemann's organizer. *Nature* **382**, 595-601 (1996).
6 Hudson, C., Clements, D., Friday, R. V., Stott, D. & Woodland, H. R. Xsox17alpha and -beta mediate endoderm formation in Xenopus. *Cell* **91**, 397-405 (1997).
7 Winklbauer, R. Cell adhesion strength from cortical tension - an integration of concepts. *J Cell Sci* **128**, 3687-3693 (2015).
8 Graner, F. & Glazier, J. A. Simulation of biological cell sorting using a two-dimensional extended Potts model. *Physical review letters* **69**, 2013-2016 (1992).
9 Glazier, J. A. & Graner, F. Simulation of the differential adhesion driven rearrangement of biological cells. *Phys Rev E Stat Phys Plasmas Fluids Relat Interdiscip Topics* **47**, 2128-2154 (1993).
